# Late-Stage C–H Acylation of Tyrosine-Containing Oligopeptides with Alcohols

**DOI:** 10.1021/acs.orglett.1c02764

**Published:** 2021-09-03

**Authors:** Iñaki Urruzuno, Paula Andrade-Sampedro, Arkaitz Correa

**Affiliations:** † Department of Organic Chemistry I, University of the Basque Country (UPV/EHU), Joxe Mari Korta R&D Center, Avenida Tolosa 72, 20018 Donostia-San Sebastián, Spain; ‡ Donostia International Physics Center (DIPC), Paseo Manuel de Lardizabal 4, 20018 Donostia-San Sebastián, Spain

## Abstract

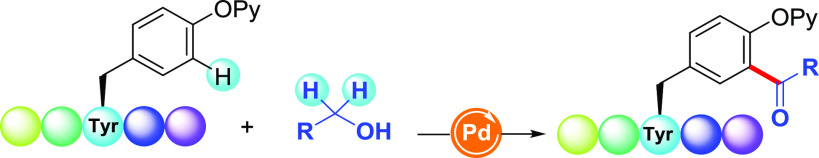

The selective tagging of amino acids within a peptide framework while using atom-economical C–H counterparts poses an unmet challenge within peptide chemistry. Herein, we report a novel Pd-catalyzed late-stage C–H acylation of a collection of Tyr-containing peptides with alcohols. This water-compatible labeling technique is distinguished by its reliable scalability and features the use of ethanol as a renewable feedstock for the assembly of a variety of peptidomimetics.

Since the definition of the “12 Principles of Green Chemistry” by Anastas and Warner in 1998,^1^ sustainable development represents a major global concern when designing new chemical synthetic processes.^2^ As a result, the last decades have witnessed the upsurge of a sheer number of greener and safer procedures for the synthesis of fine and commodity chemicals. In particular, the practical use of abundant and renewable carbon feedstock in the realm of organic chemistry has gained considerable attention.^3^ However, the use of ethanol as a valuable and cheap C_2_ feedstock is still rare, and it is chiefly used as an organic solvent rather than an actual coupling partner.^4^ In this communication, we unlock its synthetic versatility and advantageous features within the burgeoning field of bioconjugation.

Owing to their unique biological activities and improved metabolic stability compared to their native compounds, synthetically modified peptides are of utmost importance in the field of proteomics, chemical biology, and drug discovery.^5^ Metal catalysis has recently emerged as an enabling tool for the manipulation of typically unreactive C–H bonds embedded within the amino acid backbone^6^ and the corresponding side chains.^7^ Accordingly, metal-catalyzed C–H functionalization techniques are becoming highly embraced by mainstream synthetic chemists because they enable the straightforward assembly of biomolecules in a sustainable fashion.^8^ Despite the existing palette of reactivity, most of the protocols entail the use of toxic halide counterparts and feature the modification of highly reactive amino acid residues. Therefore, innovative tactics are highly coveted to forge peptides beyond those found in naturally occurring proteins, and the usage of new atom-economical C–H coupling partners to label less reactive and poorly nucleophilic handles represents an ideal strategy in these endeavors. The modification of peptides housing hydrophobic phenylalanine (Phe) and tyrosine (Tyr) residues remains comparatively overlooked,^9^ which is clear evidence that the direct translation of a given C(sp^2^)–H functionalization reaction from a simple aryl system to a peptide framework is not a trivial task as a result of the existing multiple chelating sites and ubiquitous C–H bonds.^10^

Recent studies have demonstrated that the installation of an acetyl group within an amino acid of a peptide sequence is particularly useful to produce antibody–drug conjugates through oxime ligation.^11^ Although acetylated proteins are primarily prepared upon enzymatic processes with acetyltransferases or acetyl-CoA derivatives,^12^ the parent processes in short-to-medium peptides remain elusive. The *ortho*-acetylation of simple l-Tyr-OH can occur through a classical Friedel–Crafts reaction with acetyl chloride.^13^ However, the latter cannot be applied within a peptide setting. Partial racemization is often observed (up to 15%), and stoichiometric amounts of AlCl_3_ are required (Scheme 1). In connection with our previous studies on the modification of peptides,^14^ we sought to tackle the synthetic potential of EtOH as a novel acetyl source under oxidative conditions, thereby providing a sustainable yet late-stage acetylation of a number of Tyr-containing compounds. While conceptually innovative, this strategy may suffer from certain drawbacks, such as the lack of selectivity or even an undesired *ortho*-alkoxylation reaction could preferentially occur when using EtOH.^15^ Herein, we present a complementary strategy to perform a chloride-free acetylation of Tyr-containing peptides, which can take place in a late-stage fashion featuring cheap and safe chemical reagents.

**Scheme 1:**
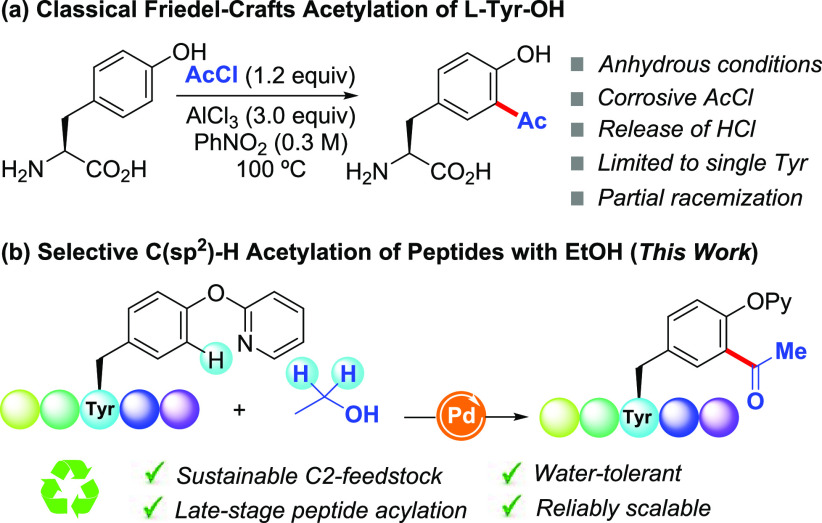
*ortho*-Acetylation of Tyr Derivatives

Inspired by the use of 2-pyridyl ether as an efficient directing group (DG)^16,17^ in the Pd-catalyzed acylation of protected Tyr derivatives with aldehydes recently reported by our group,^10c^ we first selected dipeptide **1a** as the model substrate to test the feasibility of the acetylation reaction with EtOH, thereby harnessing its oxidation toward the sustainable labeling of peptides. After considerable experimentation,^18^ we eventually found that the projected acetylation with EtOH was feasible, and remarkably, neither diacetylation nor *ortho*-alkoxylation upon a C–O bond-forming event was detected.^15^ The optimal conditions involved the use of Pd(OAc)_2_ (10 mol %) and an aqueous solution of inexpensive *tert*-butyl hydroperoxide (TBHP) as the oxidant in toluene as the solvent at 120 °C, which provided compound **2aa** in 60% yield (entry 1 of Table 1). Notably, toluene was not activated to produce the corresponding aroylated product, and EtOH was preferentially oxidized within the reaction conditions.^19^ As expected, control experiments in the absence of either catalyst (entry 2 of Table 1) or oxidant (entry 3 of Table 1) underpinned their critical role in the acetylation process. The performance of the reaction under air resulted in lower yields of compound **2aa**, albeit the process still occurred in a synthetically relevant yield (entry 4 of Table 1). Whereas the yield slightly dropped down to 40% when using 10 equiv of EtOH (entry 5 of Table 1), the process was entirely inhibited in EtOH as the solvent (entry 6 of Table 1). Accordingly, the optimal amount of EtOH was found to be 25 equiv in combination with toluene as the solvent; the use of other related solvents ushered compound **2aa** in lower yields.^18^ Given that multiple oxidation events simultaneously occur, the yield reasonably decreased when lowering the amount of TBHP (entries 7 and 8 of Table 1). However, its use in high excess does not pose a major shortcoming because it is a very cheap oxidant and renders the reaction water-compatible. In fact, an aqueous solution of TBHP afforded better results than other peroxides or persulfates (entries 9 and 10 of Table 1), and Pd(OAc)_2_ clearly outperformed other palladium catalysts^18^ (entries 11 and 12 of Table 1). Finally, we confirmed that subtle modifications on the DG had a determinant impact on the reaction outcome, and the OPy motif was the most active DG toward the target acetylation reaction (Table S3 of the Supporting Information).^16,18^

**Table 1:**
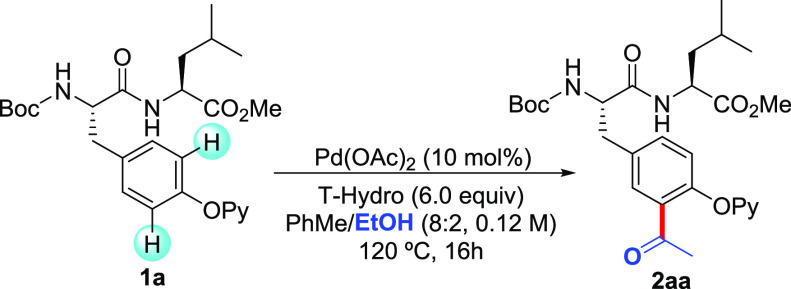
Pd-Catalyzed C–H Acetylation of Compound **1a** with Ethanol^a^

entry	change from standard conditions	**2aa** (%)^b^
1	none	60
2	without Pd(OAc)_2_	0
3	without T-hydro	0
4	under air	53
5	with EtOH (10 equiv)	40
6	with EtOH as the solvent	0
7	T-hydro (5.0 equiv)	59
8	T-hydro (4.0 equiv)	25
9	K_2_S_2_O_8_ instead of T-hydro	0
10	DCP instead of T-hydro	0
11	Pd(OPiv)_2_ instead of Pd(OAc)_2_	44
12	PdCl_2_(MeCN)_2_ instead of Pd(OAc)_2_	46

a Reaction conditions: compound **1a** (0.15 mmol), EtOH (3.75 mmol, 0.2 mL), Pd(OAc)_2_ (10 mol %), and T-hydro (6.0 equiv) in PhMe (1 mL) at 120 °C for 16 h under Ar. T-hydro = *tert*-butyl hydroperoxide solution, 70 wt % in water; DCP = dicumyl peroxide.

b Yield of isolated product after column chromatography.

Although we primarily focused on the unprecedented use of EtOH to acetylate peptides in a site-selective manner, we also evaluated the parent acylation process of dipeptide **1a** using other related aliphatic alcohols. For instance, inexpensive *n*-BuOH, 4-methyl-1-pentanol, and even biologically relevant palmityl alcohol derived from the corresponding fatty acid resulted in the exclusive monoacylated products **2ab**–**2ad** in high yields (Scheme 2).

**Scheme 2:**
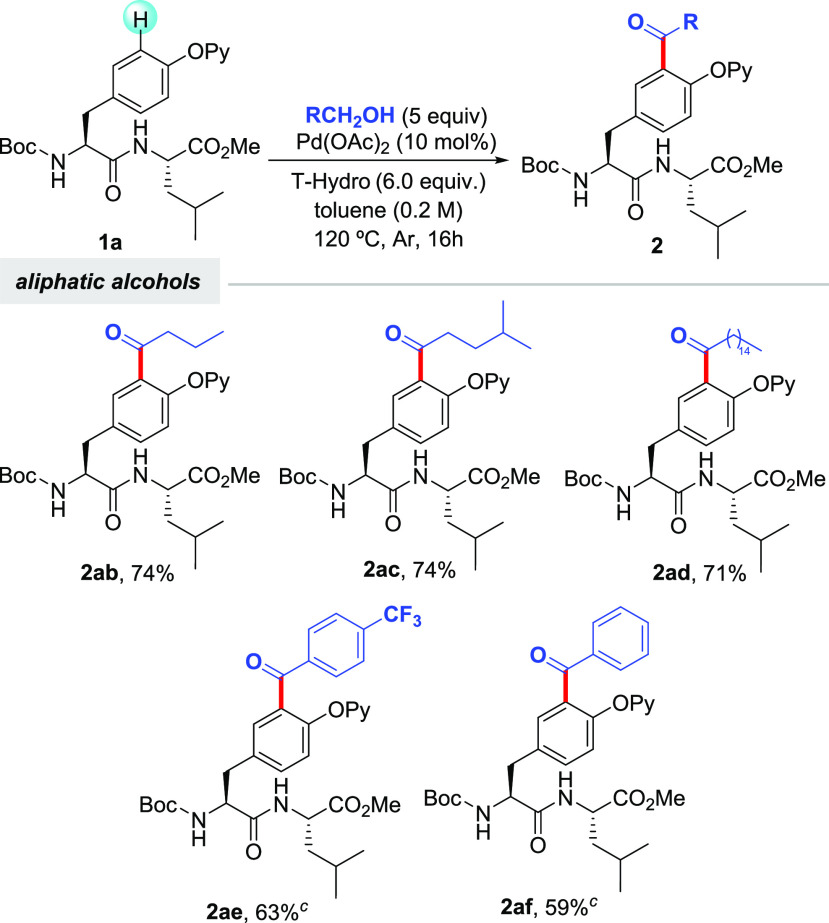
Pd-Catalyzed C–H Acylation of Compound **1a** with Alcohols^,^ Reaction conditions: compound **1a** (0.15 mmol), RCH_2_OH (0.75 mmol), Pd(OAc)_2_ (10 mol %), and T-hydro (6.0 equiv) in PhMe (1 mL) at 120 °C for 16 h under Ar. Yield of isolated product after column chromatography, with the average of at least two independent runs. Reaction conditions: compound **1a** (0.15 mmol), RCH_2_OH (0.45 mmol), Pd(OAc)_2_ (10 mol %), and T-hydro (4.0 equiv) in PhMe (1 mL) at 120 °C for 16 h under Ar.

Noteworthy, in those cases, the amount of alcohol could be significantly reduced to 5.0 equiv. Likewise, activated benzyl alcohols^20,21^ could also be employed to produce the corresponding aroylated dipeptides in 59–63% yields. Owing to their higher tendency toward oxidation, the reaction conditions were slightly modified to avoid the formation of the diaroylated compound; thus, lower amounts of both oxidant and alcohol were required. These experiments revealed that benzyl alcohols could be practical surrogates of benzaldehydes to perform the *ortho*-acylation of Tyr compounds,^10c^ thereby providing exclusively the monofunctionalized products.

We next explored the synthetic scope of the acetylation manifold featuring EtOH in the challenging setting of short-to-medium-size peptides (Scheme 3). Notably, peptides bearing Val (**1c**), Phe (**1d**), Lys (**1e**), Ala (**1f**), Pro (**1g**), Gly (**1h**), Ser (**1i**), Asp (**1j**), Glu (**1k**), Ile (**1l**), Tyr (**1m**), and even Arg (**1p**) were found compatible with the reaction conditions and provided the corresponding acetylated peptides in moderate to good yields. Note that the N terminus and other oxidizable amino acid residues housing a free amino, an alcohol, a carboxylic acid, or a guanidine motif (Lys, Ser, Asp, Glu, and Arg, respectively) were equipped with protecting groups to achieve chemoselectivity. Notably, this labeling technique was applicable to Tyr residues located at the N and C terminals as well as inner positions. Importantly, tetrapeptide **1n** and hexapeptide **1o** having the sequence of biologically relevant endomorphin-2 and neuromedin N, respectively, were also acetylated with EtOH, hence showcasing the high utility of this method toward the site-selective tagging of complex biomolecules. As previously anticipated, other alcohols could also be selectively installed at the *ortho* position of the Tyr unit within di-, tri-, and tetrapeptide derivatives (**2db**, **2kb**, and **2q** and **2r**). In general, the reactions were very clean, and side products were not observed, albeit full conversion was not always achieved and sometimes PhMe was replaced by more oxidizing PhCl. Besides, unlike classical Friedel–Crafts acetylation, our method features the use of EtOH as a sustainable C_2_ source to accomplish a synthetically meaningful transformation, wherein a high number of C–H bonds are activated. In this respect, the acylation of compound **1a** could be performed in gram scale when using EtOH and BuOH with a remarkable 62 and 74% yield, respectively (Scheme 4). In these cases, the amount of EtOH and oxidant could be slightly reduced without affecting the reaction outcome, which represents a promising starting point for applied research.

**Scheme 3:**
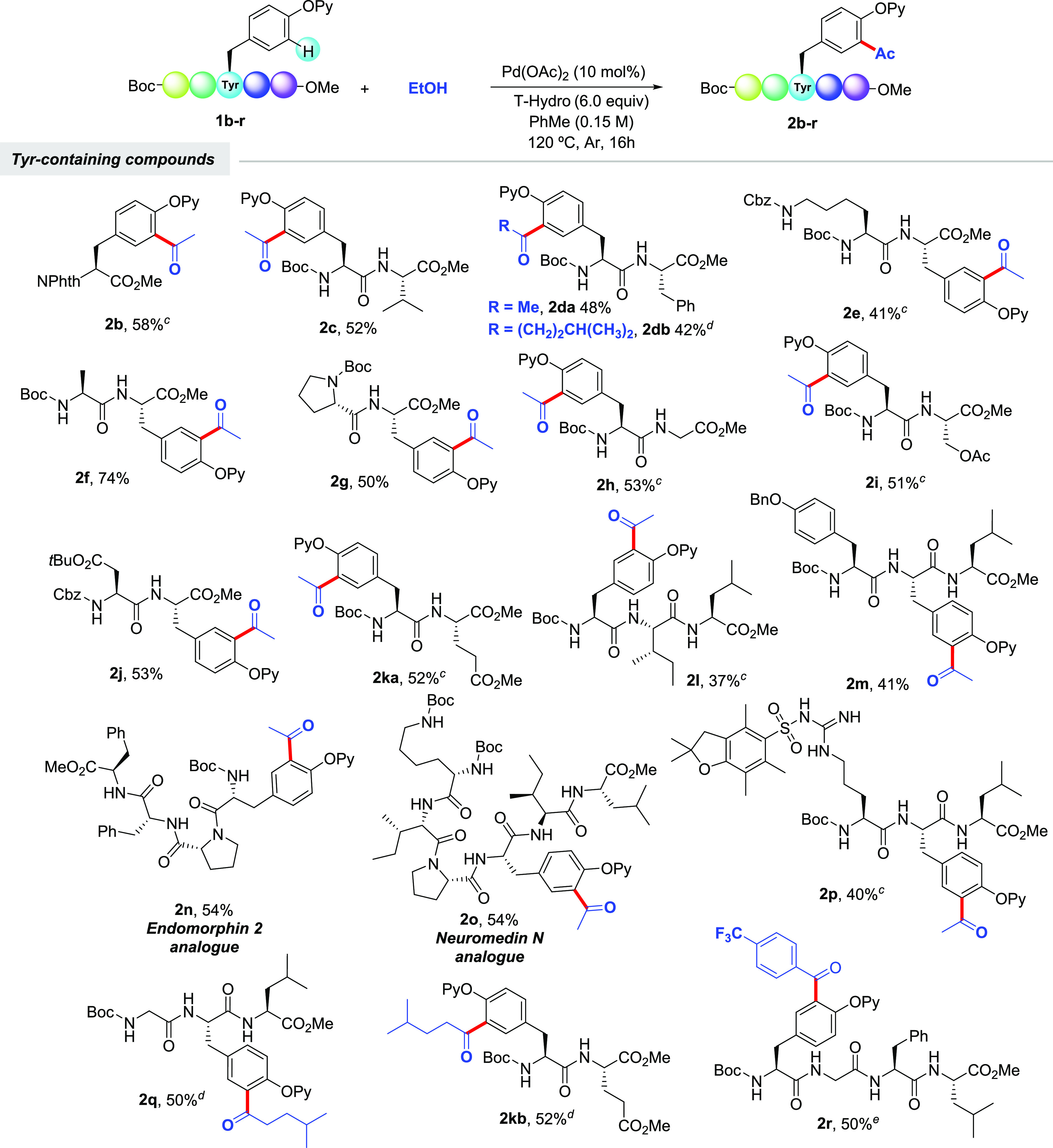
Pd-Catalyzed C–H Acylation of Tyr-Containing Oligopeptides with EtOH and Other Alcohols^,^ The same as for entry 1 of Table 1. Yield of isolated product after column chromatography, with the average of at least two independent runs with a variable yield by no more than 5% between runs. Using PhCl instead of PhMe as the solvent. Compound **1** (0.15 mmol), alcohol (0.45 mmol), Pd(OAc)_2_ (10 mol %), and T-hydro (4.0 equiv) in PhMe (1 mL) at 120 °C for 16 h under Ar. Compound **1** (0.15 mmol), alcohol (0.75 mmol) Pd(OAc)_2_ (10 mol %), and T-hydro (6.0 equiv) in PhMe (1 mL) at 120 °C for 16 h under Ar.

**Scheme 4:**
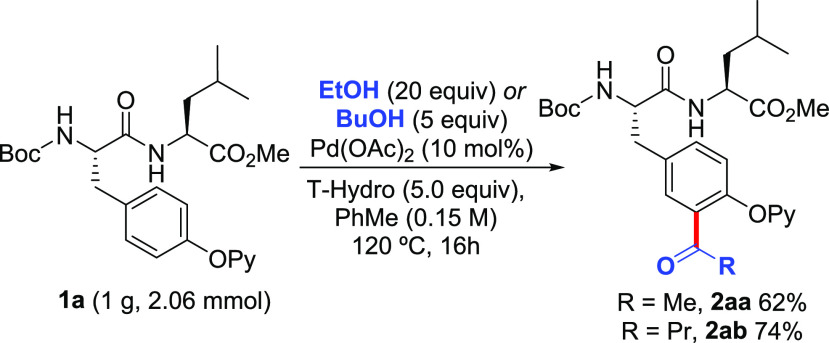
Gram-Scale Synthesis

To gain some insights into the reaction mechanism, we conducted some control experiments. We found that the acetylation of compound **1a** was suppressed in the presence of 2,2,6,6-tetramethylpiperidine 1-oxyl (TEMPO), which indicated that a radical pathway may be operative.^18^ Furthermore, assuming that EtOH could be oxidized to acetaldehyde within the course of the reaction, we performed some tests with MeCHO as the coupling partner.^18^ When using acetaldehyde under the acylation conditions previously developed by our group involving water as the solvent,^10c^ traces of compound **2aa** were obtained, which reveals the subtleties of installing a simple acetyl group. Notably, the use of PhMe as the solvent resulted in mixtures of mono- and diacetylated products, and the use of a high excess of MeCHO ushered in the exclusive formation of diacetylated compound **2aa′** in 62% yield (Table S4 of the Supporting Information).^18^ Accordingly, if EtOH is *in situ* transformed into MeCHO in the presence of TBHP,^22^ the reaction mechanism should be akin to those of related acylations with aldehydes described in the literature.^18,21^ The high selectivity toward the monoacetylation could be due to the lower reactivity of EtOH in comparison to the corresponding aldehyde.

In summary, we have demonstrated the high versatility of EtOH as a sustainable feedstock to tag Tyr-containing peptides in a late-stage fashion. This reliably scalable platform represents an innovative avenue for the diversification of Tyr-containing compounds. Salient features of this method are the widespread availability and low cost of EtOH and other related alcohols, the compatibility with an aqueous environment, and the site-selectivity toward the monofunctionalization of the Tyr unit within a peptide setting. Accordingly, this Pd-catalyzed acetylation manifold represents a useful tool for the facile modification of a virtually unlimited number of biologically relevant peptides.

## Supporting Information Available

Experimental procedures, syntheses and characterization of all new compounds, and tables with details of several optimization studies (PDF)

## References

[ref1] Anastas P.; Eghbali N. Green Chemistry: Principles and Practice. Chem. Soc. Rev. 2010, 39, 301 10.1039/B918763B. 20023854

[ref2] aSchaub T. Efficient Industrial Organic Synthesis and the Principles of Green Chemistry. Chem. - Eur. J. 2021, 27, 1865 10.1002/chem.202003544. 33448523

[ref3] aKühlborn J.; Groß J.; Opatz T. Making Natural Products from Renewable Feedstocks: Back to the Roots?. Nat. Prod. Rep. 2020, 37, 380 10.1039/C9NP00040B. 31625546

[ref4] aMeyer C. C.; Stafford N. P.; Cheng M. J.; Krische M. J. Ethanol: Unlocking an Abundant Renewable C2-Feedstock for Catalytic Enantioselective C–C Coupling. Angew. Chem., Int. Ed. 2021, 60, 10542 10.1002/anie.202102694. PMC8085048 33689214

[ref5] aLenci E.; Trabocchi A. Peptidomimetic Toolbox for Drug Discovery. Chem. Soc. Rev. 2020, 49, 3262 10.1039/D0CS00102C. 32255135

[ref6] San Segundo M.; Correa A. Cross-Dehydrogenative Coupling Reactions for the Functionalization of α-Amino Acid Derivatives and Peptides. Synthesis 2018, 50, 2853 10.1055/s-0037-1610073.

[ref7] aKing T. A.; Kandemir J. M.; Walsh S. J.; Spring D. R. Photocatalytic Methods for Amino Acid Modification. Chem. Soc. Rev. 2021, 50, 39 10.1039/D0CS00344A. 33174541

[ref8] aGuillemard L.; Kaplaneris N.; Ackermann L.; Johansson M. J. Late-Stage C–H Functionalization Offers New Opportunities in Drug Discovery. Nat. Rev. Chem. 2021, 5, 522 10.1038/s41570-021-00300-6. 37117588

[ref9] aCorrea A. Metal-Catalyzed C(sp^2^)–H Functionalization Processes of Phenylalanine- and Tyrosine-Containing Peptides. Eur. J. Inorg. Chem. 2021, 2021, 2928 10.1002/ejic.202100374.

[ref10] aLong T.; Liu L.; Tao Y.; Zhang W.; Quan J.; Zheng J.; Hegemann J. D.; Uesugi M.; Yao W.; Tian H.; Wang H. Light-Controlled Tyrosine Nitration of Proteins. Angew. Chem., Int. Ed. 2021, 60, 13414 10.1002/anie.202102287. 33847040

[ref11] aChudasama V.; Maruani A.; Caddick S. Recent Advances in the Construction of Antibody–Drug Conjugates. Nat. Chem. 2016, 8, 114 10.1038/nchem.2415. 26791893

[ref12] Yang Y.-Y.; Ascano J. M.; Hang H. C. Bioorthogonal Chemical Reporters for Monitoring Protein Acetylation. J. Am. Chem. Soc. 2010, 132, 3640 10.1021/ja908871t. 20192265 PMC2848987

[ref13] aSchneider T.; Martin J.; Durkin P. M.; Kubyshkin V.; Budisa N. The Regioselective Synthesis of o-Nitrobenzyl DOPA Derivatives. Synthesis 2017, 49, 2691 10.1055/s-0036-1588766.

[ref14] aAndrade-Sampedro P.; Matxain J. M.; Correa A. Pd-Catalyzed C(sp^2^)–H Alkoxycarbonylation of Phenethyl- and Benzylamines with Chloroformates as CO Surrogates. Chem. - Eur. J. 2021, 27, 5782 10.1002/chem.202005338. 33433940

[ref15] aEnthaler S.; Company A. Palladium-Catalysed Hydroxylation and Alkoxylation. Chem. Soc. Rev. 2011, 40, 4912 10.1039/c1cs15085e. 21643619

[ref16] aDutta U.; Maiti S.; Bhattacharya T.; Maiti D. Arene diversification through distal C(sp^2^)–H functionalization. Science 2021, 372, eabd5992 10.1126/science.abd5992. 33986152

[ref17] aLou S.-J.; Chen Q.; Wang Y.-F.; Xu D.-Q.; Du X.-H.; He J.-Q.; Mao Y.-J.; Xu Z.-Y. Selective C–H Bond Fluorination of Phenols with a Removable Directing Group: Late-Stage Fluorination of 2-Phenoxyl Nicotinate Derivatives. ACS Catal. 2015, 5, 2846 10.1021/acscatal.5b00306.

[ref18] For more details, see the Supporting Information.

[ref19] aGuin S.; Rout S. K.; Banerjee A.; Nandi S.; Patel B. K. Four Tandem C–H Activations: A Sequential C–C and C–O Bond Making via a Pd-Catalyzed Cross Dehydrogenative Coupling (CDC) Approach. Org. Lett. 2012, 14, 5294 10.1021/ol302438z. 23020217

[ref20] aPark J.; Kim A.; Sharma S.; Kim M.; Park E.; Jeon Y.; Lee Y.; Kwak J. H.; Jung Y. H.; Kim I. S. Direct Acylation of N-Benzyltriflamides from the Alcohol Oxidation Level via Palladium-Catalyzed C–H Bond Activation. Org. Biomol. Chem. 2013, 11, 2766 10.1039/c3ob40140e. 23493982

[ref21] aKumar P.; Dutta S.; Kumar S.; Bahadur V.; Van der Eycken E. V.; Vimaleswaran K. S.; Parmar V. S.; Singh B. K. Aldehydes: Magnificent Acyl Equivalents for Direct Acylation. Org. Biomol. Chem. 2020, 18, 7987 10.1039/D0OB01458C. 33000845

[ref22] aDobereiner G. E.; Crabtree R. H. Dehydrogenation as a Substrate-Activating Strategy in Homogeneous Transition-Metal Catalysis. Chem. Rev. 2010, 110, 681 10.1021/cr900202j. 19938813

