# Supplementary material for: Late-Stage C–H Acylation of Tyrosine-Containing Oligopeptides with Alcohols
Source: Org Lett. 2021 Sep 3;23(18):7279–84. doi: 10.1021/acs.orglett.1c02764 (PMC8453636; doi:10.1021/acs.orglett.1c02764)

Supporting Information

Late-Stage C–H Acylation of Tyrosine-Containing Oligopeptides with Alcohols

*Iñaki Urruzuno,[a] Paula Andrade-Sampedro[a,b] and Arkaitz Correa[a]**

[a] University of the Basque Country (UPV/EHU), Department of Organic Chemistry I, Joxe Mari Korta R&D Center, Avda. Tolosa 72, 20018 Donostia-San Sebastián (Spain).

E-mail: arkaitz.correa@ehu.eus

[b] Donostia International Physics Center (DIPC), Paseo Manuel de Lardizabal 4, 20018 Donostia-San Sebastián (Spain)

**1.-General Considerations**	**S2**
**2.-Optimization Details**	**S3**
**3.-Preparation of the Starting Materials**	**S6**
**4.-Pd-Catalyzed C(sp^2)-H Acylation of Tyr-Containing Compounds**	**S13**
**5.-Control Experiments and Mechanism Proposal**	**S30**
**6.-^{1}H NMR and ^{13}C NMR Spectra**	**S32**

1.-General Considerations

Reagents. Commercially available materials were used without further purification. Palladium acetate and T-Hydro (*tert*-butyl hydroperoxide solution, 70 wt % in water) were purchased from Sigma-Aldrich. Ethanol absolute was purchased from VWR. All the alcohols were commercially available and were used without further purification.

Analytical Methods. ^{1}H NMR and ^{13}C NMR spectra as well as IR, HRMS and melting points (where applicable) are included for all new compounds. ^{1}H NMR and ^{13}C NMR spectra were recorded on a Bruker 300, 400 or 500 MHz at 20 ^{o}C, unless otherwise indicated. All ^{1}H NMR spectra are reported in parts per million (ppm) downfield of TMS and were measured relative to the signals for $CHCl_3$ (7.26 ppm), unless otherwise indicated. All ^{13}C NMR spectra were reported in ppm relative to residual $CHCl_3$ (77 ppm), unless otherwise indicated, and were obtained with ^{1}H decoupling. Coupling constants, *J*, are reported in Hertz. Melting points were measured using open glass capillaries in a Büchi SMP-20 apparatus. High resolution mass spectra (HRMS) were performed by SGIker and were acquired on a LC/Q-TOF mass spectrometer equipped with an electrospray source ESI Agilent Jet Stream. Infrared spectra were recorded on a Bruker Alpha P. Flash chromatography was performed with EM Science silica gel 60 (230-400 mesh). The yields reported in the manuscript correspond to isolated yields and represent an average of at least two independent runs.

2.-Optimization Details

General Procedure:

A reaction tube containing a stirring bar was charged with Boc-Tyr(OPy)-Leu-OMe[1] (**1a**) (0.15 mmol, 73 mg), oxidant (0.90 mmol) (if solid) and metal source (10 mol %). The reaction tube was then evacuated and back-filled with dry argon (this sequence was repeated up to three times). Then, EtOH (3.75 mmol, 220 μL), oxidant (if liquid), and the corresponding solvent (1.0 mL) were added by syringe under argon atmosphere. The reaction tube was next warmed up to the corresponding temperature in a heating block and stirred for 16 hours. The mixture was allowed to cool to room temperature, diluted with EtOAc and washed with a saturated aqueous solution of $NaHCO_3$. The aqueous layer was extracted with EtOAc, and the combined organic layers were dried over $MgSO_4$ and concentrated under reduced pressure. The resulting crude was purified by flash chromatography (hexanes/EtOAc, 1/1). The purity of the corresponding product **2aa** was verified by ^{1}H NMR.

[1] San Segundo, M.; Correa, A. *Chem. Sci.* **2020**, *11*, 11531.

Table S1. Screening of Acylation with EtOH[a]

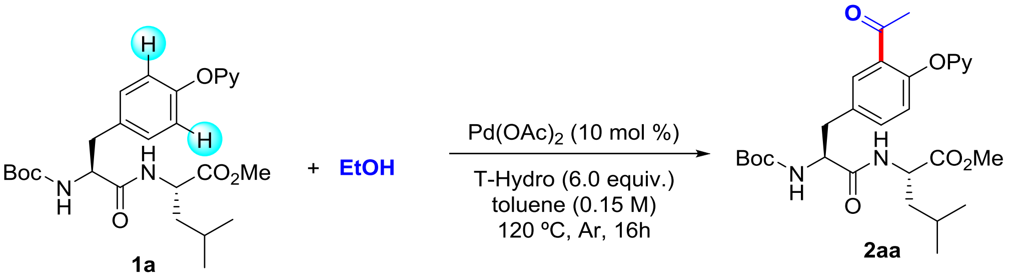

Entry	Variation from the standard conditions	2aa (%)[b]
1	none	60
2	DTBP as oxidant	0
3	TBPB as oxidant	0
4	DCP as oxidant	0
5	PIDA as oxidant	degradation
6	$K_2S_2O_8$ as oxidant	degradation
7	1,4-dioxane as solvent	15
8	toluene/water (9:1)	21
9	DMF as solvent	0
10	DCE as solvent	degradation
11	EtOH as solvent	degradation
12	*t*BuPh as solvent	48
13	o-xylene as solvent	48
14	$PhCF_3$ as solvent	45
15	$PdCl_2$ as catalyst	27
16	$Pd(OPiv)_2$ as catalyst	44
17	$Pd(PPh_3)_4$ as catalyst	41
18	$PdCl_2(MeCN)_2$ as catalyst	46
19	$Pd(dba)_2$ as catalyst	42
20	$Pd(OTFA)_2$ as catalyst	35
21	under air	53
22	T = 130 ºC	54
23	T-Hydro (5.0 equiv)	60
24	T-Hydro (4.0 equiv)	25
25	EtOH (10 equiv)	40
26	$Pd(OAc)_2$ (5 mol%)	32

[a]Reaction conditions: **1a** (0.15 mmol), EtOH (3.75 mmol), TBHPaq (0.90 mmol), toluene (1.0 mL), Ar, 16h at 120 °C. [b] Yield of isolated product after column chromatography.

Table S2. Screening with 4-(trifluoromethyl)benzyl alcohol[a]

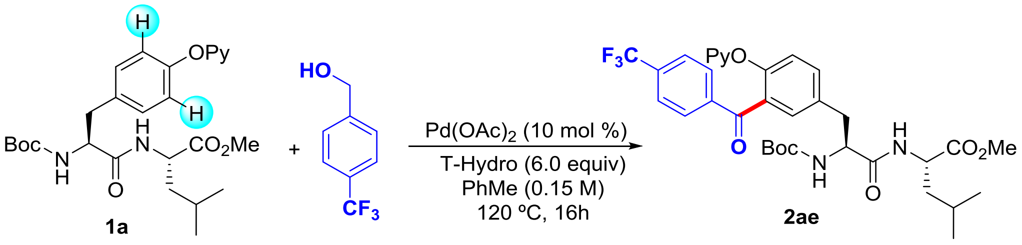

Entry	Variation from the standard conditions	Yield (%)[b]
1	none	40 (20)[c]
2	DCE as solvent	48[c]
3	PhCl as solvent	43[c]
4	$Pd(OTFA)_2$ as catalyst	degradation
5	T-Hydro (4.0 equiv)	63
6	H_2O as solvent	30[c]

[a]Reaction conditions: **1a** (0.15 mmol), alcohol (0.45 mmol), $Pd(OAc)_2$ (10 mol%), T-Hydro (6.0 equiv), toluene (1.0 mL), Ar, 16h at 120 °C. [b] Yield of isolated product after column chromatography. [c] Yield of isolated difunctionalized product after column chromatography.

Table S3. Influence of the DG[a]

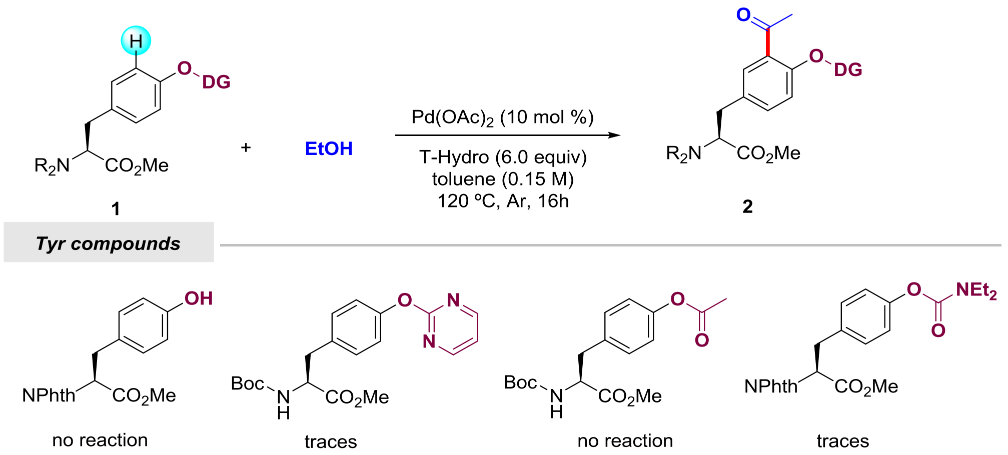

[a]Reaction conditions: **1** (0.15 mmol), EtOH (3.75 mmol), T-Hydro (6.0 equiv), toluene (1.0 mL), Ar, 16h at 120 °C.

3.-Preparation of the Starting Materials

Tyr derivatives

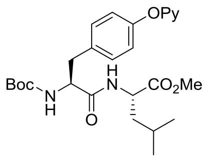

Boc-Tyr(OPy)-Leu-OMe
1a[a]

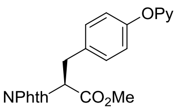

NPhth-Tyr(OPy)-OMe
1b[b]

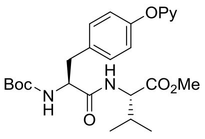

Boc-Tyr(OPy)-Val-OMe
1c[a]

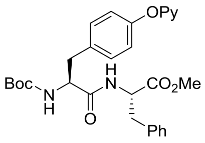

Boc-Tyr(OPy)-Phe-OMe
1d[a]

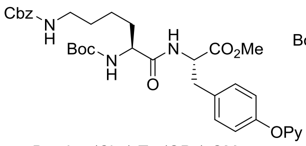

Boc-Lys(Cbz)-Tyr(OPy)-OMe
1e[a]

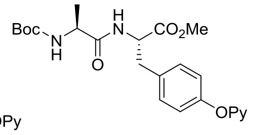

Boc-Ala-Tyr(OPy)-OMe
1f[a]

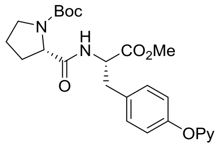

Boc-Pro-Tyr(OPy)-OMe
1g[a]

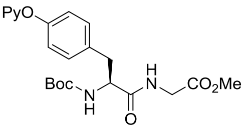

Boc-Tyr(OPy)-Gly-OMe
1h[a]

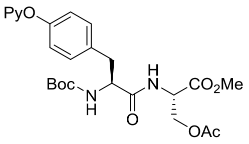

Boc-Tyr(OPy)-Ser(OAc)-OMe
1i[b]

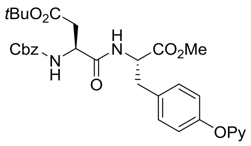

Cbz-Asp(O*t*Bu)-Tyr(OPy)-OMe
1j[b]

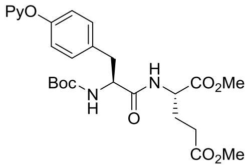

Boc-Tyr(OPy)-Glu(OMe)-OMe
1k[a]

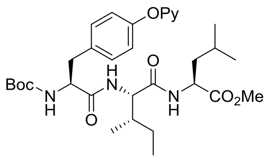

Boc-Tyr(OPy)-Ile-Leu-OMe
1l[a]

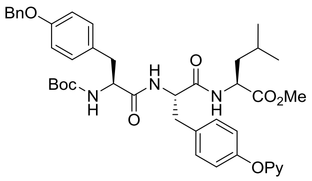

Boc-Tyr(OBn)-Tyr(OPy)-Leu-OMe
1m[b]

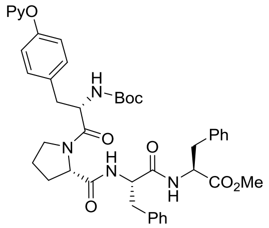

Boc-Tyr(OPy)-Pro-Phe-Phe-OMe
1n[a]

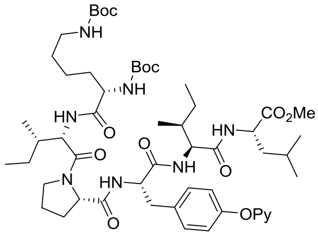

Boc-Lys(Boc)-Ile-Pro-Tyr(OPy)-Ile-Leu-OMe
1o[a]

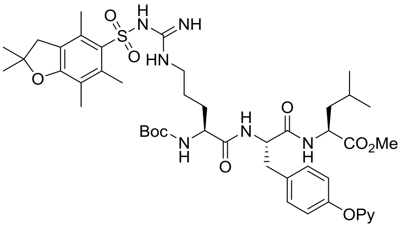

Boc-Arg(Pbf)-Tyr(OPy)-Leu-OMe
1p[b]

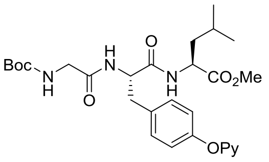

Boc-Gly-Tyr(OPy)-Leu-OMe
1q[b]

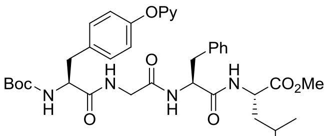

Boc-Tyr(OPy)-Gly-Phe-Leu-OMe
1r[b]

[a] Prepared following literature procedures. [b] Synthesis reported herein.

General Procedure for the O-Arylation of Tyr-Containing Peptides[2]

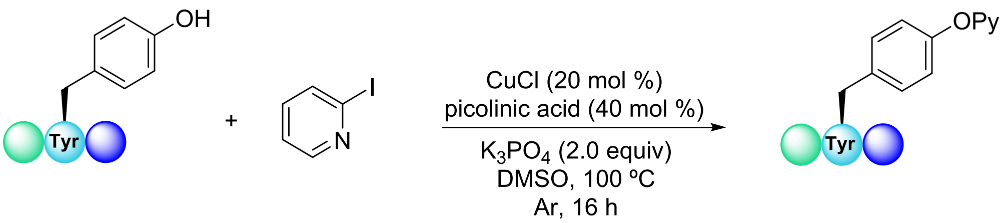

A reaction tube containing a stirring bar was charged with the corresponding tyrosine derivative (1.0 equiv), CuCl (20 mol %), K_3PO_4 (2.0 equiv) and 2-picolinic acid (40 mol %). The reaction tube was then evacuated and back-filled with dry Ar (this sequence was repeated up to three times). Then DMSO (2.5 mL/mmol) and 2-iodopyridine (2.0 equiv) were added under argon atmosphere. The reaction tube was next warmed up to 100 °C and stirred for 16 h. After cooling down to room temperature, brine was added to the above solution, washed with a saturated aqueous solution of $NaHCO_3$, and extracted with EtOAc. The organic layers were combined and evaporated under vacuum. The resulting crude was then purified by column chromatography to afford the corresponding product.

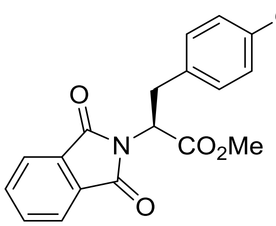

(1b)

Methyl (*S*)-2-(1,3-dioxoisoindolin-2-yl)-3-(4-(pyridin-2-yloxy)phenyl)propanoate (1b). Following the general procedure, using NPhth-Tyr-OMe[3] (12.31 mmol, 4.00 g) provided 4.30 g (87% yield) of **1b** as a white solid. Mp 89-90 °C. Column chromatography (Hex/EtOAc 1:1). ^{1}H NMR (400 MHz, $CDCl_3$) δ 8.14 (ddd, *J* = 5.0, 2.0, 0.8 Hz, 1H), 7.79 (dd, *J* = 5.4, 3.1 Hz, 2H), 7.69 (dd, *J* = 5.5, 3.1 Hz, 2H), 7.62 (ddd, *J* = 8.3, 7.2, 2.0 Hz, 1H), 7.23 – 7.13 (m, 2H), 7.04 – 6.89 (m, 3H), 6.84 – 6.70 (m, 1H), 5.17 (dd, *J* = 10.8, 5.7 Hz, 1H), 3.78 (s, 3H), 3.73 – 3.44 (m, 2H). ^{13}C NMR (101 MHz, $CDCl_3$) δ 169.4, 167.6, 163.7, 153.0, 147.8, 139.5, 134.3, 133.0, 131.7, 130.2, 123.6, 121.3, 118.6, 111.5, 53.2, 53.0, 34.2. IR (cm^{-1}): 1753, 1707, 1388, 716. HRMS (ESI) m/z: (M^+) *calcd* for ($C_{23}H_{18}N_2O_5$): 402.1216, *found* 402.1214.

---

[2] Chu, J.-H.; Chen, S.-T.; Chiang, M.-F.; Wu, M.-J. *Organometallics* **2015**, *34*, 953.
[3] Sather, A. C.; Lee, H. G.; De La Rosa, V. Y.; Yang, Y.; Müller, P.; Buchwald, S. L. *J. Am. Chem. Soc.* **2015**, *137*, 13433.

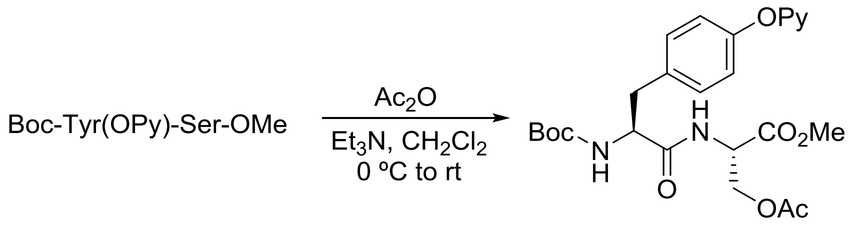

(1i)

Methyl *O*-acetyl-*N*-((*S*)-2-((*tert*-butoxycarbonyl)amino)-3-(4-(pyridin-2-yloxy) phenyl)propanoyl)-*L*-serinate (1i). To a solution of Boc-Tyr(OPy)-Ser-OMe[1] (2.0 mmol, 919 mg) and triethylamine (6.0 mmol, 0.83 mL) in dichloromethane (10 mL), acetic anhydride (4.0 mmol, 0.39 mL) was added at 0 ºC and the resulting solution was stirred at room temperature for 5 h. The organic phase was washed with a solution of NaOH 1M and brine, consecutively. The solvent was removed under reduced pressure and the product was purified by flash chromatography (EtOAc/hexanes, 6:4) to provide 519 mg (52% yield) of **1i** as a white solid. Mp 91-92 ºC. ^{1}H NMR (300 MHz, $CDCl_3$) δ 8.18 (s, 1H), 7.68 (t, *J* = 7.7 Hz, 1H), 7.24 (d, *J* = 10.7 Hz, 2H), 7.07 (d, *J* = 8.3 Hz, 2H), 7.04 – 6.94 (m, 1H), 6.89 (d, *J* = 8.3 Hz, 1H), 6.72 (d, *J* = 7.3 Hz, 1H), 5.08 – 5.01 (m, 1H), 4.83 – 4.77 (m, 1H), 4.49 – 4.15 (m, 3H), 3.75 (s, 3H), 3.09 (dd, *J* = 6.6, 3.9 Hz, 2H), 2.02 (s, 3H), 1.43 (s, 9H). ^{13}C NMR (75 MHz, $CDCl_3$) δ 171.2, 170.5, 169.4, 163.6, 153.2, 147.7, 139.4, 132.6, 130.6, 130.5, 121.4, 118.5, 111.6, 80.4, 63.7, 55.6, 52.8, 51.8, 37.5, 28.3, 20.6. IR (cm^{-1}): 3292, 1645, 1505, 1427, 1242, 1152. HRMS (ESI) m/z: (M^+) *calcd* for ($C_{25}H_{31}N_3O_8$): 501.2111, *found* 501.2118.

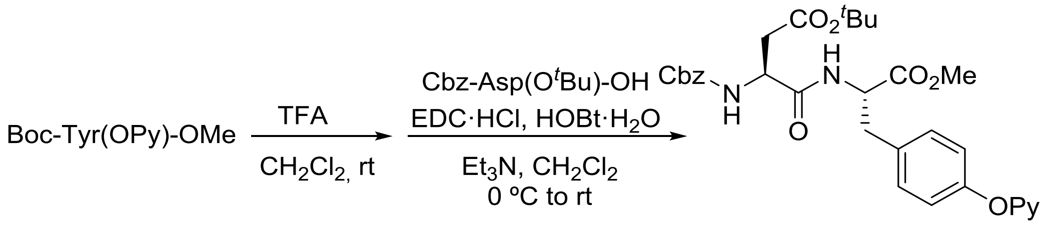

(1j)

***tert*-Butyl (*S*)-3-[((benzyloxy)carbonyl)amino]-4-[((*S*)-1-methoxy-1-oxo-3-(4-(pyridin-2-yloxy)phenyl)propan-2-yl)amino]-4-oxobutanoate (1j).** A solution of Boc-Tyr(OPy)-OMe[1] (2.0 mmol, 745 mg) in dichloromethane was treated with trifluoroacetic acid (20 mmol, 1.5 mL) and stirred for 5 h. After evaporation of the solvent, the resulting crude was diluted with EtOAc and washed with a saturated aqueous solution of $NaHCO_3$. After evaporation of the solvent, the so-obtained crude (without further purification) was dissolved in dichloromethane (10 mL) at 0 ºC. EDC·HCl (2.4 mmol, 460 mg), HOBt (2.4 mmol, 323 mg), Cbz-Asp(O^{t}Bu)-OH (2.4 mmol, 647 mg) and triethylamine (3.0 mmol, 0.45 mL) were subsequently added and stirred at room temperature overnight. The resulting solution was washed with water and extracted with dichloromethane. The

solvent was removed under reduced pressure and the corresponding product was purified by flash chromatography (Hexane:EtOAc, 6:4) to provide 890 mg (77% yield) of **1j** as a colorless oil. ^{1}H NMR (300 MHz, $CDCl_3$) δ 8.19 – 8.05 (m, 1H), 7.63 (t, *J* = 7.7 Hz, 1H), 7.32 – 7.23 (m, 4H), 7.15 – 7.08 (m, 3H), 6.99 (d, *J* = 8.3 Hz, 2H), 6.96 – 6.91 (m, 1H), 6.84 (d, *J* = 8.3 Hz, 1H), 6.09 (d, *J* = 8.2 Hz, 1H), 5.18 – 4.97 (m, 2H), 4.85 – 4.77 (m, 1H), 4.57 – 4.52 (s, 1H), 3.67 (s, 3H), 3.13 – 3.05 (m, 1H), 3.05 – 2.95 (m, 1H), 2.85 – 2.71 (m, 1H), 2.67 – 2.53 (m, 1H), 1.38 (s, 9H). ^{13}C NMR (75 MHz, $CDCl_3$) δ 171.3, 170.7 170.2, 163.5, 155.9, 153.0, 147.4, 139.3, 135.9, 131.9, 130.4, 128.3, 128.0, 127.9, 121.1, 118.3, 111.3, 81.5, 67.0, 53.2, 52.2, 50.9, 37.0, 27.8. IR (cm^{-1}): 3289, 2956, 1647, 1241, 1163. HRMS (ESI) m/z: (M$^+$) *calcd* for ($C_{31}H_{35}N_3O_8$): 577.2424, *found* 577.2427.

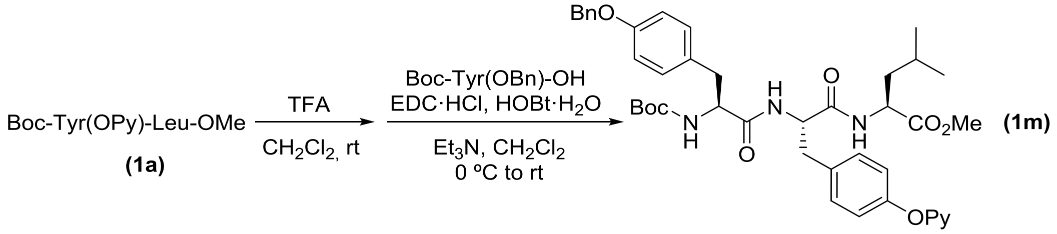

Methyl [(*S*)-2-((*S*)-3-(4-(benzyloxy)phenyl)-2-((*tert*-butoxycarbonyl)amino) propanamido-3-4-(pyridin-2-yloxy)phenyl)propanoyl]-*L*-leucinate (1m). A solution of Boc-Tyr(OPy)-Leu-OMe (2.06 mmol, 1.00 g) in dichloromethane was treated with trifluoroacetic acid (20.6 mmol, 1.54 mL) and stirred for 5 h. After evaporation of the solvent, the resulting crude was diluted with EtOAc and washed with a saturated aqueous solution of $NaHCO_3$. After evaporation of the solvent, the so-obtained crude (without further purification) was dissolved in dichloromethane (10 mL) at 0 ºC. EDC·HCl (2.25 mmol, 431mg), HOBt (2.25 mmol, 304 mg), Boc-Tyr(OBn)-OH (2.25 mmol, 837 mg) and triethylamine (2.25 mmol, 0.30 mL) were subsequently added and stirred at room temperature overnight. The resulting solution was washed with water and extracted with dichloromethane. The solvent was removed under reduced pressure and the corresponding product was purified by flash chromatography (Hexane:EtOAc, 1:1) to provide 810 mg (54% yield) of **1m** as a white solid. Mp 176-177 °C. ^{1}H NMR (300 MHz, $CDCl_3$) δ 8.12 (ddd, *J* = 5.0, 2.0, 0.9 Hz, 1H), 7.65 (ddd, *J* = 8.2, 7.1, 2.0 Hz, 1H), 7.47 – 7.27 (m, 4H), 7.20 – 6.81 (m, 11H), 6.63 (d, *J* = 8.0 Hz, 1H), 6.49 – 6.37 (m, 1H), 5.02 (s, 2H), 4.98 – 4.84 (m, 1H), 4.67 (q, *J* = 7.0 Hz, 1H), 4.56 – 4.58 (m, 1H), 4.38 – 4.17 (m, 1H), 3.70 (s, 3H), 3.04 (ddt, *J* = 34.4, 14.2, 6.5 Hz, 4H), 1.65 – 1.42 (m, 3H), 1.36 (s, 9H), 0.97 – 0.78 (m, 6H). ^{13}C NMR (75 MHz, $CDCl_3$) δ 172.8, 171.4, 170.3, 163.8, 158.0, 153.2, 147.7, 139.6, 137.0, 132.7, 130.8, 130.6, 130.4, 128.7, 128.6, 128.6, 127.5, 121.6,

121.5, 118.6, 115.3, 115.2, 114.9, 111.6, 80.6, 70.1, 56.0, 54.2, 52.4, 51.1, 41.3, 37.2, 28.3, 24.8, 22.8, 22.0. IR (cm^{-1}): 3316, 1741, 1687, 1643, 1247. HRMS (ESI) m/z: (M^+) *calcd* for ($C_{42}H_{50}N_4O_8$): 738.3629, *found* 738.3639.

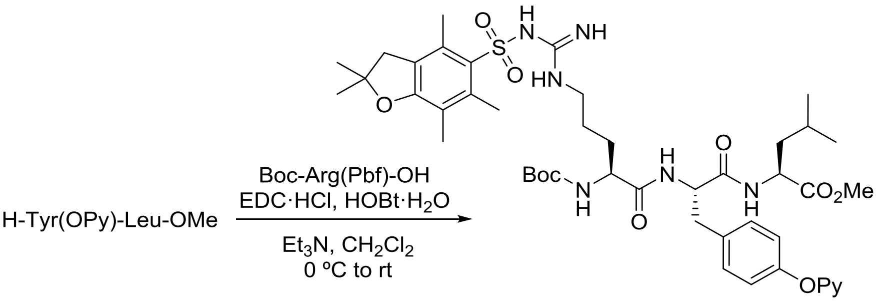

(1p)

Methyl [(*S*)-2-((*S*)-2-((*tert*-butoxycarbonyl)amino)-5-(3-((2,2,4,6,7-pentamethyl-2,3-dihydrobenzofuran-5-yl)sulfonyl)guanidino)pentanamido)-3-(4-(pyridin-2-yloxy) phenyl)propanoyl]-*L*-leucinate (1p). A solution of H-Tyr(OPy)-Leu-OMe (2.02 mmol, 777 mg) was dissolved in dichloromethane (10 mL) at 0 ºC. EDC·HCl (2.22 mmol, 425mg), HOBt (2.22 mmol, 300 mg), Boc-Arg(Pbf)-OH (2.22 mmol, 1.16 g) and triethylamine (2.22 mmol, 0.30 mL) were subsequently added and stirred at room temperature overnight. The resulting solution was washed with water and extracted with dichloromethane. The solvent was removed under reduced pressure and the corresponding product was purified by flash chromatography (EtOAc) to provide 890 mg (47% yield) of **1p** as a white solid. Mp 122-123 °C. ^{1}H NMR (300 MHz, $CDCl_3$) δ 8.25 – 7.99 (m, 1H), 7.73 – 7.45 (m, 2H), 7.20 – 7.07 (m, 3H), 7.04 – 6.90 (m, 3H), 6.81 (dd, *J* = 8.4, 0.9 Hz, 1H), 6.40 (s, 2H), 6.25 (s, 1H), 5.65 (d, *J* = 7.4 Hz, 1H), 4.68 (q, *J* = 7.4 Hz, 1H), 4.46 (q, *J* = 7.2 Hz, 1H), 4.19 (d, *J* = 6.6 Hz, 1H), 3.62 (s, 3H), 3.08 (ddd, *J* = 17.1, 11.1, 6.3 Hz, 4H), 2.89 (s, 2H), 2.56 (s, 3H), 2.49 (s, 3H), 2.05 (s, 3H), 1.86 – 1.46 (m, 7H), 1.41 (s, 6H), 1.43 (s, 9H), 0.94 – 0.66 (m, 6H). ^{13}C NMR (75 MHz, $CDCl_3$) δ 172.9, 172.6, 171.2, 163.7, 158.7, 156.6, 155.9, 153.0, 147.6, 139.6, 138.3, 133.1, 132.9, 132.2, 130.7, 124.6, 121.1, 118.6, 117.5, 111.5, 86.4, 79.9, 54.8, 54.0, 52.2, 51.0, 43.2, 40.8, 40.3, 37.0, 29.7, 28.6, 28.3, 25.5, 24.6, 22.8, 21.8, 21.5, 19.4, 18.0, 12.5. IR (cm^{-1}): 3318, 1742, 1650, 1547, 1506, 1244. HRMS (ESI) m/z: (M^+) *calcd* for ($C_{45}H_{63}N_7O_{10}S$): 893.4357, *found* 893.4357.

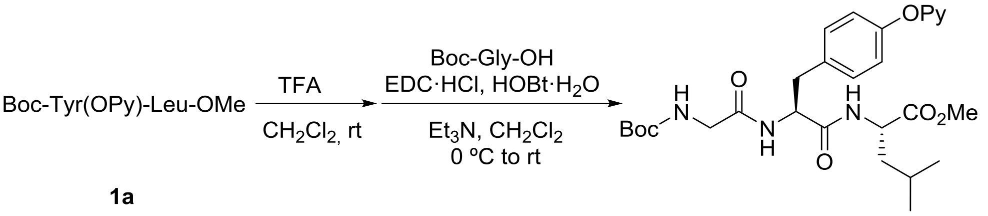

1a (1q)

Methyl ((*S*)-2-(2-((*tert*-butoxycarbonyl)amino)acetamido)-3-(4-(pyridin-2-yloxy)phenyl)propanoyl)-*L*-leucinate (1q). A solution of Boc-Tyr(OPy)-Leu-OMe (2.0 mmol, 971 mg) in dichloromethane was treated with trifluoroacetic acid (20 mmol, 1.5 mL) and stirred for 5 h. After evaporation of the solvent, the resulting crude was diluted with EtOAc and washed with a saturated aqueous solution of $NaHCO_3$. After evaporation of the solvent, the so-obtained crude (without further purification) was dissolved in dichloromethane (10 mL) at 0 ºC. EDC·HCl (2.4 mmol, 460 mg), HOBt (2.4 mmol, 323 mg), Boc-Gly-OH (2.4 mmol, 420 mg) and triethylamine (3.0 mmol, 0.45 mL) were subsequently added and stirred at room temperature overnight. The resulting solution was washed with water and extracted with dichloromethane. The solvent was removed under reduced pressure and the corresponding product was purified by flash chromatography (Hexane:EtOAc, 1:1) to provide 663 mg (61% yield) of **1q** as a white solid. Mp 75-76 °C. ^1H NMR (400 MHz, $CDCl_3$) δ 8.13 (dd, *J* = 5.0, 1.4 Hz, 1H), 7.63 (t, *J* = 6.8 Hz, 1H), 7.19 (d, *J* = 8.4 Hz, 2H), 7.10 (s, 1H), 6.99 (d, *J* = 8.4 Hz, 2H), 6.97 – 6.93 (m, 2H), 6.84 (d, *J* = 8.3 Hz, 1H), 5.56 (s, 1H), 4.77 (q, *J* = 6.9 Hz, 1H), 4.56 – 4.43 (m, 1H), 3.78 – 3.70 (m, 2H), 3.66 (s, 3H), 3.05 (d, *J* = 6.6 Hz, 2H), 1.64 – 1.45 (m, 3H), 1.39 (s, 9H), 0.85 (d, *J* = 5.7 Hz, 6H). ^{13}C NMR (101 MHz, $CDCl_3$) δ 172.7, 170.5, 169.6, 163.6, 156.0, 153.0, 147.5, 139.4, 132.6, 130.6, 121.1, 118.4, 111.4, 80.0, 54.0, 52.2, 50.9, 44.1, 41.0, 37.4, 28.2, 24.6, 22.6, 21.8. IR (cm^{-1}): 3292, 1645, 1242, 1152, 1505, 1427. HRMS (ESI) m/z: (M^+) *calcd* for ($C_{28}H_{38}N_4O_7$): 542.2740, *found* 542.2750.

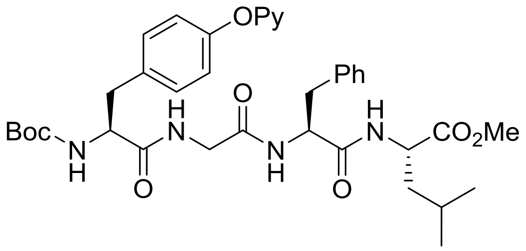

(1r)

Methyl [(*S*)-2-((*tert*-butoxycarbonyl)amino)-3-(4-(pyridin-2-yloxy)phenyl) propanoyl]glycyl-*L*-phenylalanyl-*L*-leucinate (1r). The title compound was synthesized through iterative hydrolysis employing LiOH and posterior coupling with the corresponding amino acid methyl esther (Gly, Phe, and Leu, in this order), starting from Boc-Tyr(OPy)-OMe (5.0 mmol, 1.9 g). Purification by flash chromatography (EtOAc) provided 746 mg (22% yield) of **1r** as a brown solid. Mp 84-85 °C. ^1H NMR (300 MHz, $CDCl_3$) δ 8.14 (d, *J* = 4.9 Hz, 1H), 7.64 (t, *J* = 6.8 Hz, 1H), 7.27 – 7.13 (m, 9H), 7.04 – 6.97 (m, 2H), 6.95 (d, *J* = 5.2 Hz, 2H), 6.84 (d, *J* = 8.3 Hz, 1H), 5.41 (d, *J* = 6.5 Hz, 1H), 4.80 (q, *J* = 7.1 Hz, 1H), 4.54 (q, *J* = 6.8, 5.3 Hz, 1H), 4.49 – 4.38 (m, 1H), 3.96 – 3.79

(m, 2H), 3.67 (s, 3H), 3.16 – 3.06 (m, 2H), 3.06 – 2.98 (m, 1H), 3.00 – 2.85 (m, 1H), 1.65 – 1.46 (m, 3H), 1.37 (s, 9H), 0.86 (d, *J* = 5.7 Hz, 6H). ^{13}C NMR (75 MHz, $CDCl_3$) δ 173.0, 172.1, 170.7, 168.6, 163.6, 155.6, 153.0, 147.6, 139.4, 136.4, 133.0, 130.5, 129.3, 128.4, 126.8, 121.1, 118.4, 111.4, 80.0, 55.6, 54.4, 52.2, 50.8, 43.0, 41.0, 38.2, 37.9, 28.3, 24.7, 22.7, 21.8. IR (cm^{-1}): 3290, 1639, 1506, 1428, 1244, 1162. HRMS (ESI) m/z: (M^+) *calcd* for ($C_{37}H_{47}N_5O_8$): 689.3425, *found* 689.3425.

4.-Pd-Catalyzed C(sp^2)-H Acylation of Tyr-Containing Compounds

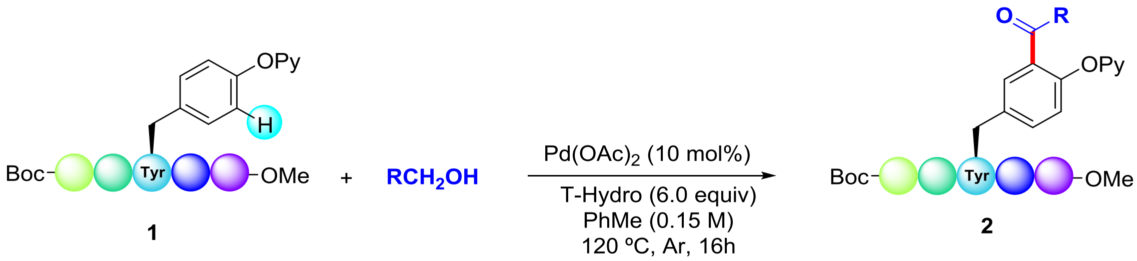

General Procedure A (Acetylation): A reaction tube containing a stirring bar was charged with the corresponding peptide (0.15 mmol) and $Pd(OAc)_2$ (10 mol %). The reaction tube was then evacuated and back-filled with dry argon (this sequence was repeated up to three times). Then, a commercially available solution of *tert*-butyl hydroperoxide (70 wt % in water) (0.90 mmol), EtOH (3.75 mmol) and toluene (1 mL) were added by syringe under argon atmosphere. The reaction tube was next warmed up to the corresponding temperature in a heating block and stirred for 16 hours. The mixture was then allowed to warm to room temperature, diluted with EtOAc and washed with aq. $NaHCO_3$ (20 mL). The aqueous layer was extracted with EtOAc (3 x 20 mL), dried over $MgSO_4$ and evaporated under vacuum. The resulting crude was then purified by column chromatography to afford the corresponding product.

General Procedure B: A reaction tube containing a stirring bar was charged with the corresponding peptide (0.15 mmol) and $Pd(OAc)_2$ (10 mol %). The reaction tube was then evacuated and back-filled with dry argon (this sequence was repeated up to three times). Then, a commercially available solution of *tert*-butyl hydroperoxide (70 wt % in water) (0.90 mmol), the corresponding alcohol (0.75 mmol) and toluene (1 mL) were added by syringe under argon atmosphere. The reaction tube was next warmed up to the corresponding temperature in a heating block and stirred for 16 hours. The mixture was then allowed to warm to room temperature, diluted with EtOAc and washed with aq. $NaHCO_3$ (20 mL). The aqueous layer was extracted with EtOAc (3 x 20 mL), dried over $MgSO_4$ and evaporated under vacuum. The resulting crude was then purified by column chromatography to afford the corresponding product.

General Procedure C: A reaction tube containing a stirring bar was charged with the corresponding peptide (0.15 mmol) and $Pd(OAc)_2$ (10 mol %). The reaction tube was then evacuated and back-filled with dry argon (this sequence was repeated up to three

times). Then, a commercially available solution of *tert*-butyl hydroperoxide (70 wt % in water) (0.60 mmol), the corresponding benzyl alcohol (0.45 mmol) and toluene (1 mL) were added by syringe under argon atmosphere. The reaction tube was next warmed up to the corresponding temperature in a heating block and stirred for 16 hours. The mixture was then allowed to warm to room temperature, diluted with EtOAc and washed with aq. $NaHCO_3$ (20 mL). The aqueous layer was extracted with EtOAc (3 x 20 mL), dried over $MgSO_4$ and evaporated under vacuum. The resulting crude was then purified by column chromatography to afford the corresponding product.

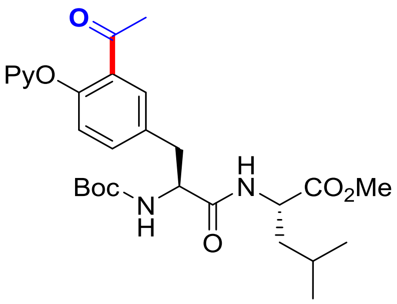

(2aa)

Methyl [(*S*)-3-(3-acetyl-4-(pyridin-2-yloxy)phenyl)-2-((*tert*-butoxycarbonyl)amino) propanoyl]-*L*-leucinate (2aa). Following the general procedure A, using **1a** (0.15 mmol, 73 mg) and EtOH (3.75 mmol, 0.22 mL) provided 47 mg (60% yield) of **2aa** as a colorless oil. Column chromatography (Hex/EtOAc 1:1). ^1H NMR (400 MHz, $CDCl_3$) δ 8.12 (dd, *J* = 5.1, 1.9 Hz, 1H), 7.76 – 7.62 (m, 2H), 7.38 (dd, *J* = 8.4, 2.4 Hz, 1H), 7.06 – 6.88 (m, 3H), 6.88 – 6.70 (m, 1H), 5.33 (d, *J* = 8.4 Hz, 1H), 4.60 – 4.54 (m, 1H), 4.43 – 4.41 (m, 1H), 3.69 (s, 3H), 3.15 (dd, *J* = 14.1, 6.2 Hz, 1H), 3.02 (dd, *J* = 13.9, 7.4 Hz, 1H), 2.48 (s, 3H), 1.61 – 1.57 (m, 3H), 1.39 (s, 9H), 0.88 (dd, *J* = 6.0, 3.8 Hz, 6H). ^{13}C NMR (101 MHz, $CDCl_3$) δ 198.3, 173.0, 171.0, 163.0, 155.4, 151.8, 147.7, 139.8, 134.5, 133.6, 131.5, 131.1, 123.0, 118.9, 111.6, 80.1, 55.3, 52.3, 50.7, 41.3, 37.3, 30.8, 28.2, 24.7, 22.8, 21.8. IR (cm^{-1}): 3305, 1743, 1665, 1657, 1427, 1160, 776. HRMS (ESI) m/z: (M^+) *calcd* for ($C_{28}H_{37}N_3O_7$): 527.2632, *found* 527.2638. *This reaction was also performed in a higher scale: the use of* ***1a*** *(2.06 mmol, 1.00 g), EtOH (20 equiv, 2.40 mL) and TBHP (5.0 equiv, 1.58 mL) in toluene (14 mL) provided 670 mg (62% yield) of* ***2aa*** *as a colorless oil.*

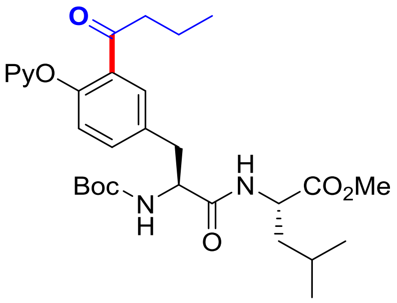

(2ab)

Methyl [(*S*)-2-((*tert*-butoxycarbonyl)amino)-3-(3-butyryl-4-(pyridin-2-yloxy) phenyl)propanoyl]-*L*-leucinate (2ab). Following the general procedure B, using **1a** (0.15 mmol, 73 mg) and *n*-BuOH (0.75 mmol, 69 µL) provided 61.5 mg (74% yield) of **2ab** as a colorless oil. Column chromatography (Hex/EtOAc 1:1). ^{1}H NMR (400 MHz, $CDCl_3$) δ 8.15 – 8.07 (m, 1H), 7.78 – 7.66 (m, 1H), 7.58 (d, *J* = 2.3 Hz, 1H), 7.37 (dd, *J* = 8.3, 2.3 Hz, 1H), 7.24 (d, *J* = 13.7 Hz, 1H), 7.08 – 6.91 (m, 3H), 6.40 (dd, *J* = 27.0, 8.3 Hz, 1H), 5.15 – 4.91 (m, 1H), 4.58 (td, *J* = 8.4, 4.8 Hz, 1H), 4.44 – 4.21 (m, 1H), 3.70 (s, 3H), 3.23 – 2.99 (m, 2H), 2.83 (t, *J* = 7.3 Hz, 2H), 1.68 – 1.46 (m, 5H), 1.42 (s, 9H), 0.90 (dd, *J* = 6.2, 3.9 Hz, 6H), 0.83 (t, *J* = 7.4 Hz, 3H). ^{13}C NMR (75 MHz, $CDCl_3$) δ 201.4, 172.9, 171.0, 163.1, 155.4, 151.1, 147.6, 139.7, 133.8, 133.6, 133.0, 132.1, 130.7, 122.9, 121.1, 118.8, 111.6, 80.1, 55.3, 52.2, 50.7, 44.5, 41.2, 37.3, 28.2, 24.6, 22.7, 21.8, 17.4, 13.6. IR (cm^{-1}): 3308, 1743, 1657, 1465, 1264, 729. HRMS (ESI) m/z: (M^{+}) *calcd* for ($C_{28}H_{37}N_3O_7$): 527.2632, *found* 527.2638. *This reaction was also performed in a higher scale: the use of* ***1a*** *(2.06 mmol, 1.00 g), n-BuOH (5.0 equiv, 0.95 mL) and TBHP (5.0 equiv, 1.58 mL) in toluene (14 mL) provided 843 mg (74% yield) of* ***2ab*** *as a colorless oil.*

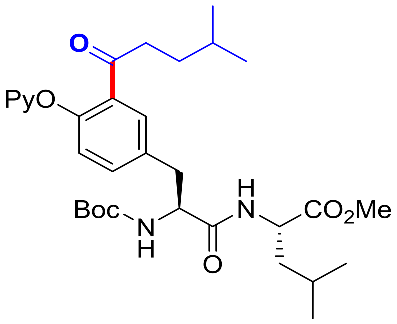

(2ac)

Methyl [(*S*)-2-((*tert*-butoxycarbonyl)amino)-3-(3-(4-methylpentanoyl)-4-(pyridin-2-yloxy)phenyl)propanoyl]-*L*-leucinate (2ac). Following the general procedure B, using **1a** (0.15 mmol, 73 mg) and 4-methylpentan-1-ol (0.75 mmol, 93 µL) provided 64.4 mg (74% yield) of **2ac** as a white solid. Mp 91-92 ºC. Column chromatography (Hex/EtOAc 1:1). ^{1}H NMR (400 MHz, $CDCl_3$) δ 8.13 (ddd, *J* = 5.0, 2.0, 0.8 Hz, 1H), 7.71 (ddd, *J* = 8.2, 7.2, 2.0 Hz, 1H), 7.58 (d, *J* = 2.3 Hz, 1H), 7.37 (dd, *J* = 8.4, 2.3 Hz, 1H), 7.07 – 6.97

(m, 2H), 6.94 (dt, *J* = 8.3, 0.9 Hz, 1H), 6.46 (d, *J* = 8.2 Hz, 1H), 5.09 (d, *J* = 8.3 Hz, 1H), 4.58 (td, *J* = 8.5, 4.9 Hz, 1H), 4.37 (d, *J* = 8.3 Hz, 1H), 3.70 (s, 3H), 3.23 – 2.94 (m, 2H), 2.94 – 2.69 (m, 2H), 1.64 – 1.44 (m, 6H), 1.41 (s, 9H), 0.90 (dd, *J* = 6.1, 3.6 Hz, 6H), 0.81 – 0.70 (m, 6H). ^{13}C NMR (101 MHz, $CDCl_3$) δ 201.9, 173.0, 170.9, 163.2, 155.5, 151.3, 147.8, 139.8, 133.9, 133.6, 132.4, 130.9, 123.2, 119.0, 111.8, 80.5, 55.5, 52.4, 50.9, 41.6, 41.0, 37.3, 32.9, 28.3, 27.8, 24.8, 22.9, 22.4, 22.0. IR (cm^{-1}): 3322, 1747, 1691, 1655, 1524, 1169, 778. HRMS (ESI) m/z: (M$^+$) *calcd* for ($C_{32}H_{45}N_3O_7$): 583.3258, *found* 583.3265.

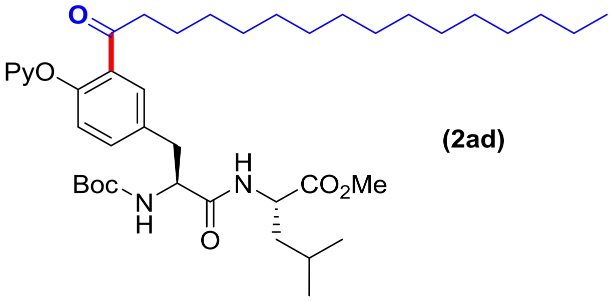

(2ad)

Methyl [(*S*)-2-((*tert*-butoxycarbonyl)amino)-3-(3-palmitoyl-4-(pyridin-2-yloxy) phenyl)propanoyl]-*L*-leucinate (2ad). Following the general procedure B, using **1a** (0.15 mmol, 73 mg) and palmityl alcohol (0.75 mmol, 181 mg) provided 76.6 mg (71% yield) of **2ad** as a yellowish oil. Column chromatography (Hex/EtOAc 7:3). ^{1}H NMR (400 MHz, $CDCl_3$) δ 8.14 (dd, *J* = 4.9, 2.0 Hz, 1H), 7.75 – 7.66 (m, 1H), 7.59 (d, *J* = 2.3 Hz, 1H), 7.37 (dd, *J* = 8.3, 2.3 Hz, 1H), 7.09 – 6.98 (m, 2H), 6.95 (d, *J* = 8.2 Hz, 1H), 6.37 (d, *J* = 8.2 Hz, 1H), 5.03 (d, *J* = 8.1 Hz, 1H), 4.61 – 4.56 (m, 1H), 4.37 – 4.35 (m, 1H), 3.71 (s, 3H), 3.21 – 2.95 (m, 2H), 2.84 (t, *J* = 7.4 Hz, 2H), 1.65 – 1.50 (m, 5H), 1.42 (s, 9H), 1.22 (d, *J* = 25.3 Hz, 24H), 0.95 – 0.81 (m, 9H). ^{13}C NMR (101 MHz, $CDCl_3$) δ 201.7, 173.0, 170.9, 163.2, 155.5, 151.3, 147.7, 139.8, 133.9, 133.6, 132.4, 130.9, 123.2, 118.9, 111.8, 80.5, 55.5, 52.4, 50.9, 42.9, 41.6, 37.3, 32.0, 29.8, 29.8, 29.7, 29.6, 29.5, 29.5, 29.3, 28.3, 24.8, 24.2, 22.9, 22.8, 21.9, 14.2. IR (cm^{-1}): 3305, 1744, 1680, 1656, 1428, 1241, 1165, 775. HRMS (ESI) m/z: (M$^+$) *calcd* for ($C_{42}H_{65}N_3O_7$): 723.4823, *found* 723.4826.

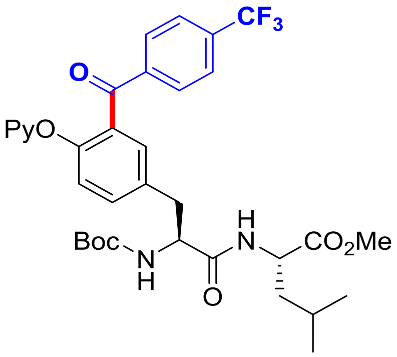

(2ae)

Methyl [(*S*)-2-((*tert*-butoxycarbonyl)amino)-3-(4-(pyridin-2-yloxy)-3-(4-(trifluoro-methyl)benzoyl)phenyl)propanoyl]-*L*-leucinate (2ae). Following the general procedure C, using **1a** (0.15 mmol, 73 mg), 4-(trifluoromethyl)benzyl alcohol (0.55 mmol, 82 μL) provided 62.3 mg (63% yield) of **2ae** as a white solid. Column chromatography (Hex/EtOAc 1:1). The spectroscopic data correspond to those previously reported in the literature.[1] ^{1}H NMR (400 MHz, $CDCl_3$) δ 7.96 (dd, *J* = 5.0, 1.9 Hz, 1H), 7.81 (d, *J* = 8.1 Hz, 2H), 7.56 (d, *J* = 8.2 Hz, 2H), 7.53 – 7.47 (m, 1H), 7.46 (dd, *J* = 8.3, 2.3 Hz, 1H), 7.42 (d, *J* = 2.2 Hz, 1H), 7.19 (d, *J* = 8.3 Hz, 1H), 6.88 (dd, *J* = 7.2, 5.0 Hz, 1H), 6.54 (d, *J* = 8.3 Hz, 1H), 6.43 (d, *J* = 8.3 Hz, 1H), 5.12 (d, *J* = 8.2 Hz, 1H), 4.64 – 4.51 (m, 1H), 4.38 (d, *J* = 7.9 Hz, 1H), 3.67 (s, 3H), 3.25 – 2.99 (m, 2H), 1.67 – 1.49 (m, 4H), 1.41 (s, 9H), 0.90 (dd, *J* = 6.1, 3.2 Hz, 6H). ^{13}C NMR (101 MHz, $CDCl_3$) δ 194.2, 173.0, 170.7, 162.7, 150.8, 146.9, 140.6, 139.7, 134.0, 133.9 (q, $J_{C\text{-}F}$ = 32.5 Hz), 133.6, 131.5, 131.3, 130.0, 125.1 (q, $J_{C\text{-}F}$ = 4.0 Hz), 123.3, 123.0 (q, $J_{C\text{-}F}$ = 272.6 Hz), 118.8, 111.5, 80.6, 55.6, 52.5, 50.9, 41.6, 37.4, 28.4, 24.8, 22.9, 21.9.

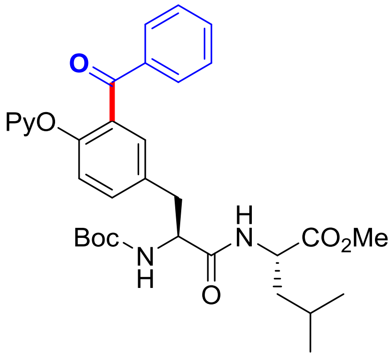

(2af)

Methyl [(*S*)-3-(3-benzoyl-4-(pyridin-2-yloxy)phenyl)-2-((*tert*-butoxycarbonyl) amino)propanoyl]-*L*-leucinate (2af). Following the general procedure C, using **1a** (0.15 mmol, 73 mg) and benzyl alcohol (0.54 mmol, 47 μL) provided 52 mg (59% yield) of **2af** as a white solid. Mp 61-62 ºC. Column chromatography (Hex/EtOAc 1:1). ^{1}H NMR (400 MHz, $CDCl_3$) δ 7.99 – 7.96 (m, 1H), 7.75 – 7.71 (m, 2H), 7.53 – 7.47 (m, 1H), 7.51 – 7.43 (m, 1H), 7.42 (dd, *J* = 8.3, 2.2 Hz, 1H), 7.38 (s, 1H), 7.33 (t, *J* = 7.7 Hz, 2H), 7.19

(d, J = 8.3 Hz, 1H), 6.86 (ddd, J = 7.1, 5.0, 0.9 Hz, 1H), 6.59 (d, J = 8.3 Hz, 1H), 6.44 (d, J = 8.3 Hz, 1H), 5.09 (d, J = 8.2 Hz, 1H), 4.58 (td, J = 8.6, 5.0 Hz, 1H), 4.43 – 4.29 (m, 1H), 3.67 (s, 3H), 3.17 (dd, J = 14.1, 6.5 Hz, 1H), 3.08 (dd, J = 14.3, 6.9 Hz, 1H), 1.64 – 1.55 (m, 2H), 1.54 – 1.48 (m, 1H), 1.41 (s, 9H), 0.91 (d, J = 3.6 Hz, 3H), 0.89 (d, J = 4.4 Hz, 3H). ^{13}C NMR (101 MHz, $CDCl_3$) δ 195.0, 172.9, 170.7, 162.9, 155.4, 150.5, 146.9, 140.0, 137.3, 133.1, 132.8, 132.2, 131.0, 129.8, 128.0, 122.9, 118.5, 111.4, 80.4, 55.4, 52.3, 50.7, 41.5, 37.1, 28.2, 24.7, 22.8, 21.8. IR (cm^{-1}): 3304, 2956, 1657, 1428, 1242, 1158. HRMS (ESI) m/z: (M^+) *calcd* for ($C_{33}H_{39}N_3O_7$): 589.2788, *found* 589.2799.

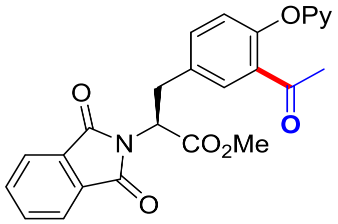

(2b)

Methyl (*S*)-3-[3-acetyl-4-(pyridin-2-yloxy)phenyl]-2-(1,3-dioxoisoindolin-2-yl) propanoate (2b). Following the general procedure A, using NPhth-Tyr(OPy)-OMe **(1b)** (0.15 mmol, 60 mg) *in PhCl as solvent* provided 39 mg (58% yield) of **2b** as a white solid. Mp 108-109 °C. Column chromatography (Hex/EtOAc 1:1). ^{1}H NMR (400 MHz, $CDCl_3$) δ 8.09 (dd, J = 5.1, 2.0 Hz, 1H), 7.79 (dd, J = 5.5, 3.1 Hz, 2H), 7.75 – 7.60 (m, 4H), 7.33 (dd, J = 8.4, 2.4 Hz, 1H), 7.01 – 6.93 (m, 2H), 6.92 – 6.82 (m, 1H), 5.15 (dd, J = 10.9, 5.4 Hz, 1H), 3.76 (s, 3H), 3.68 – 3.47 (m, 2H), 2.40 (s, 3H). ^{13}C NMR (101 MHz, $CDCl_3$) δ 198.1, 169.2, 167.5, 163.0, 151.8, 147.8, 139.8, 134.3, 133.8, 133.6, 131.7, 131.6, 130.6, 123.7, 123.2, 119.0, 111.7, 53.1, 53.0, 34.1, 30.7. IR (cm^{-1}): 1777, 1744, 1709, 1672, 1387, 1268, 859. HRMS (ESI) m/z: (M^+) *calcd* for ($C_{25}H_{20}N_2O_6$): 444.1321, *found* 444.1323.

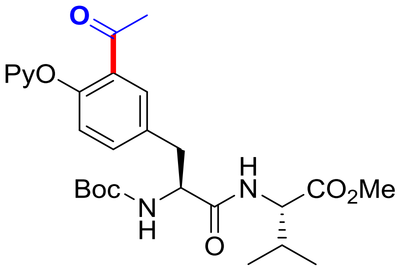

(2c)

Methyl [(*S*)-3-(3-acetyl-4-(pyridin-2-yloxy)phenyl)-2-((*tert*-butoxycarbonyl)amino) propanoyl)-*L*-valinate (2c). Following the general procedure A, using Boc-Tyr(OPy)Val-OMe[1] **(1c)** (0.15 mmol, 71 mg) and EtOH (3.75 mmol, 0.22 mL) provided 40 mg (52% yield) of **2c** as a yellowish oil. Column chromatography (Hex/EtOAc 1:1). ^{1}H NMR (400 MHz, $CDCl_3$) δ 8.13 (dd, J = 5.1, 2.0 Hz, 1H), 7.76 – 7.61 (m, 2H), 7.38

(dd, *J* = 8.4, 2.3 Hz, 1H), 7.08 – 6.97 (m, 2H), 6.94 (d, *J* = 8.3 Hz, 1H), 6.62 (d, *J* = 8.8 Hz, 1H), 5.18 (d, *J* = 8.2 Hz, 1H), 4.48 (dd, *J* = 8.7, 5.0 Hz, 1H), 4.44 – 4.30 (m, 1H), 3.69 (s, 3H), 3.15 (dd, *J* = 14.0, 6.4 Hz, 1H), 3.04 (dd, *J* = 14.1, 7.3 Hz, 1H), 2.48 (s, 3H), 2.13 (ddd, *J* = 13.8, 6.9, 5.1 Hz, 2H), 1.40 (s, 9H), 0.86 (dd, *J* = 10.3, 6.9 Hz, 6H). ^{13}C NMR (101 MHz, $CDCl_3$) δ 198.4, 172.0, 171.0, 163.1, 155.5, 151.9, 147.8, 139.9, 134.4, 133.6, 131.7, 131.1, 123.2, 119.0, 111.8, 80.4, 57.3, 55.6, 52.2, 37.2, 31.3, 30.9, 28.3, 18.9, 17.8. IR (cm^{-1}): 3306, 1740, 1679, 1655, 1427, 1239, 775. HRMS (ESI) m/z: (M^+) *calcd* for ($C_{27}H_{35}N_3O_7$): 513.2475, *found* 513.2478.

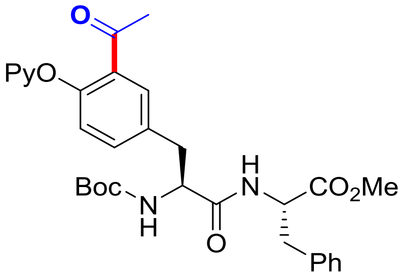

(2da)

Methyl [(*S*)-3-(3-acetyl-4-(pyridin-2-yloxy)phenyl)-2-((*tert*-butoxycarbonyl)amino) propanoyl)-*L*-phenylalaninate (2da). Following the general procedure A, using Boc-Tyr(OPy)-Phe-OMe[1] **(1d)** (0.15 mmol, 78 mg) and EtOH (3.75 mmol, 0.22 mL) provided 40.3 mg (48% yield) of **2da** as a colorless oil. Column chromatography (Hex/EtOAc 1:1). ^{1}H NMR (400 MHz, $CDCl_3$) δ 8.16 (dd, *J* = 5.1, 1.9 Hz, 1H), 7.75 (ddd, *J* = 8.3, 7.2, 2.0 Hz, 1H), 7.67 (d, *J* = 2.3 Hz, 1H), 7.46 – 7.35 (m, 1H), 7.32 – 7.19 (m, 3H), 7.12 – 7.02 (m, 4H), 6.99 (d, *J* = 8.3 Hz, 1H), 6.40 (d, *J* = 8.2 Hz, 1H), 5.02 (s, 1H), 4.93 – 4.78 (m, 1H), 4.38 – 4.36 (m, 1H), 3.72 (s, 3H), 3.17 – 3.03 (m, 4H), 2.53 (s, 3H), 1.44 (s, 9H). ^{13}C NMR (101 MHz, $CDCl_3$) δ 198.4, 171.5, 170.6, 163.1, 155.3, 152.0, 147.8, 139.9, 135.7, 134.4, 133.5, 131.8, 131.1, 129.3, 128.8, 127.3, 123.3, 119.1, 111.8, 80.4, 55.5, 53.4, 53.2, 52.5, 38.0, 37.6, 30.9, 28.3. IR (cm^{-1}): 3318, 1742, 1685, 1655, 1427, 1264, 1210, 699. HRMS (ESI) m/z: (M^+) *calcd* for ($C_{31}H_{35}N_3O_7$): 561.2475, *found* 561.2479.

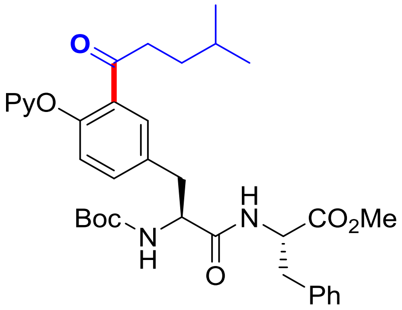

(2db)

Methyl [(*S*)-2-((*tert*-butoxycarbonyl)amino)-3-(3-(4-methylpentanoyl)-4-(pyridin-2-yloxy)phenyl)propanoyl]-*L*-phenylalaninate (2db). Following the general procedure

B, using Boc-Tyr(OPy)-Phe-OMe[1] **(1d)** (0.15 mmol, 78 mg) and 4-methylpentan-1-ol (0.75 mmol, 93 μL) *in PhCl as solvent* provided 39 mg (42% yield) of **2db** as a colorless oil. ^{1}H NMR (300 MHz, $CDCl_3$) δ 8.15 (d, *J* = 3.0 Hz, 1H), 7.73 (ddd, *J* = 8.2, 7.3, 2.0 Hz, 1H), 7.58 (d, *J* = 2.2 Hz, 1H), 7.36 (dd, *J* = 8.3, 2.3 Hz, 1H), 7.31 – 7.22 (m, 3H), 7.12 – 6.90 (m, 5H), 6.40 (d, *J* = 7.7 Hz, 1H), 5.00 (s, 1H), 4.83 (q, *J* = 6.7 Hz, 1H), 4.35 (m, 1H), 3.71 (s, 3H), 3.26 – 3.03 (m, 4H), 2.89 – 2.71 (m, 2H), 1.61 – 1.35 (m, 12H), 0.81 (d, *J* = 6.4 Hz, 6H). ^{13}C NMR (75 MHz, $CDCl_3$) δ 202.4, 172.0, 171.1, 163.8, 151.9, 148.3, 140.3, 136.3, 134.3, 134.1, 133.1, 131.4, 129.9, 129.2, 127.8, 123.8, 119.5, 112.3, 80.4, 56.1, 53.9, 53.0, 41.5, 38.5, 38.1, 33.5, 28.9, 28.4, 22.9. IR (cm^{-1}): 3327, 2955, 1677, 1428, 1264, 1167, 970, 730. IR (cm^{-1}): 3318, 1742, 1685, 1655, 1427, 1264, 1210, 699. HRMS (ESI) m/z: (M^+) *calcd* for ($C_{35}H_{43}N_3O_7$): 617.3101, *found* 617.3105.

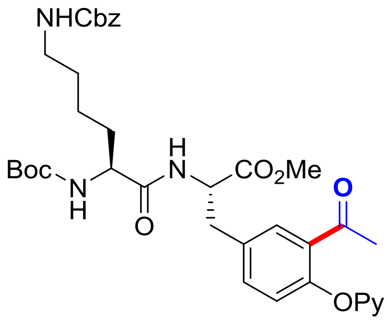

(2e)

Methyl (*S*)-3-[3-acetyl-4-(pyridin-2-yloxy)phenyl]-2-[(*S*)-6-(((benzyloxy)carbonyl) amino)-2-((*tert*-butoxycarbonyl)amino)hexanamido]propanoate (2e). Following the general procedure A, using Boc-Lys(Cbz)-Tyr(OPy)-OMe[1] **(1e)** (0.15 mmol, 95 mg) in *PhCl as solvent* provided 42.4 mg (41% yield) of **2e** as a colorless oil. Column chromatography (Hex/EtOAc 3:7). ^{1}H NMR (400 MHz, $CDCl_3$) δ 8.18 (d, *J* = 4.9 Hz, 1H), 7.83 – 7.71 (m, 1H), 7.59 (d, *J* = 2.3 Hz, 1H), 7.46 – 7.25 (m, 6H), 7.11 – 6.94 (m, 3H), 6.80 (d, *J* = 7.9 Hz, 1H), 5.37 (d, *J* = 7.9 Hz, 1H), 5.17 – 5.13 (m, 1H), 5.09 (s, 2H), 4.89 (q, *J* = 6.3 Hz, 1H), 4.12 – 4.10 (m, 1H), 3.74 (s, 3H), 3.35 – 3.01 (m, 4H), 2.52 (s, 3H), 1.87 – 1.80 (m, 2H), 1.72 – 1.47 (m, 4H), 1.42 (s, 9H). ^{13}C NMR (101 MHz, $CDCl_3$) δ 198.6, 172.0, 171.6, 163.0, 156.7, 155.9, 152.3, 147.8, 140.1, 136.7, 134.4, 132.7, 131.4, 131.2, 128.6, 128.2, 128.1, 123.1, 119.2, 112.0, 80.1, 66.7, 54.4, 53.0, 52.6, 40.4, 37.1, 31.6, 31.0, 29.4, 28.3, 22.5. IR (cm^{-1}): 3320, 1742, 1673, 1465, 1239, 1163. HRMS (ESI) m/z: (M^+) *calcd* for ($C_{36}H_{44}N_4O_9$): 676.3108, *found* 676.3109.

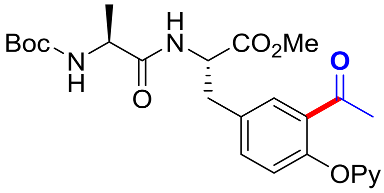

(2f)

Methyl (*S*)-3-(3-acetyl-4-(pyridin-2-yloxy)phenyl)-2-((*S*)-2-((*tert*-butoxycarbonyl) amino)propanamido)propanoate (2f). Following the general procedure A, using Boc-Ala-Tyr(OPy)-OMe[1] **(1f)** (0.15 mmol, 66 mg) and EtOH (3.75 mmol, 0.22 mL) provided 54 mg (74% yield) of **2f** as a colorless oil. Column chromatography (Hex/EtOAc 1:1). ^{1}H NMR (400 MHz, $CDCl_3$) δ 8.24 – 8.11 (m, 1H), 7.83 – 7.70 (m, 1H), 7.58 (d, *J* = 2.3 Hz, 1H), 7.35 – 7.26 (m, 1H), 7.06 (t, *J* = 6.8 Hz, 2H), 7.01 (d, *J* = 8.3 Hz, 1H), 6.81 (d, *J* = 7.8 Hz, 1H), 5.27 (s, 1H), 4.89 (dt, *J* = 7.7, 5.7 Hz, 1H), 4.19 – 4.16 (m, 1H), 3.77 (s, 3H), 3.28 (dd, *J* = 13.9, 5.6 Hz, 1H), 3.11 (dd, *J* = 13.9, 5.9 Hz, 1H), 2.53 (s, 3H), 1.43 (s, 9H), 1.36 (d, *J* = 7.1 Hz, 3H). ^{13}C NMR (101 MHz, $CDCl_3$) δ 198.7, 172.6, 171.5, 163.0, 155.7, 152.3, 147.8, 140.0, 134.5, 132.8, 131.2, 123.1, 119.2, 112.0, 80.2, 53.0, 52.7, 50.2, 37.1, 31.1, 28.4, 18.0. IR (cm^{-1}): 3306, 1742, 1668, 1427, 1239, 1163, 777. HRMS (ESI) m/z: (M^+) *calcd* for ($C_{25}H_{31}N_3O_7$): 485.2162, *found* 485.2166.

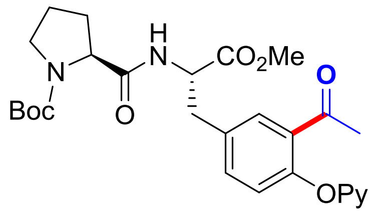

(2g)

***tert*-Butyl (*S*)-2-[((*S*)-3-(3-acetyl-4-(pyridin-2-yloxy)phenyl)-1-methoxy-1-oxopropan-2-yl)carbamoyl]pyrrolidine-1-carboxylate (2g).** Following the general procedure A, using **1g** (0.15 mmol, 79 mg) and EtOH (3.75 mmol, 0.22 mL) provided 38 mg (50% yield) of **2g** as a yellow oil. Column chromatography (Hex/EtOAc 1:1). ^{1}H NMR (300 MHz, $CDCl_3$) δ 8.17 – 8.09 (m, 1H), 7.71 (t, *J* = 7.7 Hz, 1H), 7.58 (dd, *J* = 7.7, 2.1 Hz, 1H), 7.34 – 7.25 (m, 1H), 7.05 – 6.97 (m, 2H), 6.94 (d, *J* = 8.2 Hz, 1H), 4.97 – 4.73 (m, 1H), 4.32 – 4.12 (m, 1H), 3.72 (s, 3H), 3.47 – 3.28 (m, 2H), 3.13 (dtd, *J* = 36.1, 14.1, 6.1 Hz, 2H), 2.49 (s, 3H), 2.30 – 1.73 (m, 4H), 1.41 (s, 9H). ^{13}C NMR (75 MHz, $CDCl_3$) δ 198.0, 171.5, 171.4, 162.9, 152.1, 147.7, 147.7, 139.7, 134.1, 132.8, 131.5, 130.8, 123.0, 118.9, 111.6, 80.4, 60.0, 53.1, 52.4, 46.9, 37.3, 30.8, 29.6, 28.3, 24.4. IR (cm^{-1}): 3305, 2976, 1744, 1678, 1428, 1238, 1191. HRMS (ESI) m/z: (M^+) *calcd* for ($C_{27}H_{33}N_3O_7$): 511.2319, *found* 511.2325.

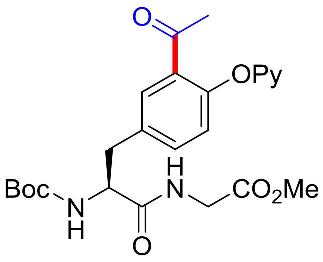

(2h)

Methyl (*S*)-[3-(3-acetyl-4-(pyridin-2-yloxy)phenyl)-2-((*tert*-butoxycarbonyl)amino) propanoyl]glycinate (2h). Following the general procedure A, using **1h** (0.15 mmol, 79 mg) and EtOH (3.75 mmol, 0.22 mL) provided 37 mg (53% yield) of **2h** as a white solid. Mp 57-58 ºC. Column chromatography (Hex/EtOAc 1:1). ^{1}H NMR (400 MHz, $CDCl_3$) δ 8.15 (dd, *J* = 5.0, 1.9 Hz, 1H), 7.72 (ddd, *J* = 8.8, 7.4, 2.0 Hz, 1H), 7.67 (d, *J* = 2.3 Hz, 1H), 7.40 (dd, *J* = 8.4, 2.3 Hz, 1H), 7.05 (d, *J* = 8.4 Hz, 1H), 7.02 (dd, *J* = 7.3, 5.1 Hz, 1H), 6.97 (d, *J* = 8.3 Hz, 1H), 6.61 (s, 1H), 5.08 (s, 1H), 4.48 – 4.37 (m, 1H), 4.06 (dd, *J* = 18.3, 5.4 Hz, 1H), 3.98 (dd, *J* = 18.3, 5.2 Hz, 1H), 3.74 (s, 3H), 3.18 (dd, *J* = 14.0, 6.3 Hz, 1H), 3.07 (dd, *J* = 14.0, 7.2 Hz, 1H), 2.51 (s, 3H), 1.41 (s, 9H). ^{13}C NMR (101 MHz, $CDCl_3$) δ 198.3, 171.2, 169.9, 163.0, 155.4, 151.9, 147.7, 139.8, 134.3, 133.4, 131.7, 131.0, 123.2, 119.0, 111.7, 80.5, 55.4, 52.4, 41.2, 37.5, 30.8, 28.2. IR (cm^{-1}): 3238, 1741, 1656, 1427, 1240, 1155. HRMS (ESI) m/z: (M$^+$) *calcd* for ($C_{24}H_{29}N_3O_7$): 471.2006, *found* 471.2004.

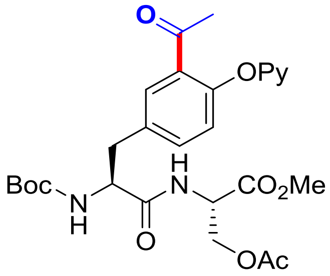

(2i)

Methyl *O*-acetyl-*N*-[(*S*)-3-(3-acetyl-4-(pyridin-2-yloxy)phenyl]-2-[(*tert*-butoxycarbonyl)amino)propanoyl]-*L*-serinate (2i). Following the general procedure A, using **1i** (0.15 mmol, 75 mg) and EtOH (3.75 mmol, 0.22 mL) provided 42 mg (51% yield) of **2i** as a white solid. Mp 148-149 ºC. Column chromatography (Hex/EtOAc 1:1). ^{1}H NMR (300 MHz, $CDCl_3$) δ 8.17 – 8.10 (m, 1H), 7.75 – 7.67 (m, 1H), 7.66 (t, *J* = 2.3 Hz, 1H), 7.37 (d, *J* = 8.3 Hz, 1H), 7.06 – 7.00 (m, 2H), 6.99 – 6.91 (m, 2H), 6.87 (d, *J* = 7.8 Hz, 0H), 5.15 (s, 1H), 4.79 (dt, *J* = 7.4, 3.5 Hz, 1H), 4.47 – 4.37 (m, 2H), 4.27 (ddd, *J* = 28.7, 11.4, 3.5 Hz, 1H), 3.73 (s, 3H), 3.15 (dd, *J* = 14.6, 7.4 Hz, 1H), 3.05 (dd, *J* = 13.9, 7.1 Hz, 1H), 2.49 (s, 3H), 2.00 (s, 3H), 1.40 (s, 9H). ^{13}C NMR (75 MHz, $CDCl_3$) δ 198.2, 171.0, 170.5, 169.4, 163.0, 155.2, 151.9, 147.7, 139.7, 134.2, 133.2, 131.6, 130.9, 123.1, 118.9, 111.7, 80.4, 63.6, 55.3, 52.8, 51.6, 37.4, 30.7, 28.2, 20.5. IR (cm^{-1}): 3262, 1746, 1650, 1217. HRMS (ESI) m/z: (M$^+$) *calcd* for ($C_{27}H_{33}N_3O_9$): 543.2217, *found* 543.2209.

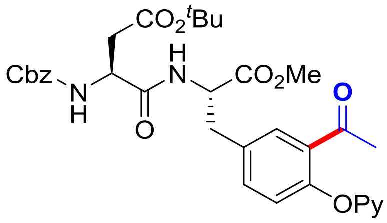

(2j)

***tert*-Butyl (*S*)-4-[((*S*)-3-(3-acetyl-4-(pyridin-2-yloxy)phenyl)-1-methoxy-1-oxopropan-2-yl)amino]-3-[((benzyloxy)carbonyl)amino]-4-oxobutanoate (2j).** Following the general procedure A, using **1j** (0.15 mmol, 87 mg) and EtOH (3.75 mmol, 0.22 mL) provided 49 mg (53% yield) of **2j** as a white solid. Mp 49-50 ºC. Column chromatography (Hex/EtOAc 1:1). ^{1}H NMR (300 MHz, $CDCl_3$) δ 8.14 (dd, *J* = 5.3, 1.6 Hz, 1H), 7.72 (ddd, *J* = 8.3, 7.3, 2.0 Hz, 1H), 7.61 (dd, *J* = 12.0, 2.2 Hz, 1H), 7.37 – 7.26 (m, 5H), 7.01 (dd, *J* = 8.2, 2.7 Hz, 3H), 6.95 (d, *J* = 8.0 Hz, 1H), 5.99 (dd, *J* = 40.5, 8.0 Hz, 1H), 5.18 – 5.03 (m, 2H), 4.83 (ddd, *J* = 13.4, 6.0 Hz, 1H), 4.62 – 4.45 (m, 1H), 3.73 (s, 3H), 3.18 (dd, *J* = 13.7, 5.4 Hz, 1H), 3.06 (dd, *J* = 13.9, 6.1 Hz, 1H), 2.86 (dd, *J* = 17.1, 4.5 Hz, 1H), 2.68 – 2.55 (m, 1H), 2.50 (s, 3H), 1.41 (s, 9H). ^{13}C NMR (75 MHz, $CDCl_3$) δ 198.2, 171.1, 170.8, 170.3, 163.0, 156.2, 152.1, 147.8, 139.7, 136.1, 134.3, 132.6, 131.6, 131.0, 128.5, 128.2, 128.0, 123.1, 118.9, 111.7, 81.8, 67.2, 53.2, 52.5, 51.0, 37.2, 37.0, 30.8, 28.0. IR (cm^{-1}): 3324, 1726, 1677, 1239, 1153. HRMS (ESI) m/z: (M^+) *calcd* for ($C_{33}H_{37}N_3O_9$): 619.2530, *found* 619.2523.

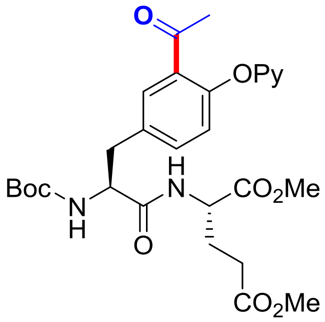

(2ka)

Dimethyl [(*S*)-3-(3-acetyl-4-(pyridin-2-yloxy)phenyl]-2-[(*tert*-butoxycarbonyl) amino)propanoyl]-*L*-glutamate (2ka). Following the general procedure A, using **1k** (0.15 mmol, 77 mg) and EtOH (3.75 mmol, 0.22 mL) provided 43 mg (52% yield) of **2ka** as a white solid. Mp 45-46 ºC. Column chromatography (Hex/EtOAc 1:1). ^{1}H NMR (400 MHz, $CDCl_3$) δ 8.14 (td, *J* = 5.0, 1.9 Hz, 1H), 7.72 (td, *J* = 7.8, 2.0 Hz, 1H), 7.66 (dd, *J* = 4.4, 2.3 Hz, 1H), 7.39 (d, *J* = 8.4 Hz, 1H), 7.06 (dd, *J* = 8.4, 2.2 Hz, 1H), 7.02 (dd, *J* = 7.2, 5.1 Hz, 1H), 6.97 (d, *J* = 8.3 Hz, 1H), 6.85 – 6.60 (m, 1H), 5.04 (s, 1H), 4.58 (ddd, *J* = 7.4 Hz, 1H), 4.47 – 4.30 (m, 1H), 3.72 (s, 3H), 3.64 (s, 3H), 3.15 (dt, *J* = 13.5, 6.6 Hz, 1H), 3.06 (dt, *J* = 13.4, 6.3 Hz, 1H), 2.51 (s, 3H), 2.41 – 2.25 (m, 2H), 2.25 – 2.07 (m,

1H), 2.02 – 1.88 (m, 1H), 1.42 (s, 9H). ^{13}C NMR (101 MHz, $CDCl_3$) δ 198.3, 173.1, 171.7, 170.9, 163.0, 155.3, 151.9, 147.7, 139.8, 134.3, 133.3, 131.7, 131.0, 123.3, 118.9, 111.7, 80.4, 55.5, 52.6, 51.8, 51.6, 37.3, 30.8, 29.8, 28.2, 27.2. IR (cm^{-1}): 3270, 1736, 1684, 1649, 1243, 1158. HRMS (ESI) m/z: (M$^+$) *calcd* for ($C_{28}H_{35}N_3O_9$): 557.2323, *found* 557.2376.

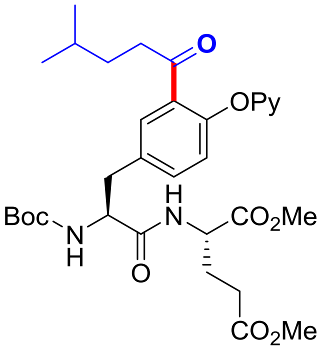

(2kb)

Dimethyl [(*S*)-2-((*tert*-butoxycarbonyl)amino)-3-(3-(4-methylpentanoyl)-4-(pyridin-2-yloxy)phenyl)propanoyl]-*L*-glutamate (2kb). Following the general procedure B, using **1k** (0.15 mmol, 77 mg) and 4-methylpentan-1-ol (0.75 mmol, 93 µL) provided 38 mg (52% yield) of **2kb** as a yellow oil. Column chromatography (Hex/EtOAc 1:1). ^{1}H NMR (400 MHz, $CDCl_3$) δ 8.16 – 8.09 (m, 1H), 7.72 (t, *J* = 7.7 Hz, 1H), 7.61 – 7.54 (m, 1H), 7.37 (d, *J* = 8.1 Hz, 1H), 7.06 (d, *J* = 8.3 Hz, 1H), 7.04 – 6.98 (m, 1H), 6.95 (d, *J* = 8.2 Hz, 1H), 6.86 – 6.65 (m, 1H), 5.03 (s, 1H), 4.66 – 4.51 (m, 1H), 4.44 – 4.31 (m, 1H), 3.72 (s, 3H), 3.64 (s, 3H), 3.21 – 3.11 (m, 1H), 3.11 – 3.00 (m, 4H), 2.87 – 2.79 (m, 2H), 2.39 – 2.26 (m, 2H), 2.24 – 2.11 (m, 1H), 2.00 – 1.89 (m, 1H), 1.49 – 1.35 (m, 12H), 0.79 (d, *J* = 6.1 Hz, 6H). ^{13}C NMR (101 MHz, $CDCl_3$) δ 201.7, 173.1, 171.8, 171.0, 163.1, 155.3, 151.2, 147.6, 139.7, 133.7, 133.3, 132.4, 130.7, 123.3, 118.8, 111.6, 80.4, 55.5, 52.6, 51.8, 51.7, 40.8, 37.3, 32.8, 29.8, 28.2, 27.7, 27.2, 27.0, 22.3. IR (cm^{-1}): 3323, 2955, 1739, 1675, 1428, 1242, 1167. HRMS (ESI) m/z: (M$^+$) *calcd* for ($C_{32}H_{43}N_3O_9$): 613.2999, *found* 613.3011.

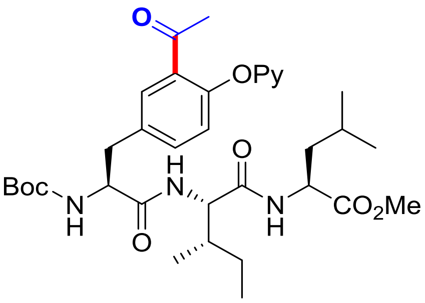

(2l)

Methyl [(*S*)-3-(3-acetyl-4-(pyridin-2-yloxy)phenyl)-2-((*tert*-butoxycarbonyl)amino) propanoyl)-*L*-isoleucyl-*L*-leucinate (2l). Following the general procedure A, using Boc-

Tyr(OPy)-Ile-Leu-OMe[1] **(1l)** (0.15 mmol, 90 mg) in *PhCl as solvent* provided 39 mg (37% yield) of **2l** as white solid. Mp 131-132 ºC. Column chromatography (Hex/EtOAc 1:1). ^{1}H NMR (400 MHz, $CDCl_3$) δ 8.12 (d, *J* = 5.0 Hz, 1H), 7.80 – 7.61 (m, 2H), 7.35 (dd, *J* = 8.4, 2.4 Hz, 1H), 7.08 – 6.72 (m, 5H), 5.41 (d, *J* = 8.0 Hz, 1H), 4.57 – 4.52 (m, 1H), 4.53 – 4.27 (m, 2H), 3.70 (s, 3H), 3.23 – 2.94 (m, 2H), 2.48 (s, 3H), 2.08 – 1.78 (m, 1H), 1.67 – 1.50 (m, 5H), 1.36 (s, 9H), 1.34 – 1.06 (m, 1H), 0.90 – 0.84 (m, 11H). ^{13}C NMR (101 MHz, $CDCl_3$) δ 198.4, 173.1, 171.3, 170.9, 163.1, 155.7, 152.0, 147.8, 139.9, 134.4, 133.7, 131.5, 131.2, 123.1, 119.0, 111.8, 80.3, 57.9, 55.6, 52.3, 50.9, 41.1, 37.4, 37.1, 30.9, 28.3, 24.9, 22.9, 22.0, 15.3, 11.4. IR (cm^{-1}): 3286, 1746, 1685, 1641, 1465, 1158. HRMS (ESI) m/z: (M^+) *calcd* for ($C_{34}H_{48}N_4O_8$): 640.3472, *found* 640.3479.

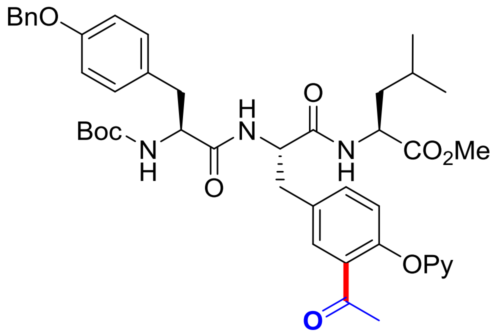

(2m)

Methyl [(*S*)-3-(3-acetyl-4-(pyridin-2-yloxy)phenyl]-2-[(*S*)-3-(4-(benzyloxy)phenyl)-2-((*tert*-butoxycarbonyl)amino)propanamido)propanoyl]-*L*-leucinate (2m). Following the general procedure A, using Boc-Tyr(OBn)-Tyr(OPy)-Leu-OMe **(1m)** (0.15 mmol, 111 mg) and EtOH (3.75 mmol, 0.22 mL) in *PhCl as solvent* provided 48 mg (41% yield) of **2m** as a white solid. Mp 130-131 ºC. Column chromatography (Hex/EtOAc 6:4). ^{1}H NMR (300 MHz, $CDCl_3$) δ 8.09 (ddd, *J* = 5.0, 2.0, 0.8 Hz, 1H), 7.70 (ddd, *J* = 8.3, 7.2, 2.0 Hz, 1H), 7.53 (d, *J* = 2.3 Hz, 1H), 7.44 – 7.28 (m, 6H), 7.16 – 7.07 (m, 3H), 7.05 – 6.84 (m, 6H), 6.70 (d, *J* = 8.2 Hz, 1H), 6.51 (d, *J* = 8.0 Hz, 1H), 5.09 (d, *J* = 7.2 Hz, 1H), 5.02 (s, 2H), 4.73 – 4.67 (m, 1H), 4.56 – 4.48 (m,1H), 4.33 – 4.26 (m, 1H), 3.70 (s, 3H), 3.18 – 2.99 (m, 3H), 2.88 (dd, *J* = 14.5, 7.8 Hz, 1H), 2.49 (s, 3H), 2.06 – 2.02 (m, 2H), 1.72 – 1.43 (m, 4H), 1.34 (s, 9H), 0.88 (d, *J* = 5.8 Hz, 6H). ^{13}C NMR (75 MHz, $CDCl_3$) δ 198.5, 172.8, 171.5, 171.0, 163.1, 158.0, 155.7, 152.0, 147.8, 139.9, 137.1, 134.6, 133.3, 131.8, 131.1, 130.4, 128.8, 128.7, 128.0, 127.5, 123.3, 119.1, 115.3, 115.2, 111.8, 80.5, 70.1, 56.3, 53.8, 52.4, 51.1, 41.3, 37.1, 30.9, 28.3, 24.8, 22.9, 22.0. IR (cm^{-1}): 3291, 1742, 1685, 1642, 1549, 1238. HRMS (ESI) m/z: (M^+) *calcd* for ($C_{44}H_{52}N_4O_9$): 780.3734, *found* 780.3747.

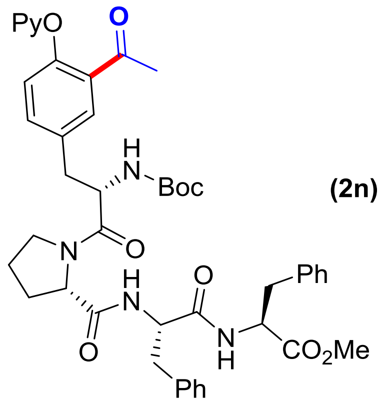

(2n)

Methyl [(*S*)-3-(3-acetyl-4-(pyridin-2-yloxy)phenyl)-2-((*tert*-butoxycarbonyl)amino) propanoyl]-*L*-prolyl-*L*-phenylalanyl-*L*-phenylalaninate (2n). Following the general procedure A, using Boc-Tyr(OPy)-Pro-Phe-Phe-OMe[1] **(1n)** (0.15 mmol, 114 mg) provided 65 mg of **2n** (54% yield) as a white solid. Column chromatography (EtOAc). Mp 59-60 ºC. ^{1}H NMR (500 MHz, DMSO-d_6 at 80 ºC) δ 8.10 (dd, J = 5.0, 2.0 Hz, 1H), 7.96 (d, J = 7.6 Hz, 1H), 7.91 – 7.69 (m, 1H), 7.68 – 7.53 (m, 2H), 7.46 (d, J = 8.3 Hz, 1H), 7.31 – 7.14 (m, 8H), 7.10 (dd, J = 7.2, 4.9 Hz, 1H), 7.02 (t, J = 8.7 Hz, 1H), 4.63 – 4.26 (m, 4H), 3.58 (s, 3H), 2.96 – 2.74 (m, 1H), 2.41 (s, 2H), 2.03 – 1.65 (m, 3H), 1.32 (s, 9H). ^{13}C NMR (126 MHz, DMSO-d_6 at 80 ºC) 197.3, 170.9, 170.5, 170.2, 169.9, 162.4, 150.3, 146.9, 139.6, 137.2, 136.6, 134.0, 133.7, 131.3, 129.8, 128.6, 128.5, 127.7, 127.5, 127.4, 126.0, 125.7, 122.2, 118.6, 111.0, 77.9, 59.4, 54.2, 53.2, 51.2, 46.3, 36.9, 36.6, 35.8, 29.6, 27.7. IR (cm^{-1}): 3294, 1741, 1637, 1427, 1239, 1047, 699. HRMS (ESI) m/z: (M$^+$) *calcd* for ($C_{45}H_{51}N_5O_9$): 805.3687, *found* 805.3696.

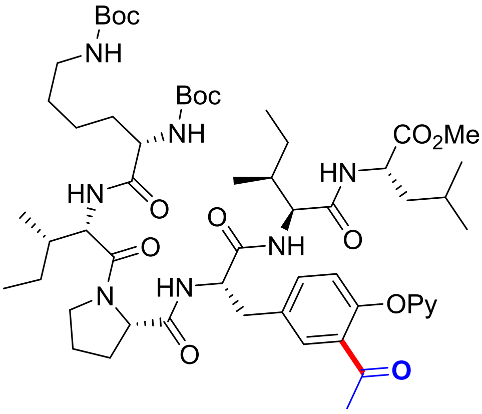

(2o)

Methyl [(*S*)-3-(3-acetyl-4-(pyridin-2-yloxy)phenyl)-2-((*S*)-1-(N^2,N^6-bis(*tert*-butoxy-carbonyl)-*L*-lysyl-*L*-isoleucyl)pyrrolidine-2-carboxamido)propanoyl)-*L*-isoleucyl-*L*-leucinate (2o). Following the general procedure A, using Boc-Lys(Boc)-Ile-Pro-Tyr(OPy)-Ile-Leu-OMe[1] **(1o)** (0.15 mmol, 155 mg) provided 87.3 mg of **2o** (54% yield) as a white solid. Column chromatography (EtOAc). Mp 73-74 ºC. ^{1}H NMR (500MHz,

DMSO-d_6) δ 8.12 (dd, J = 5.0, 1.9 Hz, 1H), 7.95 (d, J = 7.6 Hz, 2H), 7.92 – 7.80 (m, 1H), 7.79 – 7.36 (m, 7H), 7.25 (d, J = 8.2 Hz, 1H), 7.11 (dd, J = 7.2, 4.8 Hz, 1H), 7.07 – 6.90 (m, 3H), 6.52 (s, 1H), 6.30 (s, 1H), 4.61 (td, J = 8.0, 5.2 Hz, 2H), 4.51 – 4.30 (m, 5H), 4.26 (dd, J = 8.8, 6.9 Hz, 2H), 3.91 (q, J = 7.5 Hz, 2H), 3.70 (s, 1H), 3.61 (s, 5H), 3.53 (s, 2H), 3.12 (dd, J = 14.4, 5.2 Hz, 2H), 3.00 – 2.79 (m, 5H), 2.43 (s, 3H), 1.98 (s, 2H), 1.90 – 1.70 (m, 8H), 1.69 – 1.45 (m, 12H), 1.38 (d, J = 1.7 Hz, 36H), 1.27 (tq, J = 15.8, 9.4, 6.3 Hz, 5H), 1.20 – 1.02 (m, 4H), 0.85 (ddt, J = 17.5, 14.4, 7.2 Hz, 33H). ^{13}C NMR (126 MHz, DMSO-d_6 at 80 °C) δ 197.2, 172.0, 171.4, 170.8, 170.3, 170.3, 169.9, 169.8, 169.7, 162.4, 155.1, 154.7, 152.3, 150.3, 147.0, 146.9, 139.6, 139.4, 133.9, 133.4, 131.2, 129.8, 129.7, 122.0, 119.8, 118.6, 118.4, 111.0, 77.8, 77.0, 59.1, 56.4, 54.4, 54.0, 53.2, 51.0, 50.0, 46.6, 36.7, 36.1, 31.1, 29.6, 28.8, 27.9, 27.7, 23.9, 23.8, 23.6, 22.3, 22.1, 21.0, 14.7, 10.5. IR (cm^{-1}): 3285, 1746, 1682, 1643, 1465, 1243, 1165. HRMS (ESI) m/z: (M$^+$) *calcd* for ($C_{56}H_{86}N_8O_{13}$): 1078.6314, *found* 1078.6312.

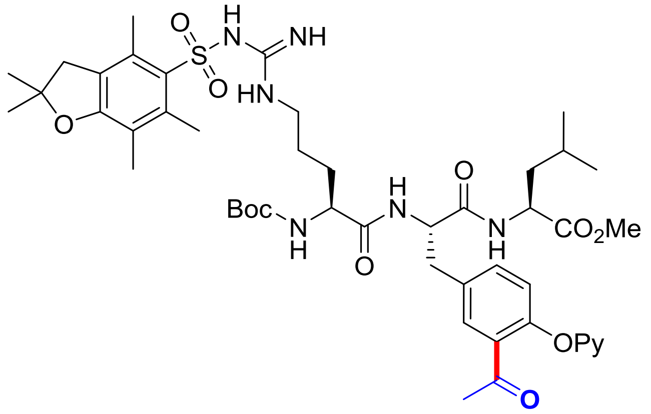

(2p)

Methyl [(*S*)-3-(3-acetyl-4-(pyridin-2-yloxy)phenyl)-2-((*S*)-2-((*tert*-butoxycarbonyl) amino)-5-(3-((2,2,4,6,7-pentamethyl-2,3-dihydrobenzofuran-5-yl)sulfonyl) guanidino)pentanamido)propanoyl]-*L*-leucinate (2p). Following the general procedure A, *in PhCl as solvent* using Boc-Arg(Pbf)-Tyr(OPy)-Leu-OMe **(1p)** (0.15 mmol, 134 mg) provided 56 mg of **2p** (40% yield) as a colorless oil Column chromatography (EtOAc). ^{1}H NMR (300MHz, $CDCl_3$) δ 8.10 (dd, J = 5.0, 1.9 Hz, 1H), 7.80 – 7.59 (m, 2H), 7.52 – 7.32 (m, 2H), 7.09 – 6.86 (m, 4H), 6.32 (s, 2H), 5.55 (d, J = 7.8 Hz, 1H), 4.77 – 4.69 (m, 1H), 4.57 – 4.37 (m, 1H), 4.19 – 4.15 (m, 1H), 3.66 (s, 3H), 3.34 – 2.98 (m, 5H), 2.94 (s, 2H), 2.57 (s, 3H), 2.49 (d, J = 9.6 Hz, 5H), 2.41 (s, 1H), 2.24 (s, 2H), 2.07 (s, 3H), 1.71 – 1.48 (m, 7H), 1.44 (s, 6H), 1.37 (s, 9H), 0.85 (d, J = 3.8 Hz, 6H). ^{13}C NMR (75 MHz, $CDCl_3$) δ 199.3, 173.1, 172.7, 171.0, 163.1, 158.8, 156.6, 156.0, 151.7, 147.7, 140.0, 138.5, 134.7, 134.2, 134.1, 133.8, 133.0, 132.4, 131.6, 131.2, 131.0, 130.8, 124.7, 123.2, 121.2, 119.1, 117.6, 111.9, 86.5, 80.1, 54.6, 54.2, 52.4, 51.1, 43.4,

41.0, 40.4, 36.9, 30.9, 28.7, 28.4, 24.8, 22.8, 21.9, 19.5, 18.1, 12.6. IR (cm^{-1}): 3363, 1738, 1716, 1675, 1667, 1240, HRMS (ESI) m/z: (M^+) *calcd* for ($C_{47}H_{65}N_7O_{11}S$): 935.4463, *found* 935.4479.

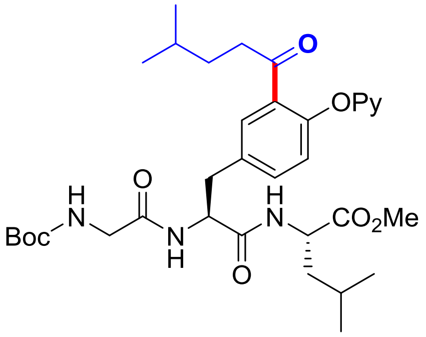

(2q)

Methyl [(*S*)-2-(2-((*tert*-butoxycarbonyl)amino)acetamido]-3-[3-(4-methylpentanoyl) -4-(pyridin-2-yloxy)phenyl)propanoyl]-*L*-leucinate (2q). Following the general procedure B, using **1q** (0.15 mmol, 79 mg) and 4-methylpentan-1-ol (0.75 mmol, 93 μL) provided 38 mg (50% yield) of **2q** as a yellow oil. Column chromatography (Hex/EtOAc 2:3). ^{1}H NMR (400 MHz, $CDCl_3$) δ 8.13 (d, *J* = 5.0 Hz, 1H), 7.72 (t, *J* = 7.1 Hz, 1H), 7.55 (d, *J* = 2.0 Hz, 1H), 7.37 (d, *J* = 8.1 Hz, 1H), 7.02 (dd, *J* = 12.4, 7.4 Hz, 2H), 6.96 (d, *J* = 8.2 Hz, 1H), 6.76 (s, 1H), 6.52 (s, 1H), 5.35 (s, 1H), 4.80 – 4.63 (m, 1H), 4.59 – 4.46 (m, 1H), 3.86 – 3.72 (m, 2H), 3.71 (s, 3H), 3.20 – 3.07 (m, 2H), 2.88 – 2.79 (m, 2H), 1.65 – 1.50 (m, 3H), 1.48 – 1.38 (m, 12H), 0.89 (d, *J* = 5.5 Hz, 6H), 0.78 (d, *J* = 5.8 Hz, 6H). ^{13}C NMR (101 MHz, $CDCl_3$) δ 201.7, 173.1, 171.8, 171.0, 163.1, 155.3, 151.2, 147.6, 139.7, 133.7, 133.3, 132.4, 130.7, 123.3, 118.8, 111.6, 80.4, 55.5, 52.6, 51.8, 51.7, 40.8, 37.3, 32.8, 29.8, 28.2, 27.7, 27.2, 27.0, 22.3. IR (cm^{-1}): 3289, 2956, 1647, 1241, 1164. HRMS (ESI) m/z: (M^+) *calcd* for ($C_{34}H_{48}N_4O_8$): 640.3472, *found* 640.3476.

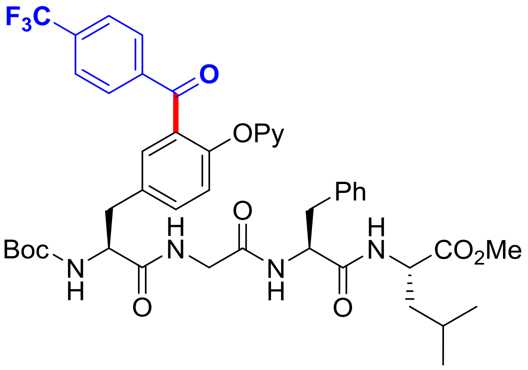

(2r)

Methyl [(*S*)-2-((*tert*-butoxycarbonyl)amino)-3-(4-(pyridin-2-yloxy)-3-(4-(trifluoromethyl)benzoyl)phenyl)propanoyl]glycyl-*L*-phenylalanyl-*L*-leucinate (2r). Following the general procedure C, using **1r** (0.15 mmol, 103 mg) and (4-(trifluoromethyl)phenyl)methanol (0.45 mmol, 63 μL) provided 63 mg (50% yield) of **2r**

as a white solid. Mp 95-96 ºC. Column chromatography (EtOAc). ^{1}H NMR (300 MHz, $CDCl_3$) δ 7.93 (d, *J* = 5.0 Hz, 1H), 7.77 (d, *J* = 8.1 Hz, 2H), 7.52 (d, *J* = 8.2 Hz, 2H), 7.48 – 7.39 (m, 3H), 7.30 – 7.12 (m, 7H), 7.02 (d, *J* = 8.0 Hz, 1H), 6.86 (dd, *J* = 6.9, 5.2 Hz, 1H), 6.80 (d, *J* = 8.0 Hz, 1H), 6.46 (d, *J* = 8.3 Hz, 1H), 5.54 (d, *J* = 7.3 Hz, 1H), 4.76 (dd, *J* = 6.9 Hz, 1H), 4.60 – 4.42 (m, 2H), 3.98 (dd, *J* = 16.6, 5.6 Hz, 1H), 3.80 (dd, *J* = 16.6, 5.1 Hz, 1H), 3.67 (s, 3H), 3.19 (dd, *J* = 13.9, 5.6 Hz, 1H), 3.13 – 2.93 (m, 3H), 1.61 – 1.42 (m, 3H), 1.36 (s, 9H), 0.85 (d, *J* = 5.2 Hz, 6H). ^{13}C NMR (75 MHz, $CDCl_3$) δ 194.2, 173.0, 171.9, 170.6, 168.6, 162.5, 155.6, 150.5, 146.7, 140.5, 139.4, 136.3, 134.0, 133.8, 133.7 (q, J_{C-F} = 32.5 Hz), 131.2, 131.1, 129.7, 129.3, 128.5, 126.9, 124.9 (q, J_{C-F} = 3.8 Hz), 123.5 (q, J_{C-F} = 270.0 Hz), 123.0, 118.6, 111.2, 80.2, 55.4, 54.4, 52.2, 50.8, 43.0, 41.1, 38.2, 37.7, 28.2, 24.7, 22.6, 21.8. IR (cm^{-1}): 3294, 1641, 1242, 1165. HRMS (ESI) m/z: (M^+) *calcd* for ($C_{45}H_{50}F_3N_5O_9$): 861.3561, *found* 861.3572.

5.- Control Experiments and Mechanism Proposal

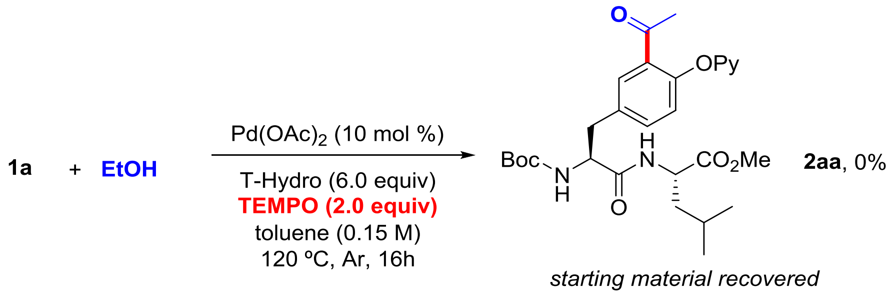

Table S4. Control Experiments with MeCHO[a]

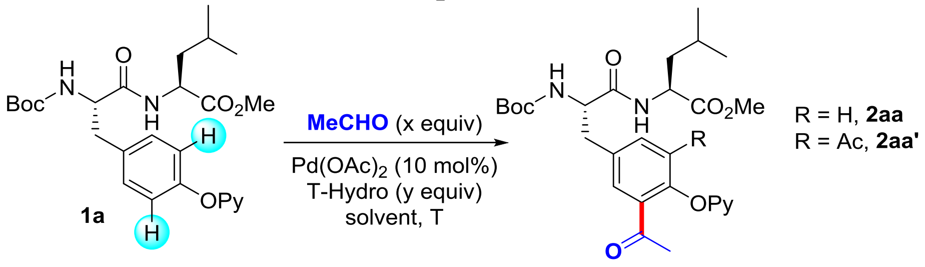

entry	MeCHO	T-Hydro	Solvent & T	**2aa:2aa'** (%)[b]
1	4	4	water at 90 ºC	traces
2	4	4	PhMe at 90 ºC	22:61
3	25	6	PhMe at 120 ºC	0:62

[a]Reaction conditions: **1a** (0.15 mmol), MeCHO (x mmol), T-Hydro (y mmol), toluene (1.0 mL) under Ar for 16h. [b] Yield of isolated product after column chromatography.

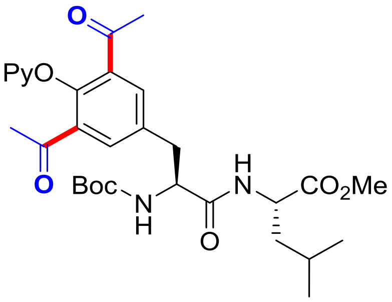

(2aa')

Methyl [(*S*)-2-((*tert*-butoxycarbonyl)amino)-3-(3,5-diacetyl-4-(pyridin-2-yloxy) phenyl)propanoyl]-*L*-leucinate (2aa'). Following the general procedure A, using **1a** (0.15 mmol, 73 mg) and MeCHO (3.75 mmol, 0.21 mL) provided 53 mg (62% yield) of **2aa'** as a white solid. Mp 90-91 ºC. Column chromatography (Hex/EtOAc 1:1). ^{1}H NMR (400 MHz, $CDCl_3$) δ 8.01 (dd, J = 5.1, 1.9 Hz, 1H), 7.88 – 7.62 (m, 3H), 7.09 – 6.91 (m, 2H), 6.71 (d, J = 7.8 Hz, 1H), 5.33 (d, J = 8.3 Hz, 1H), 4.61 – 4.45 (m, 1H), 4.48 – 4.43 (m, 1H), 3.70 (s, 3H), 3.23 (dd, J = 14.1, 6.3 Hz, 1H), 3.07 (dd, J = 14.0, 7.0 Hz, 1H), 2.44 (s, 6H), 1.72 – 1.50 (m, 3H), 1.41 (s, 9H), 0.90 (dd, J = 6.0, 3.8 Hz, 6H). ^{13}C NMR (101 MHz, $CDCl_3$) δ 198.5, 173.0, 170.7, 162.8, 155.4, 148.5, 147.3, 140.1, 134.3, 134.2, 134.0, 118.9, 111.3, 80.3, 55.1, 52.4, 50.8, 41.3, 37.3, 30.4, 28.3, 24.7, 22.8, 21.8. IR (cm^{-}

[1]): 3326, 1757, 1741, 1686, 1656, 1520, 1444, 1207. HRMS (ESI) m/z: (M^{+}) *calcd* for ($C_{30}H_{39}N_3O_8$): 569.2737, *found* 569.2741.

The accepted mechanism for simple phenyl systems starts with the coordination of the OPy group with the Pd catalyst and further *ortho*-palladation delivers 6-membered palladacycle **I**. The latter, which is often proposed to exist as a dimeric species, could undergo dissociation and further addition of the *in situ* formed acyl radical species from consecutive oxidation events from EtOH, thereby resulting in the formation of a transient Pd(III) species. Although *in-depth* studies are arguably required, intermediate **II** would likely evolve into the corresponding Pd(IV) intermediate (**III**) in the oxidizing reaction conditions. Eventually, reductive elimination would deliver the acetylated peptide and the active Pd(II) catalyst.

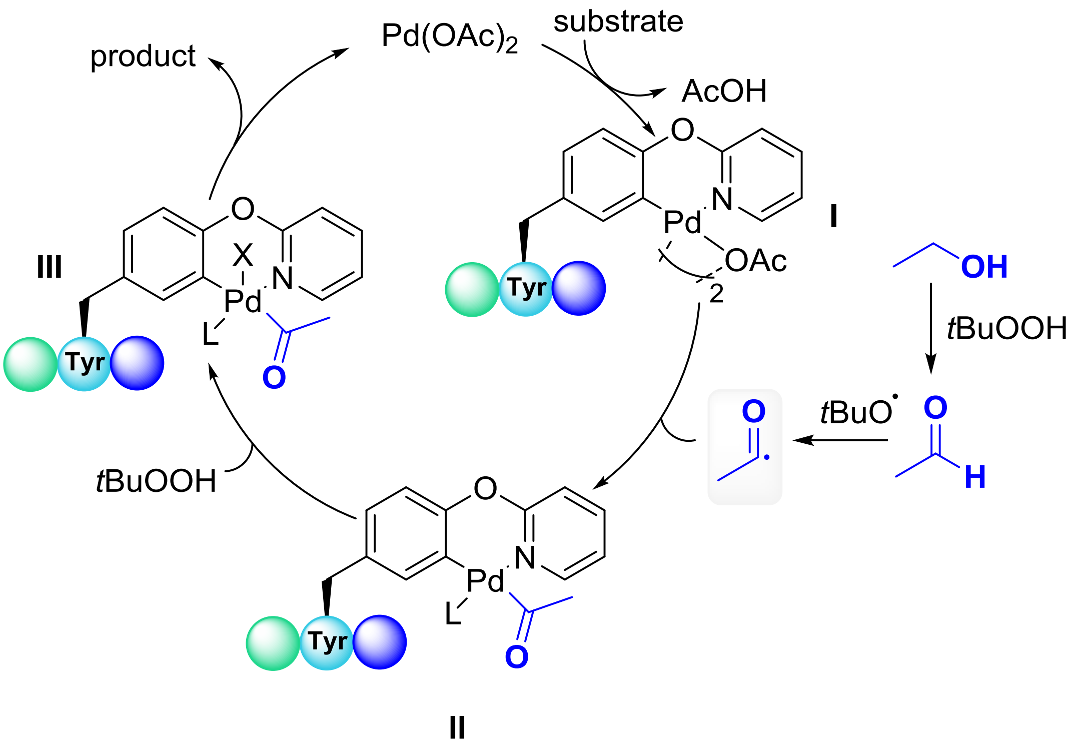

6.- ^{1}H NMR and ^{13}C NMR Spectra

^{1}H NMR (400 MHz, $CDCl_3$)

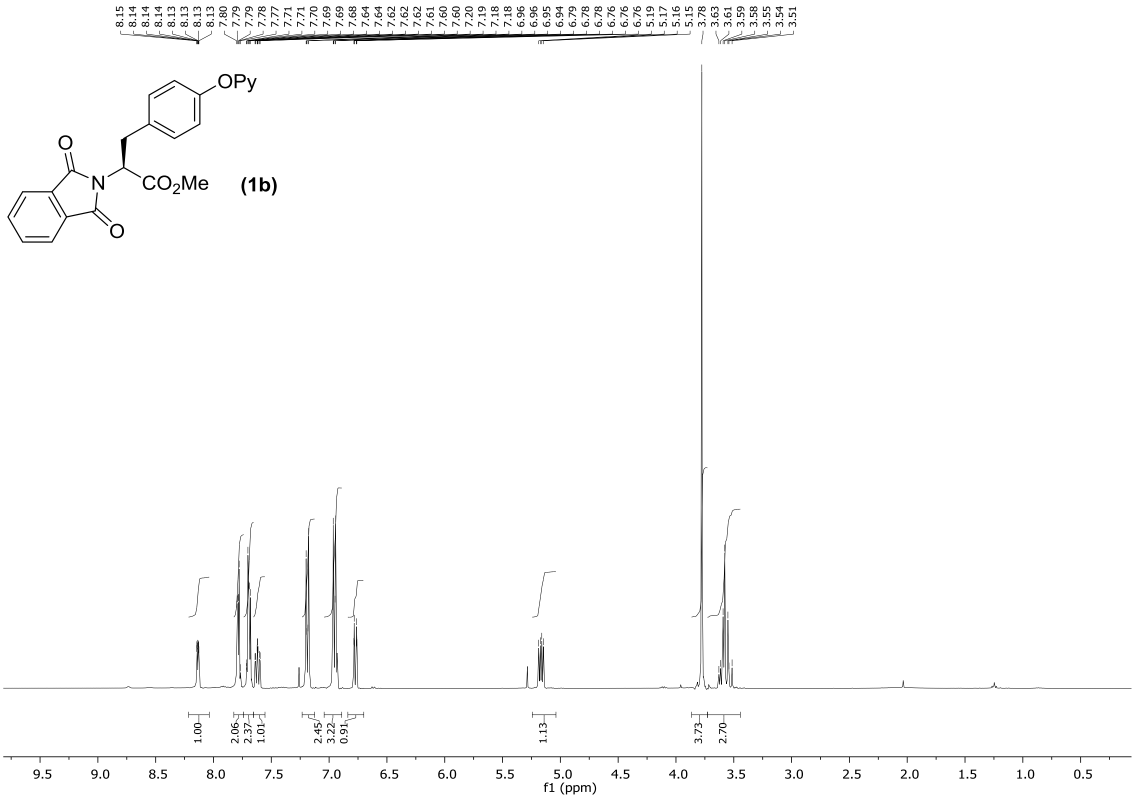

^{13}C NMR (101 MHz, $CDCl_3$)

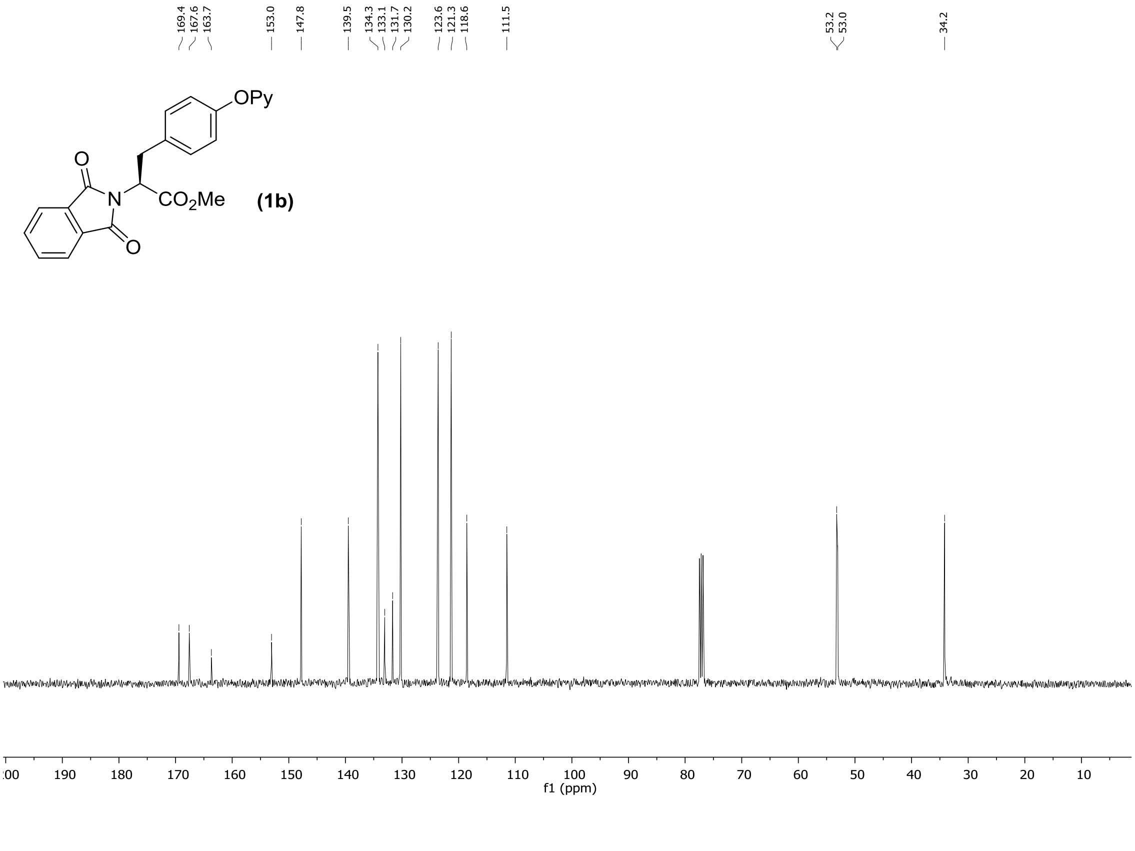

^{1}H NMR (300 MHz, $CDCl_3$)

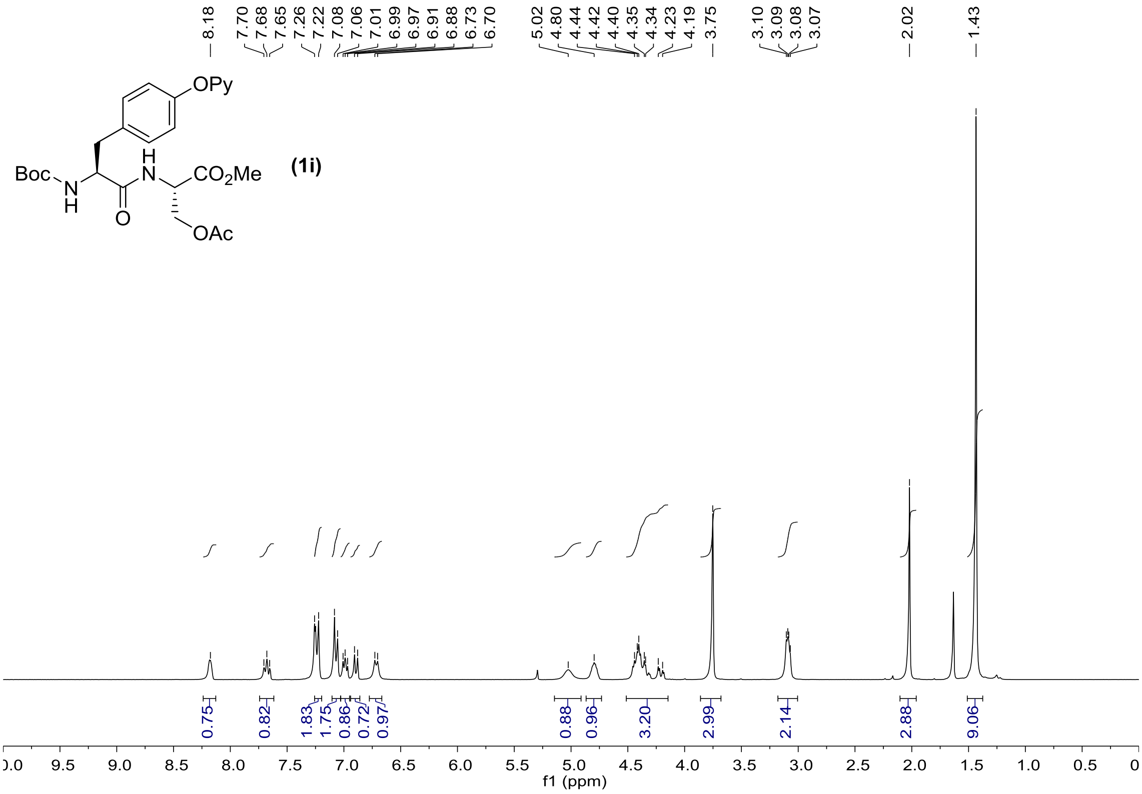

^{13}C NMR (75 MHz, $CDCl_3$)

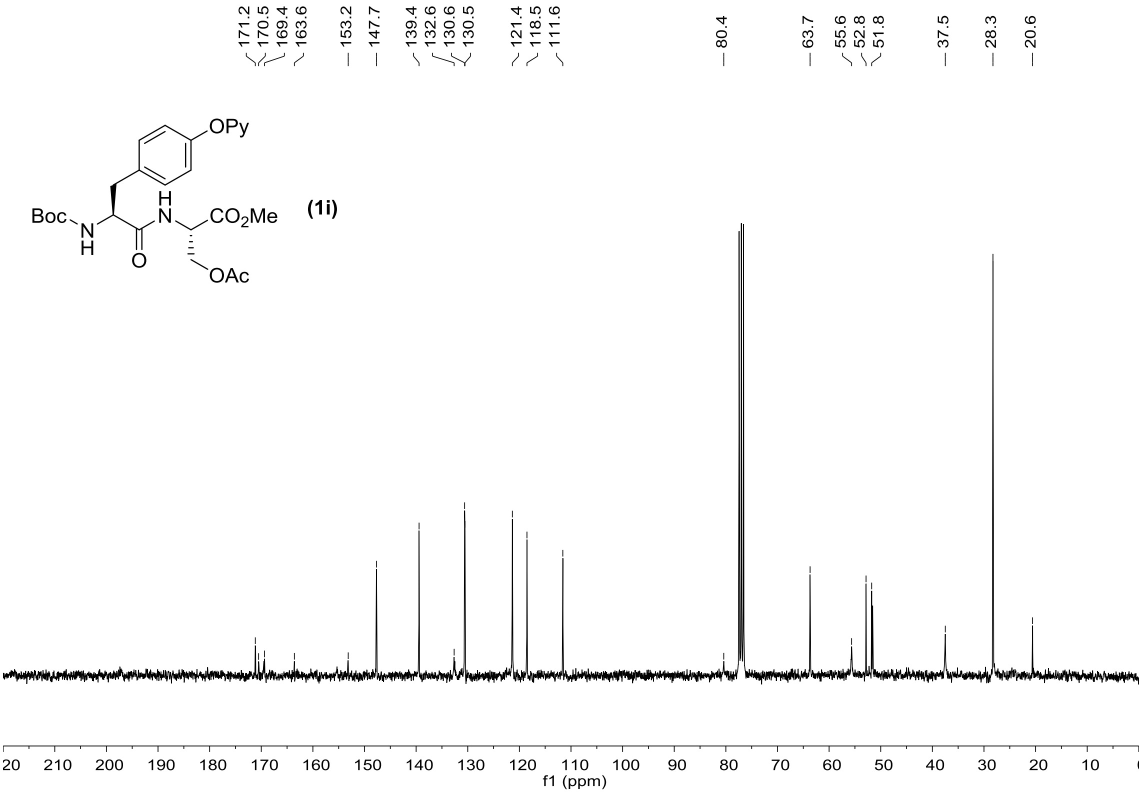

^{1}H NMR (300 MHz, $CDCl_3$)

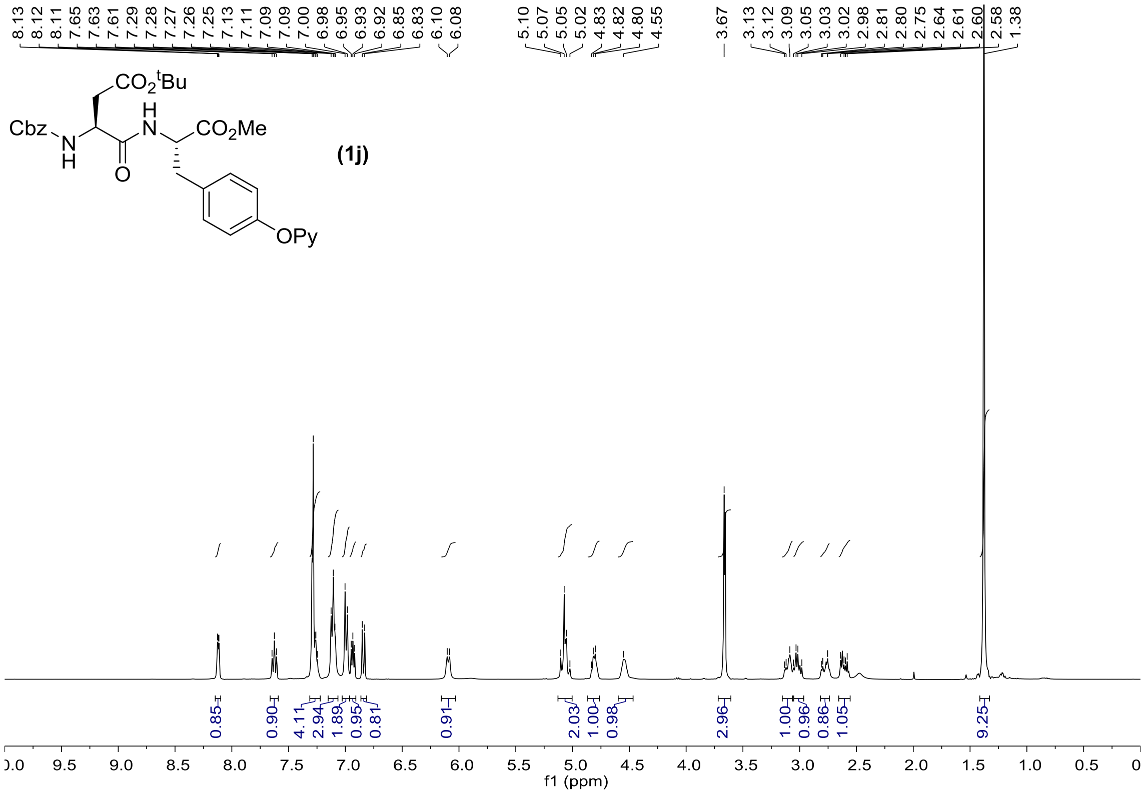

^{13}C NMR (75 MHz, $CDCl_3$)

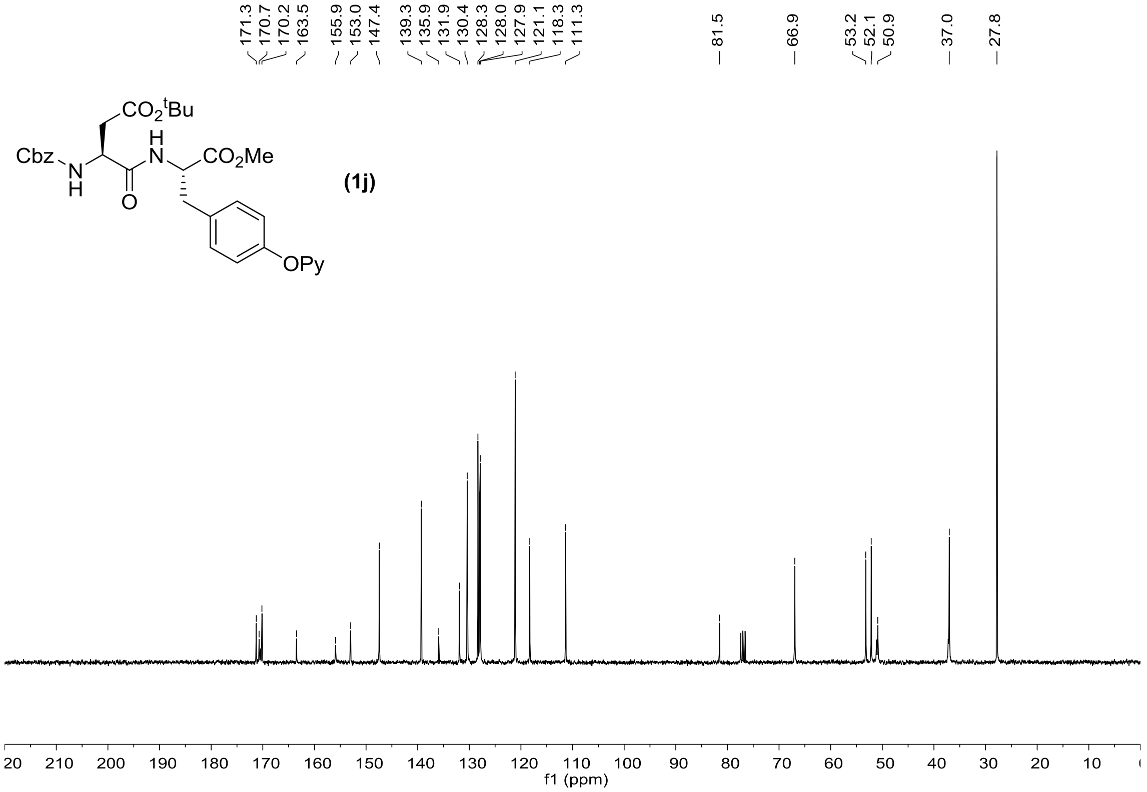

^{1}H NMR (300 MHz, $CDCl_3$)

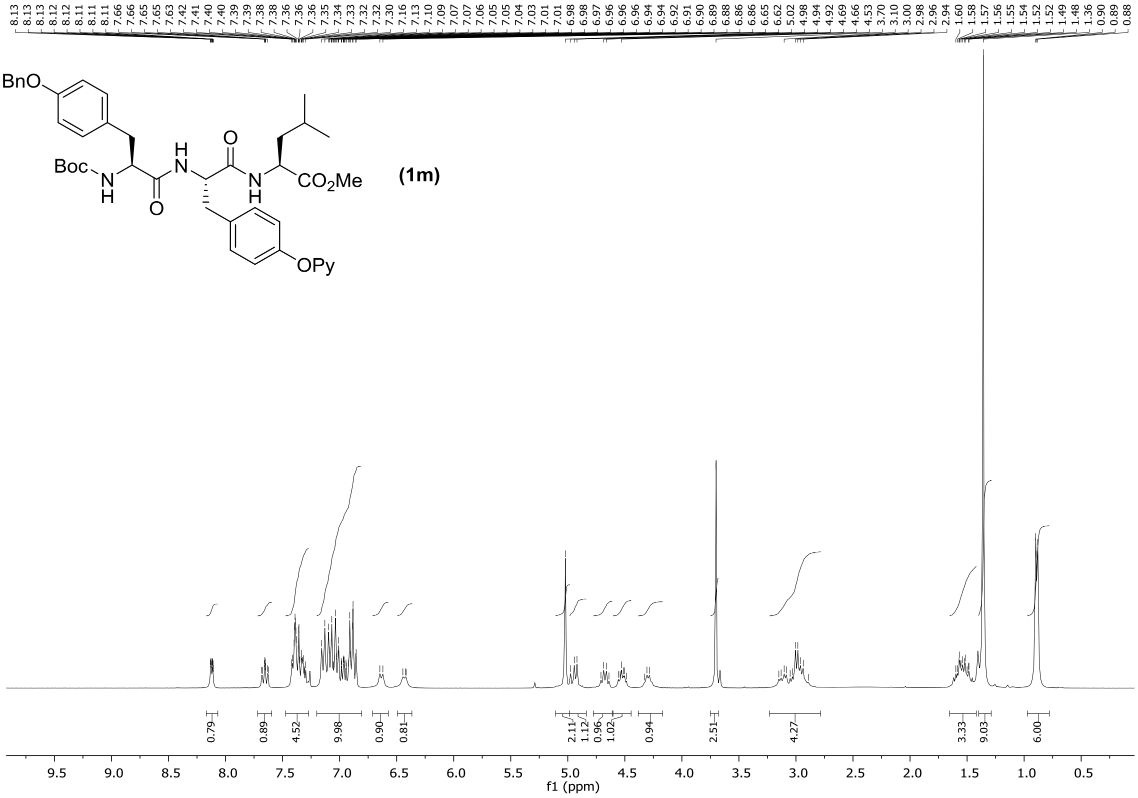

^{13}C NMR (75 MHz, $CDCl_3$)

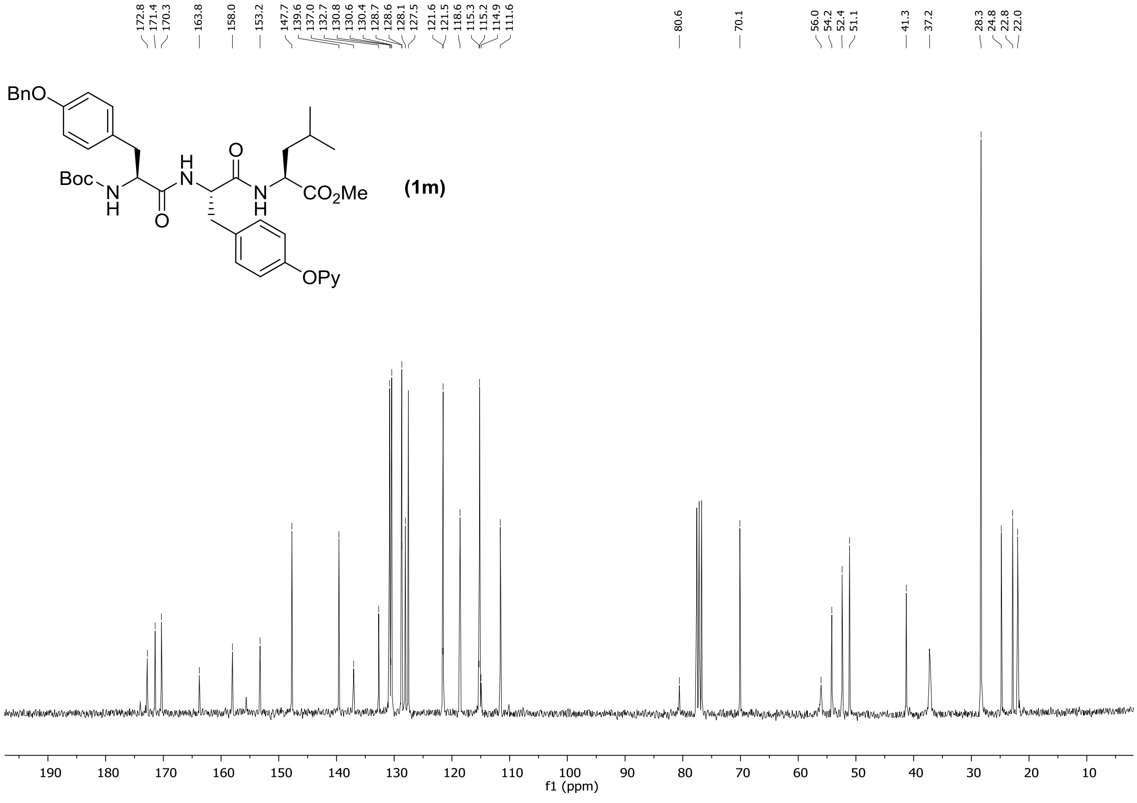

^{1}H NMR (300 MHz, $CDCl_3$)

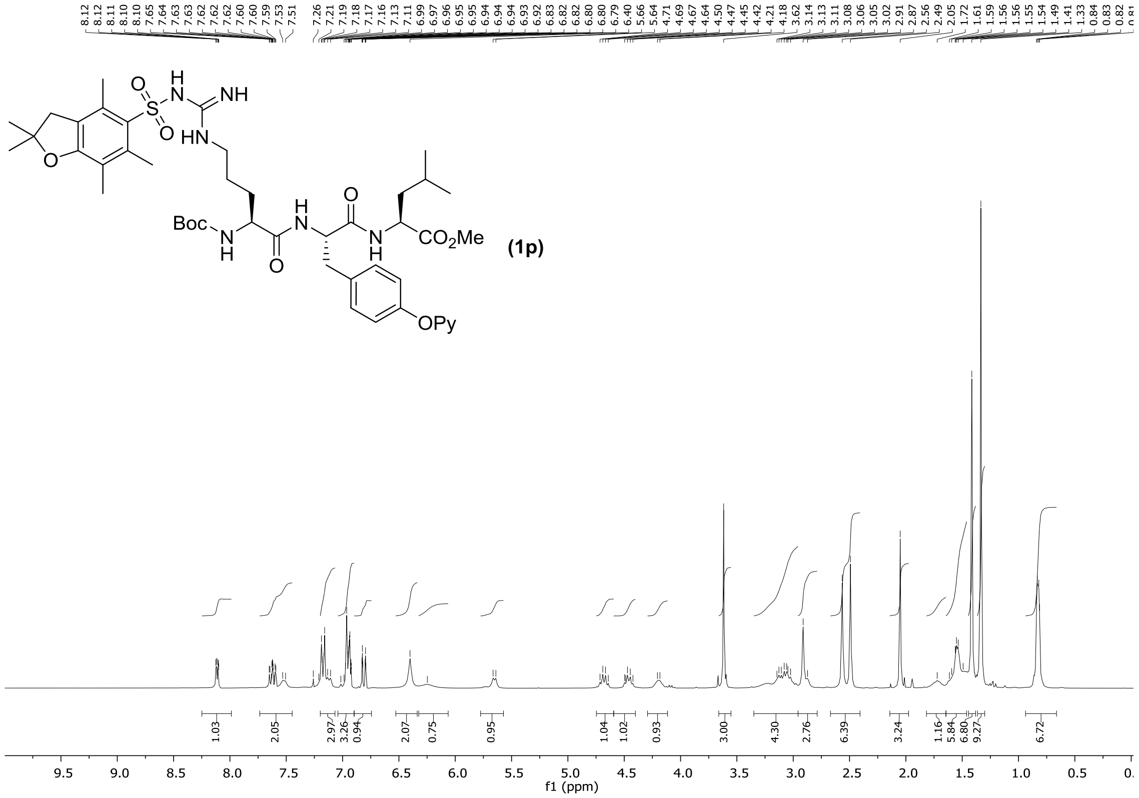

^{13}C NMR (75 MHz, $CDCl_3$)

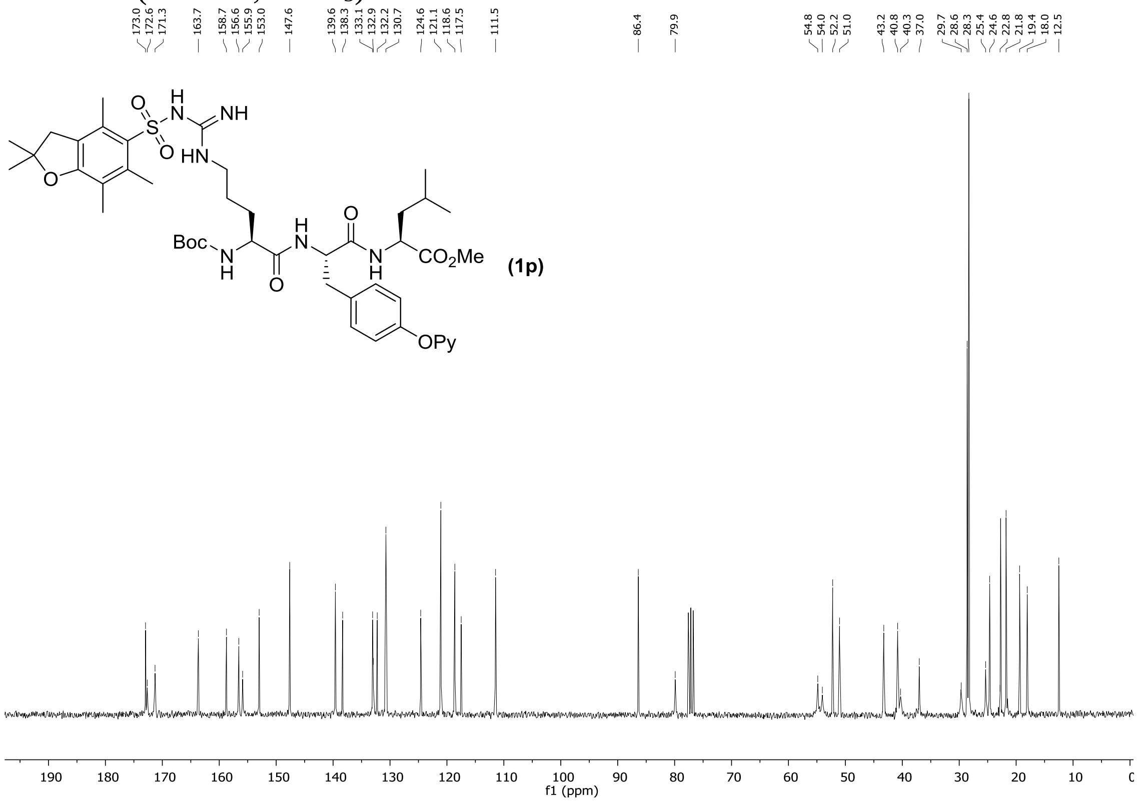

^{1}H NMR (400 MHz, $CDCl_3$)

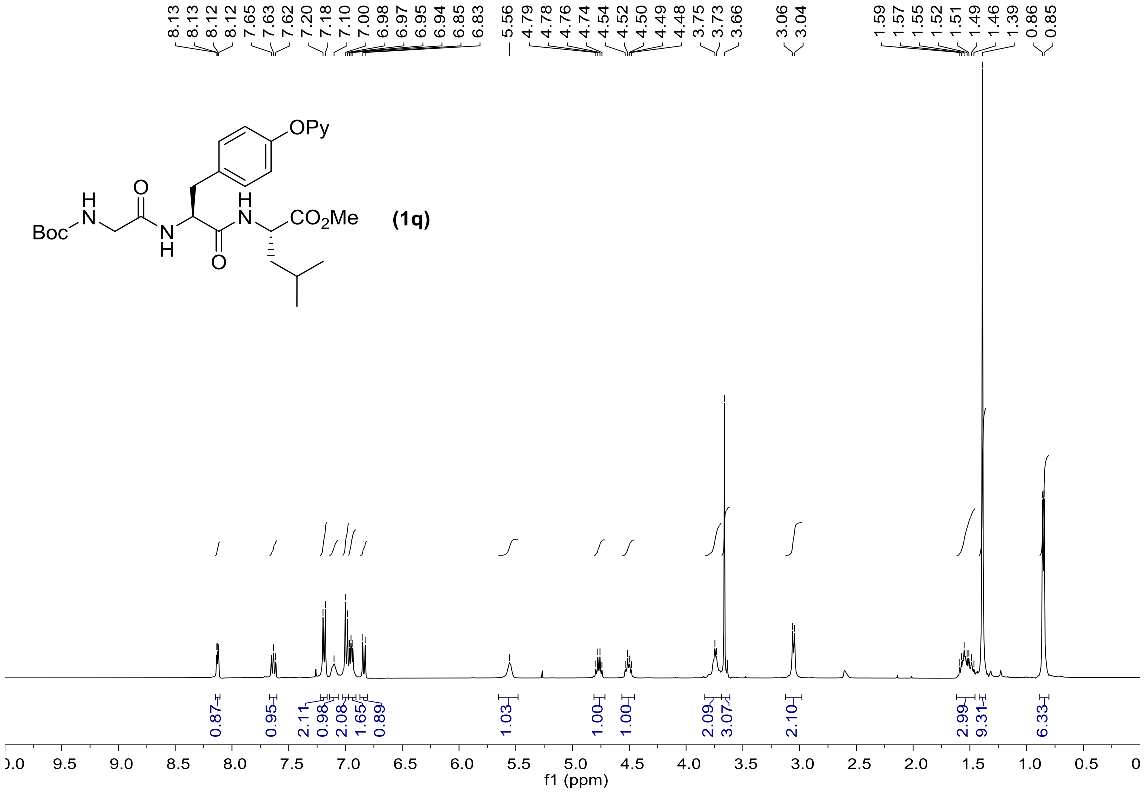

^{13}C NMR (101 MHz, $CDCl_3$)

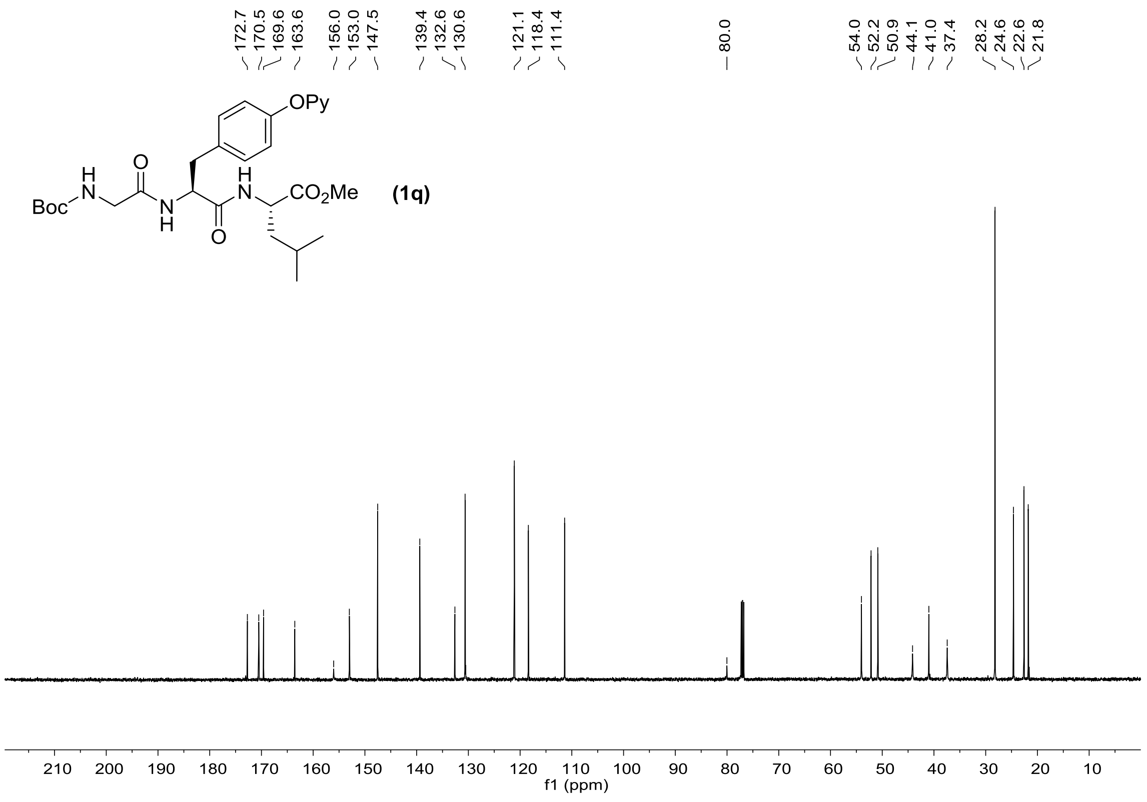

^{1}H NMR (300 MHz, $CDCl_3$)

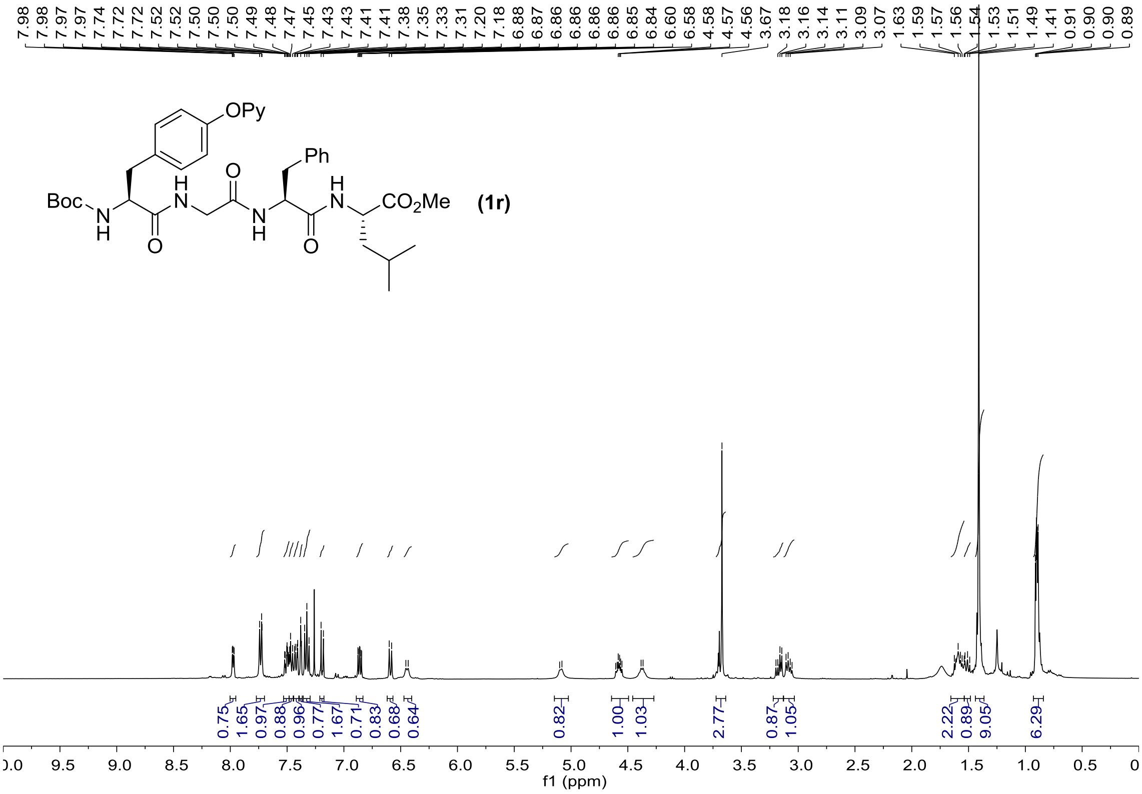

^{13}C NMR (75 MHz, $CDCl_3$)

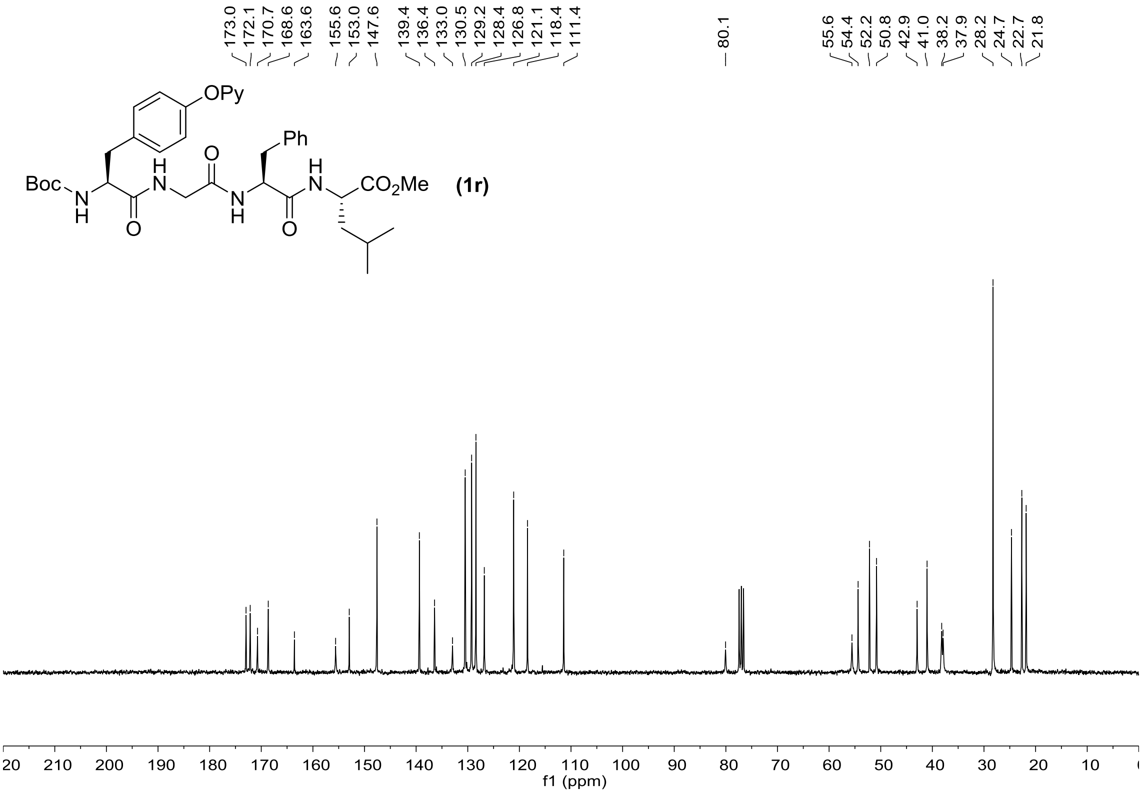

^{1}H NMR (400 MHz, $CDCl_3$)

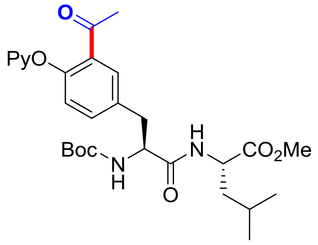

(2aa)

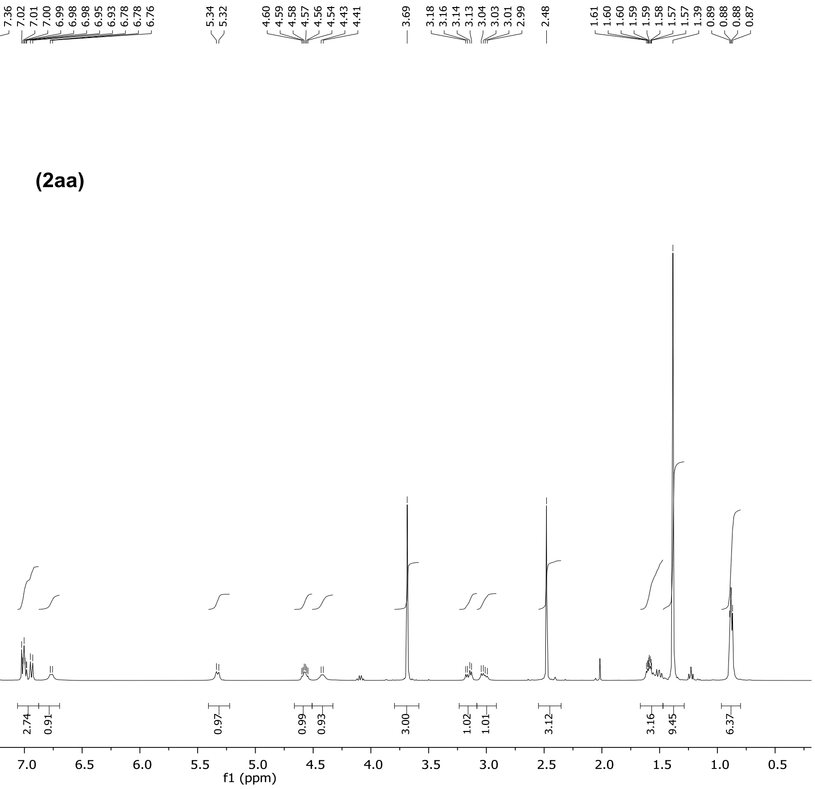

^{13}C NMR (101 MHz, $CDCl_3$)

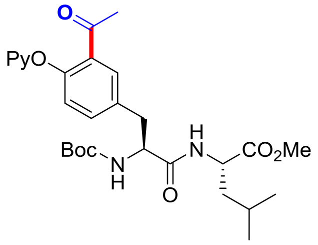

(2aa)

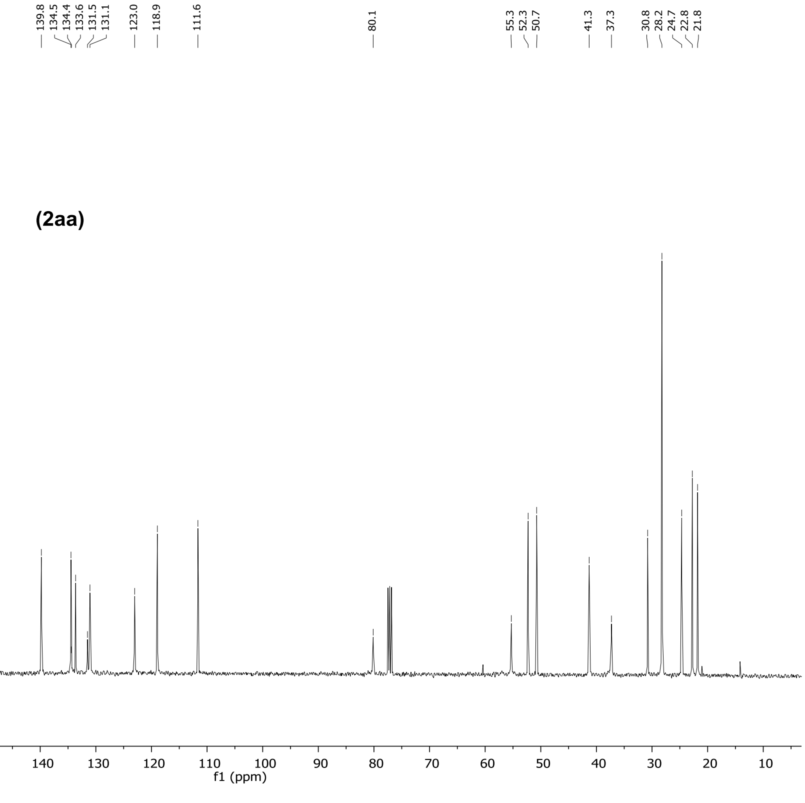

^{1}H NMR (400 MHz, $CDCl_3$)

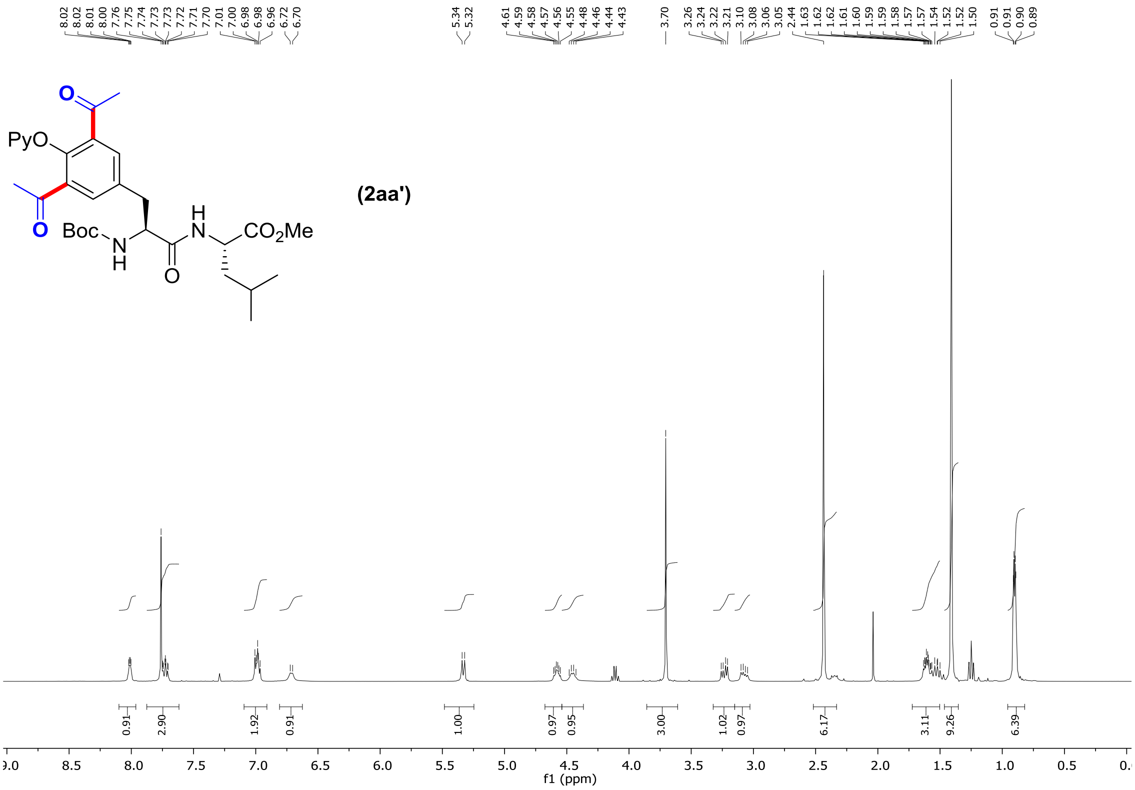

^{13}C NMR (101 MHz, $CDCl_3$)

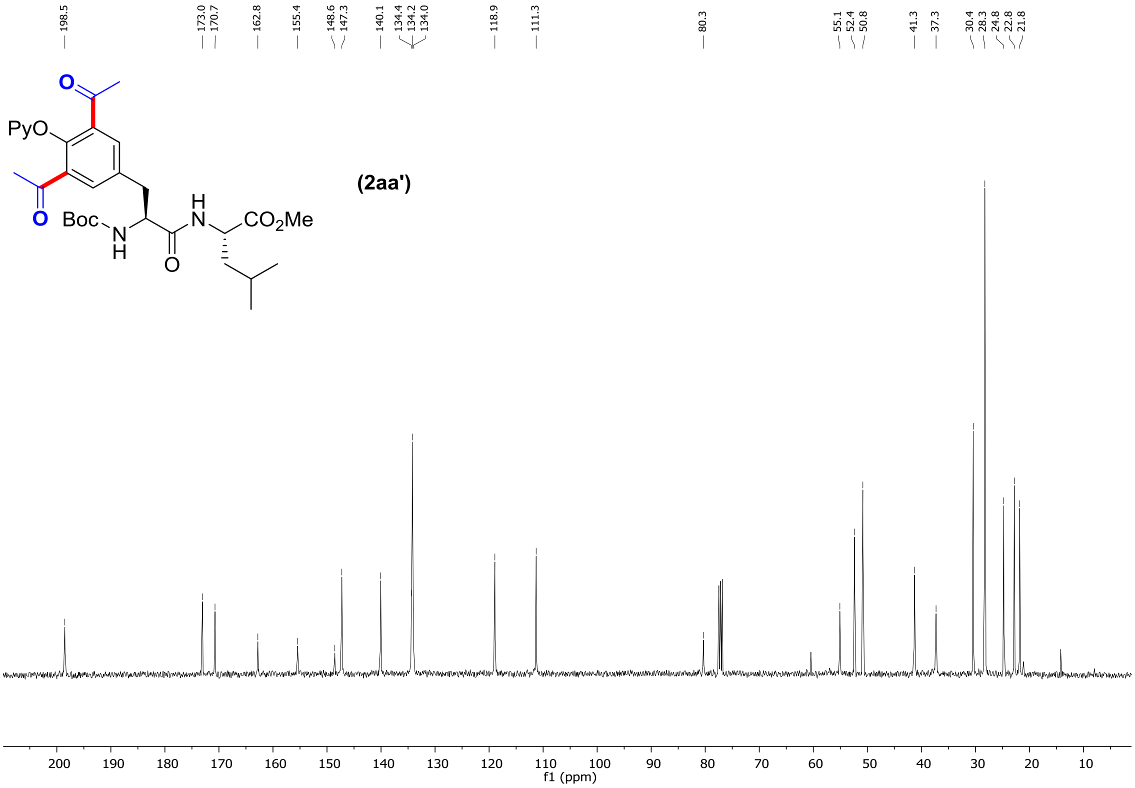

^{1}H NMR (400 MHz, $CDCl_3$)

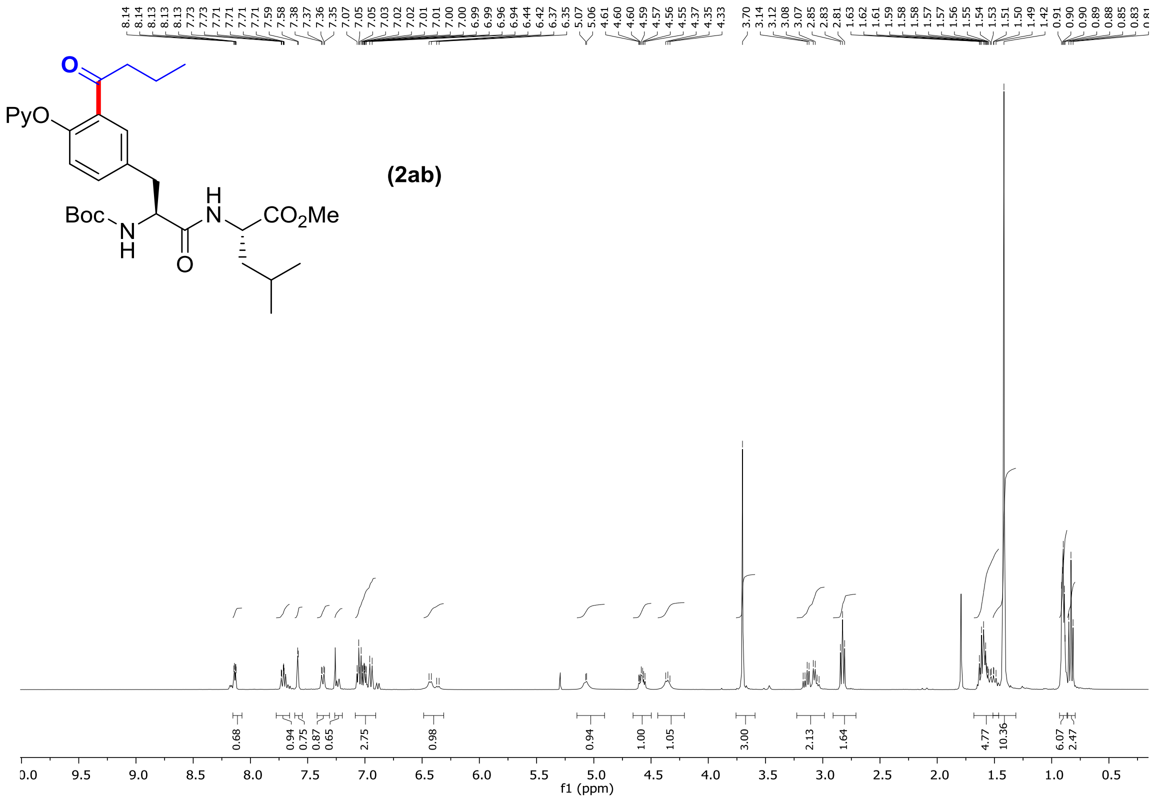

^{13}C NMR (75 MHz, $CDCl_3$)

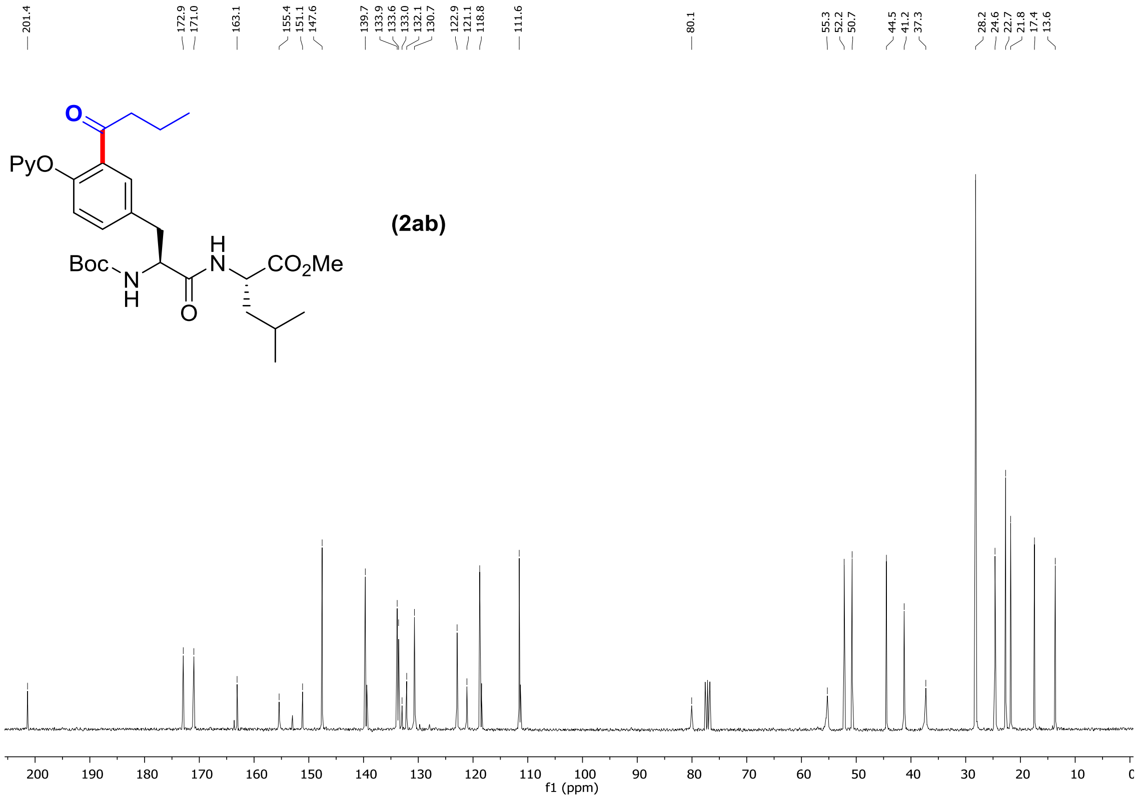

^{1}H NMR (400 MHz, $CDCl_3$)

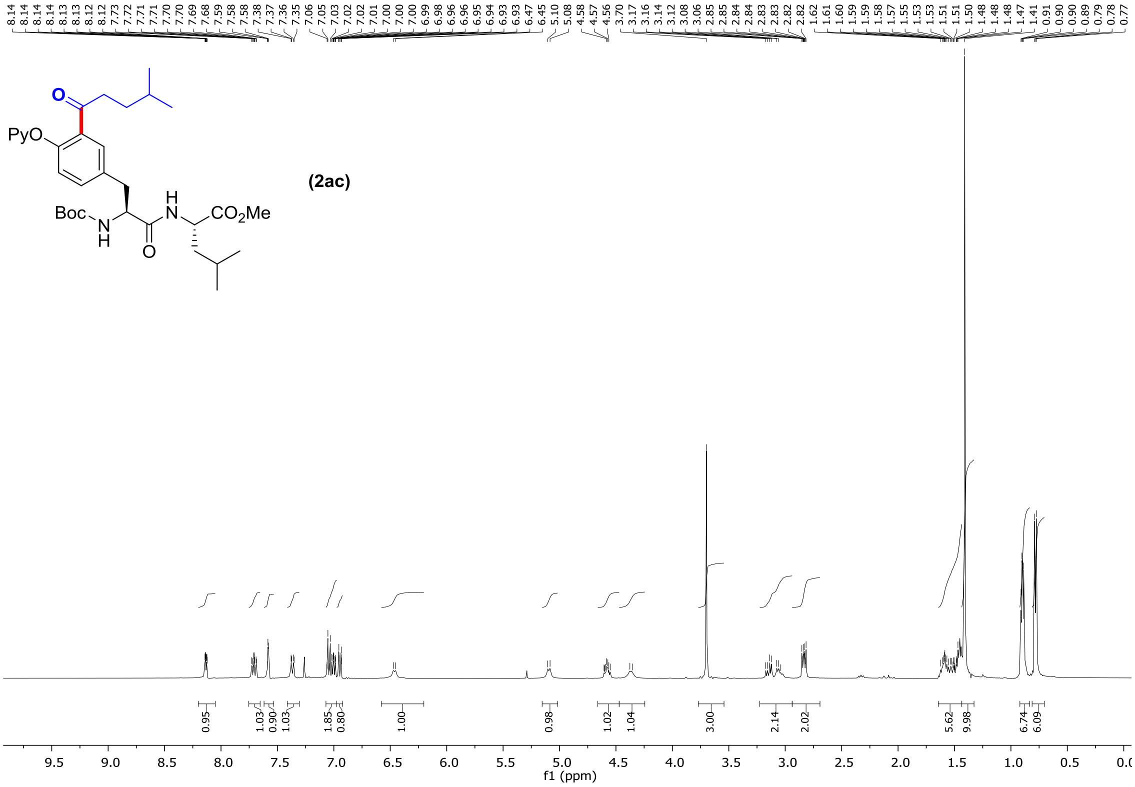

^{13}C NMR (101 MHz, $CDCl_3$)

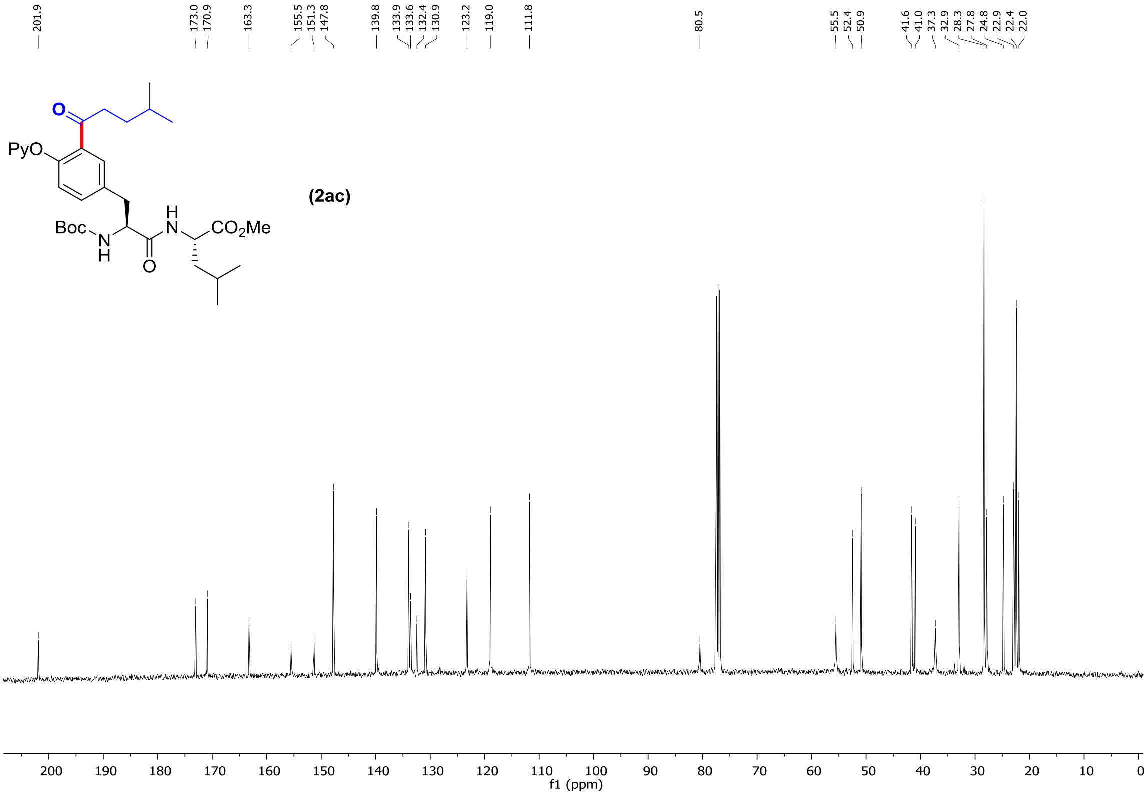

^{1}H NMR (400 MHz, $CDCl_3$)

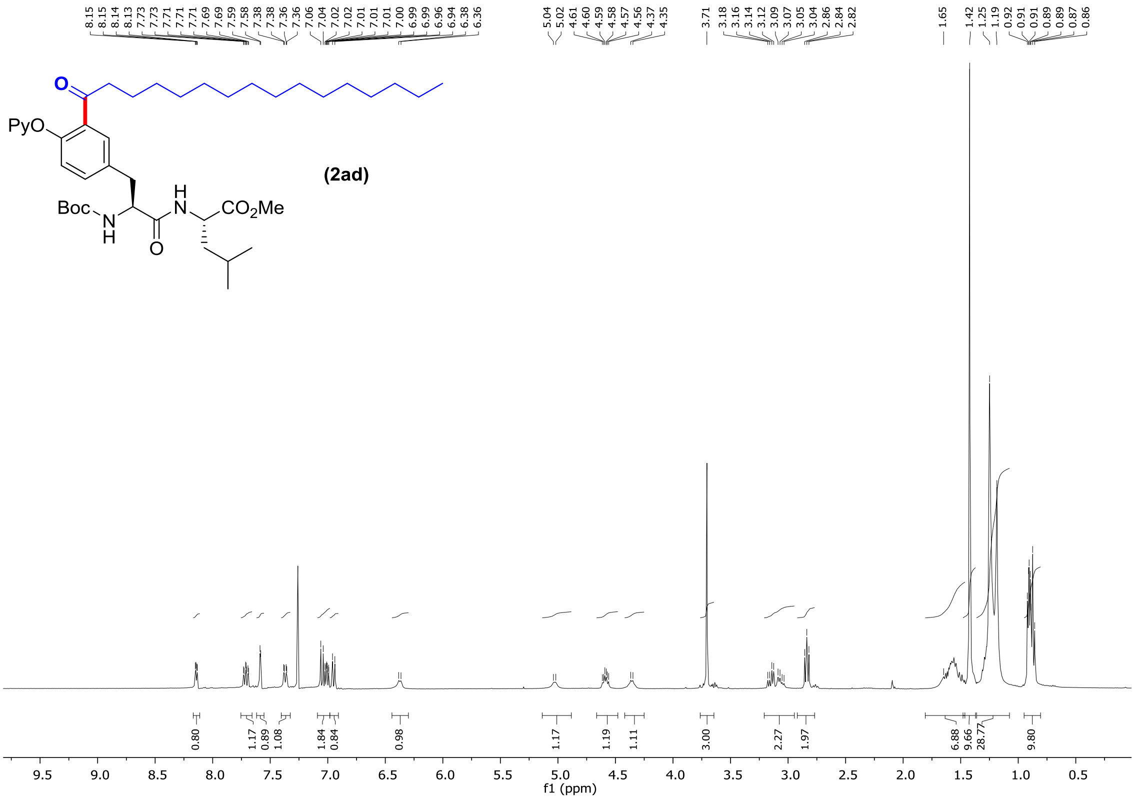

^{13}C NMR (101 MHz, $CDCl_3$)

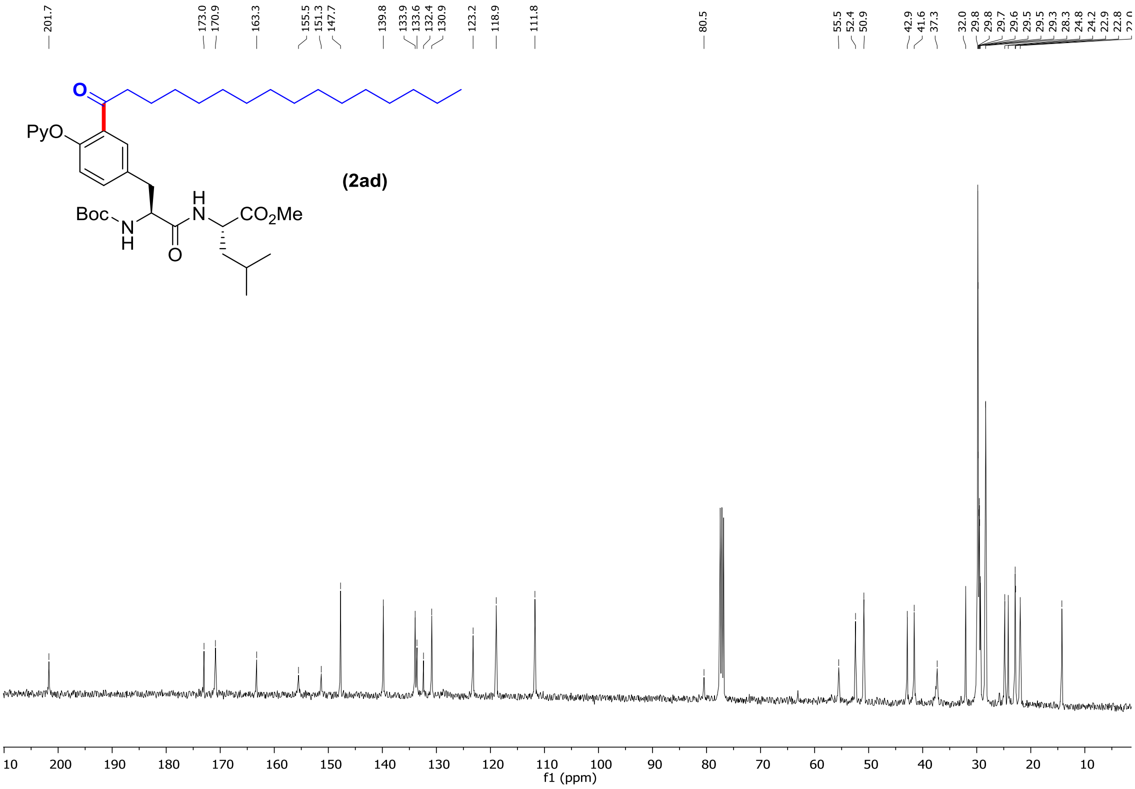

^{1}H NMR (400 MHz, $CDCl_3$)

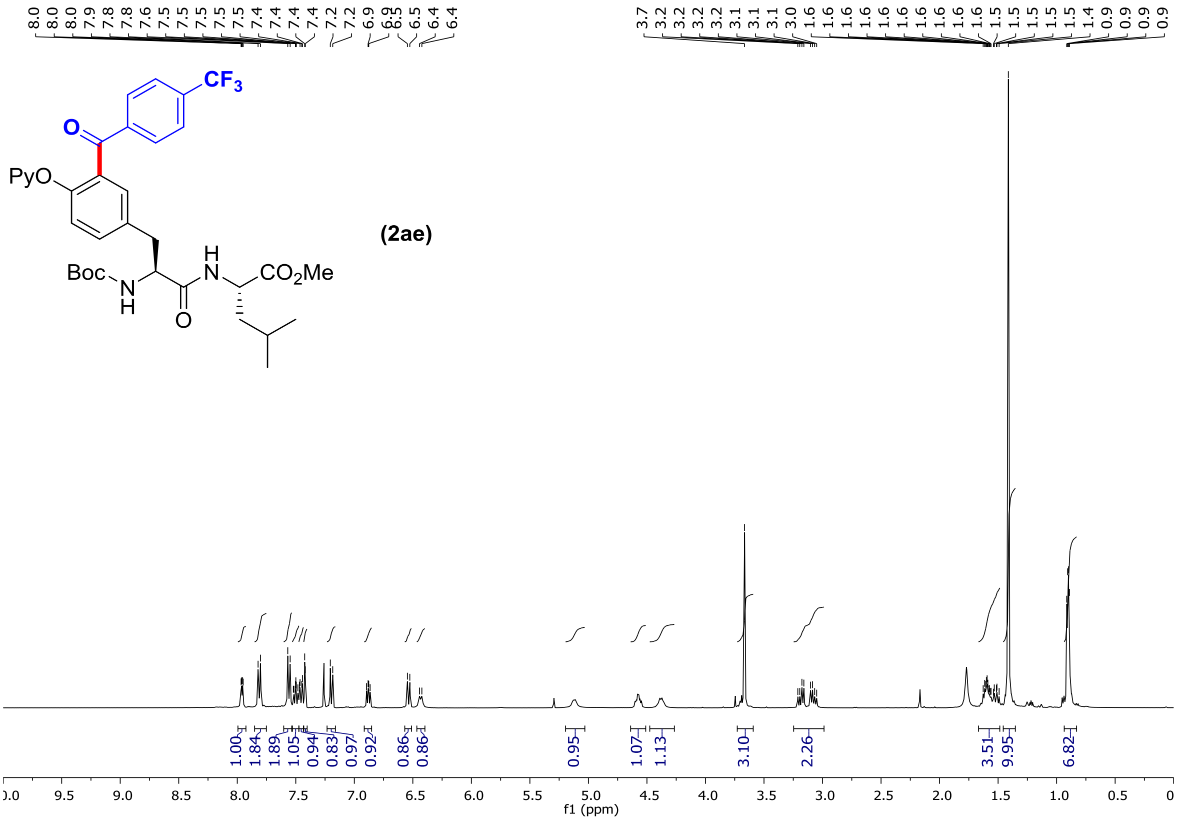

^{13}C NMR (101 MHz, $CDCl_3$)

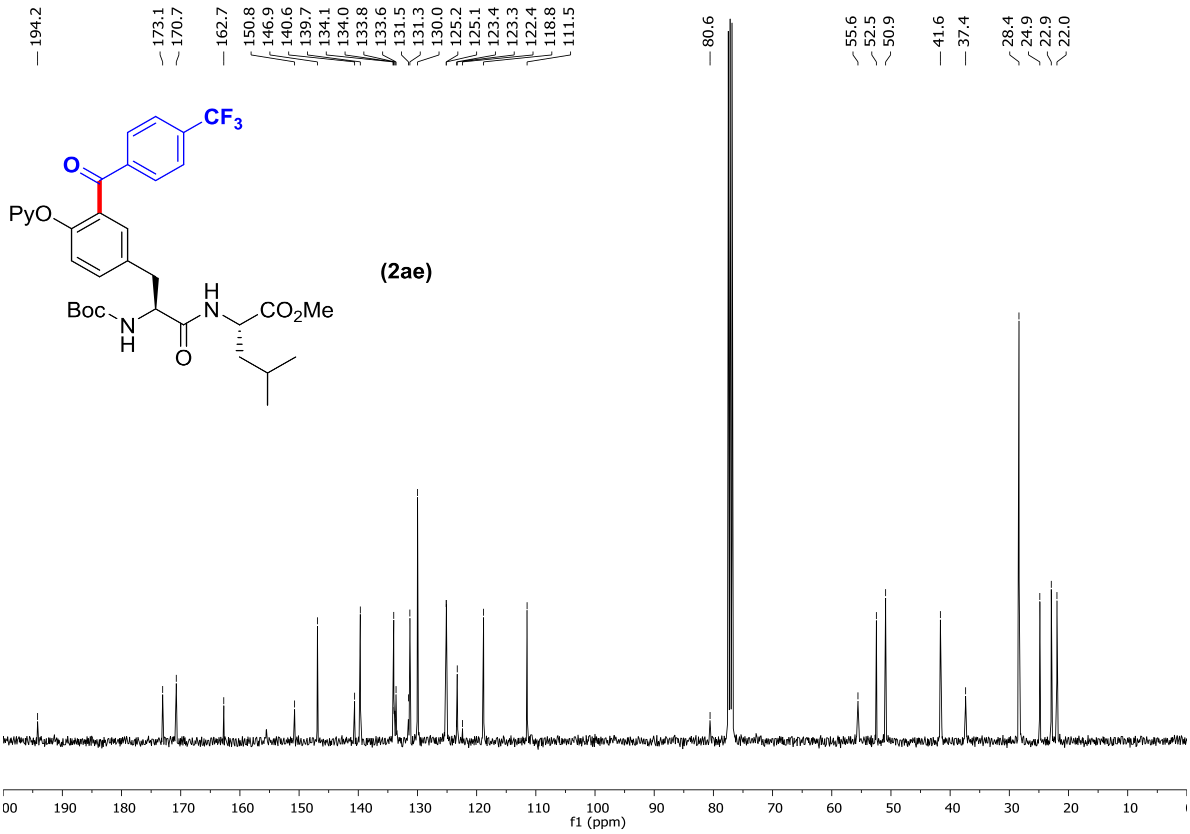

^{1}H NMR (400 MHz, $CDCl_3$)

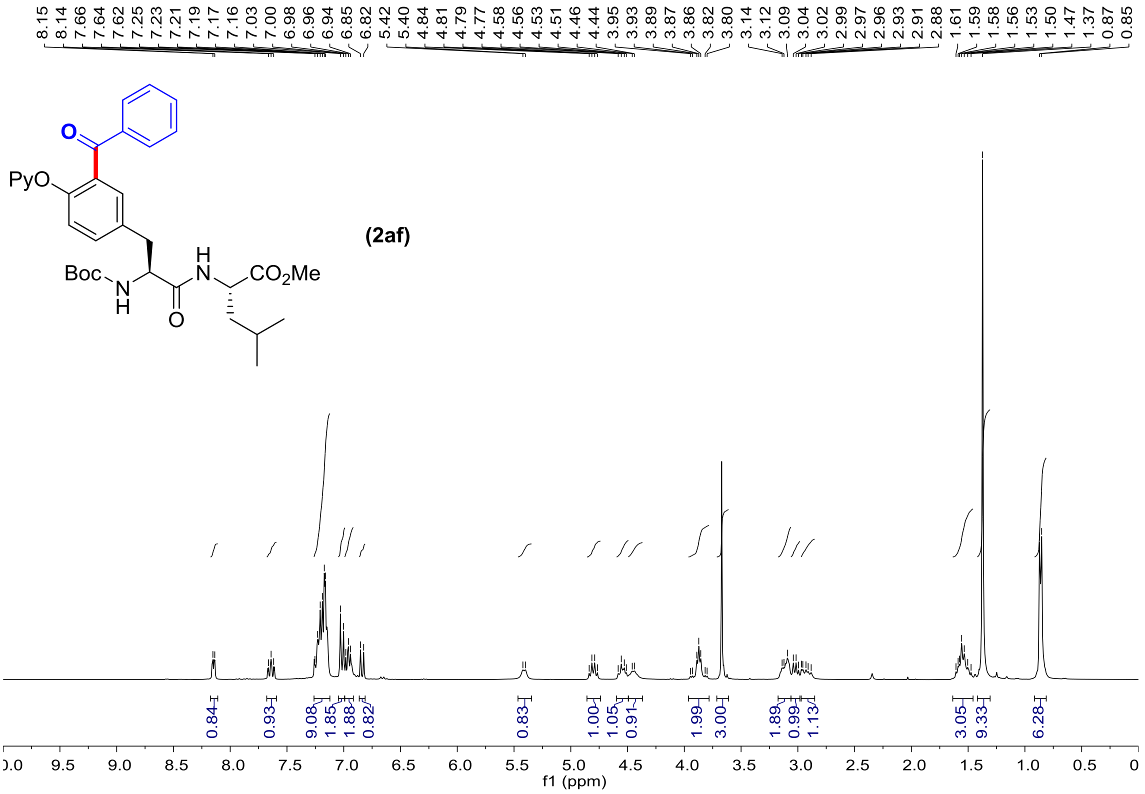

^{13}C NMR (101 MHz, $CDCl_3$)

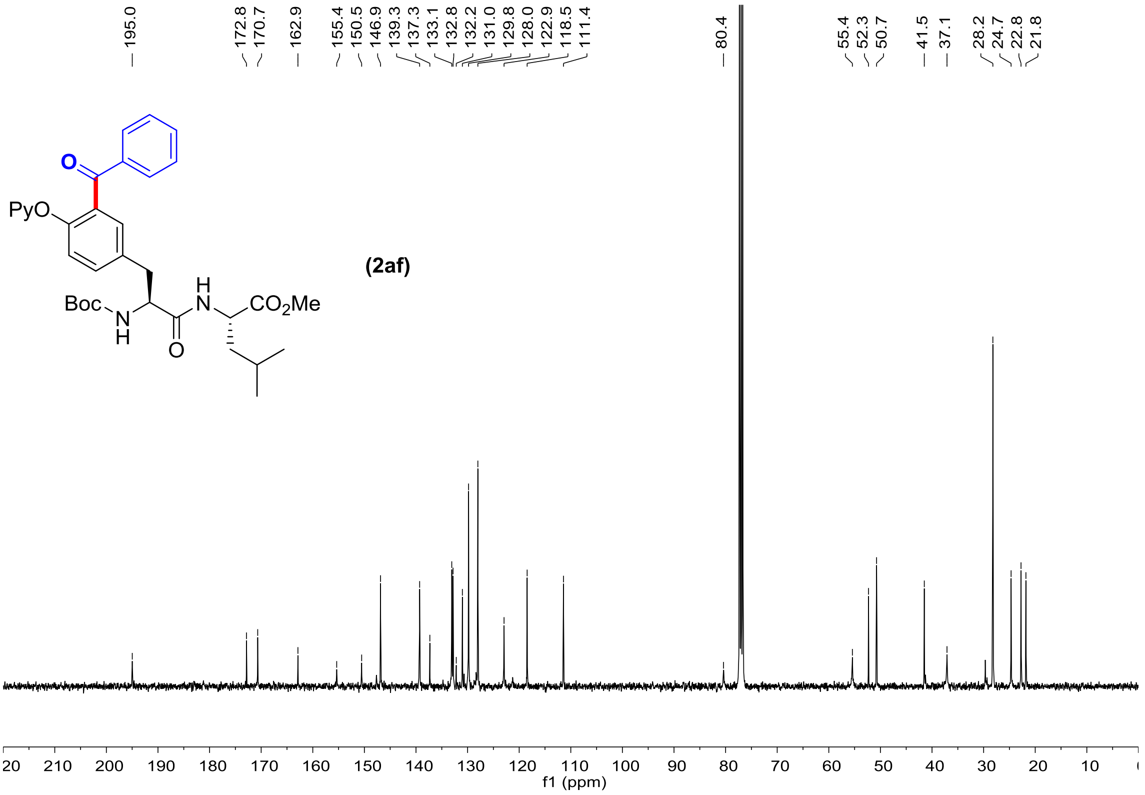

^{1}H NMR (400 MHz, CDCl$_3$)

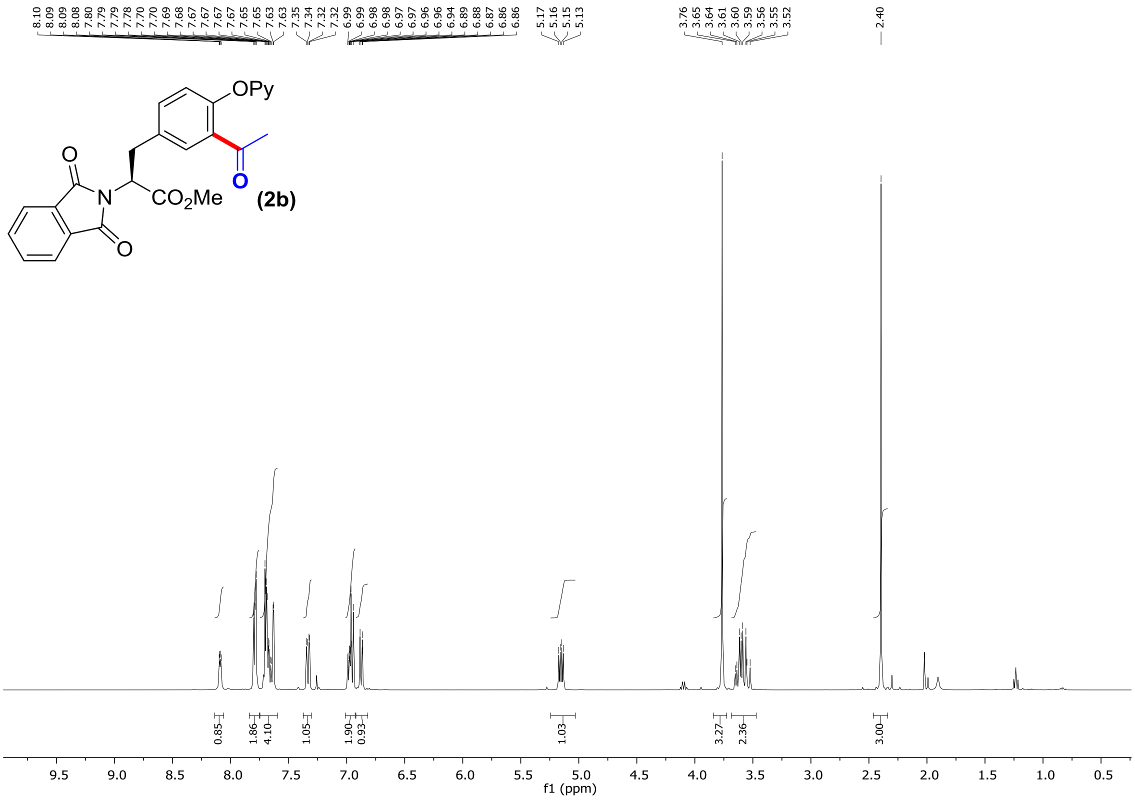

^{13}C NMR (101 MHz, CDCl$_3$)

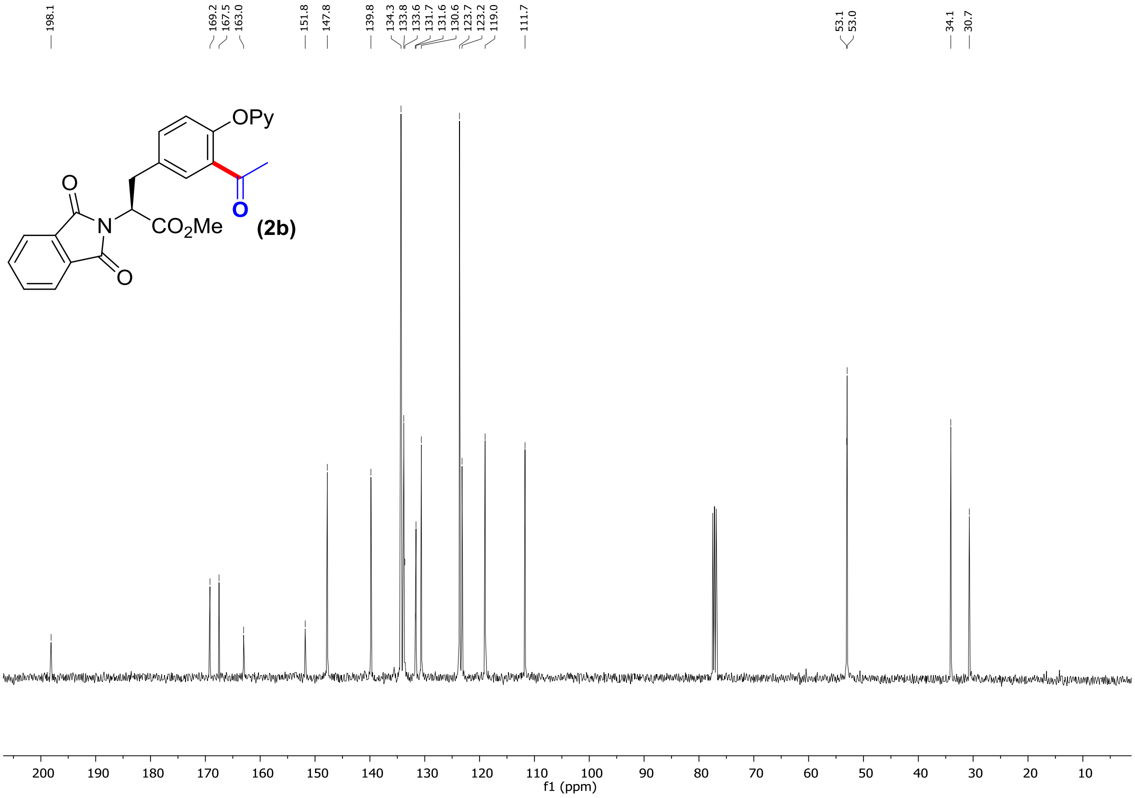

^{1}H NMR (400 MHz, $CDCl_3$)

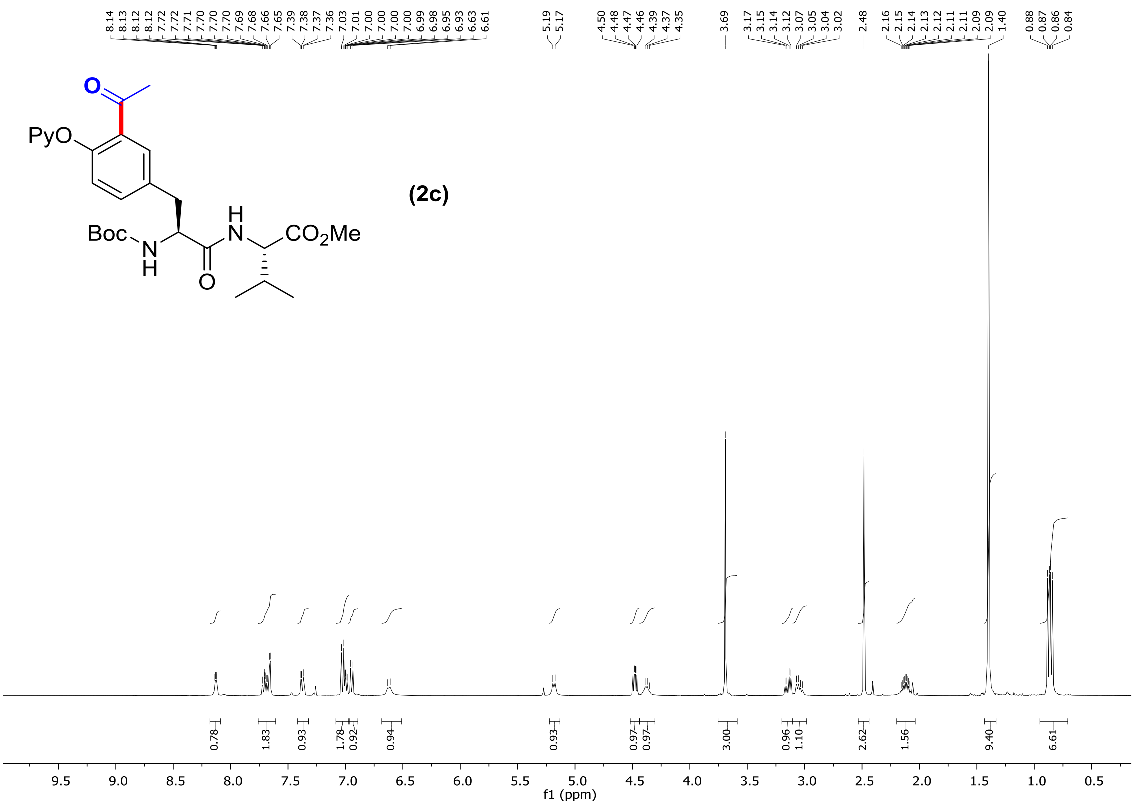

^{13}C NMR (101 MHz, $CDCl_3$)

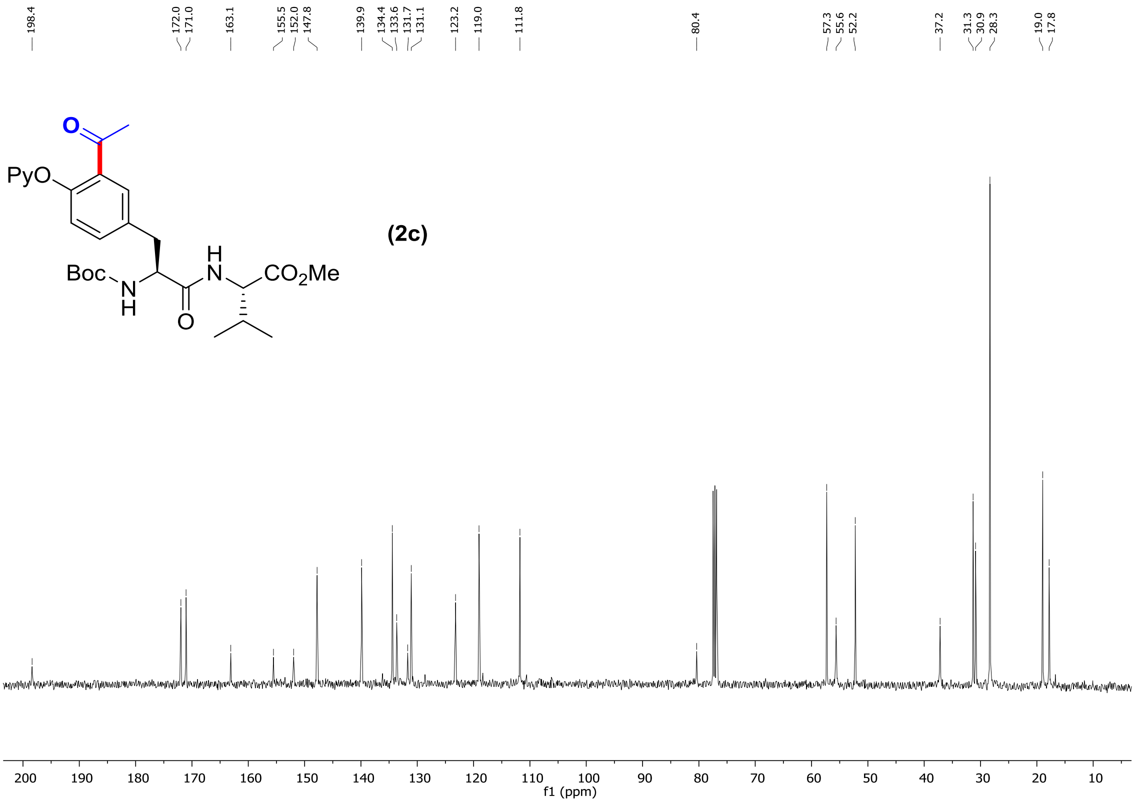

^{1}H NMR (400 MHz, $CDCl_3$)

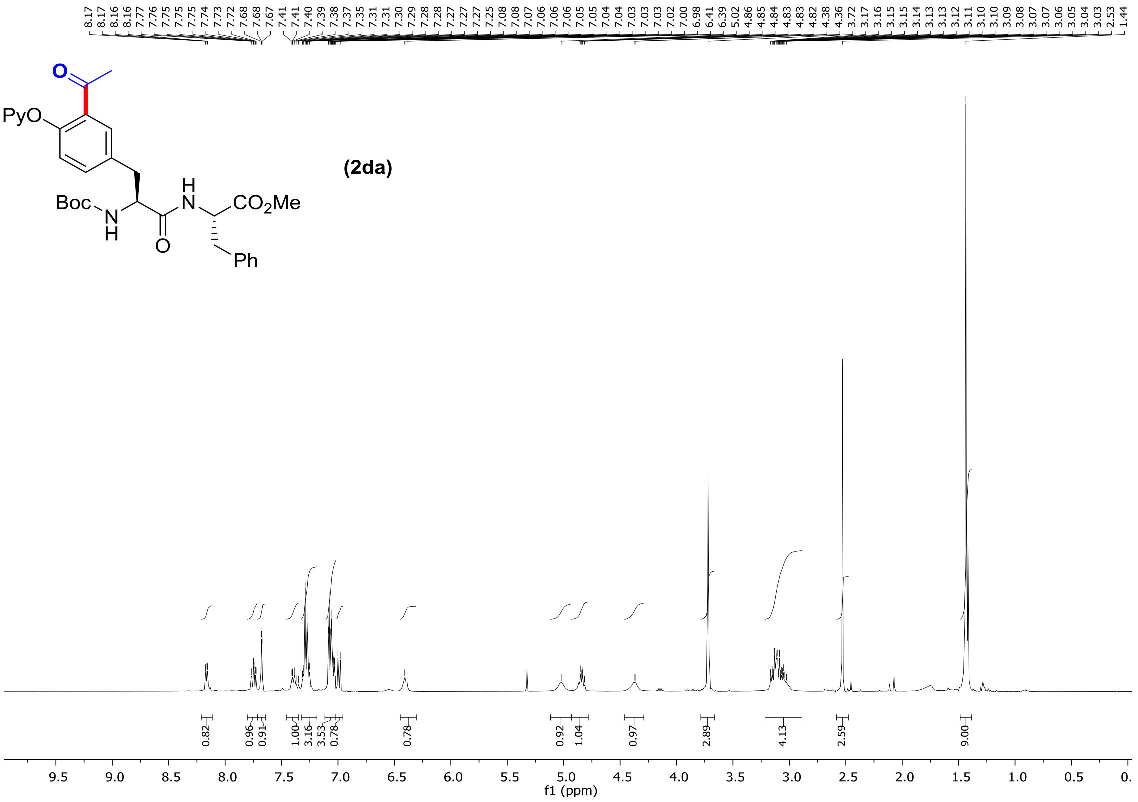

^{13}C NMR (101 MHz, $CDCl_3$)

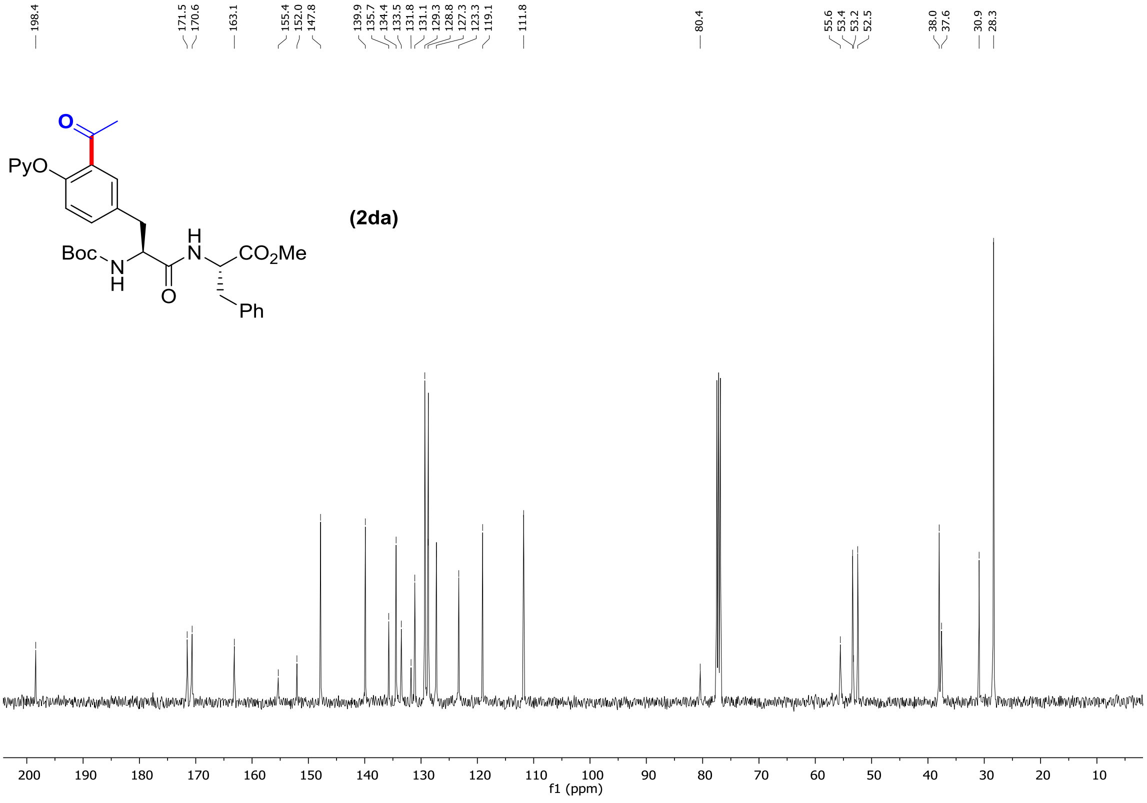

^{1}H NMR (300 MHz, $CDCl_3$)

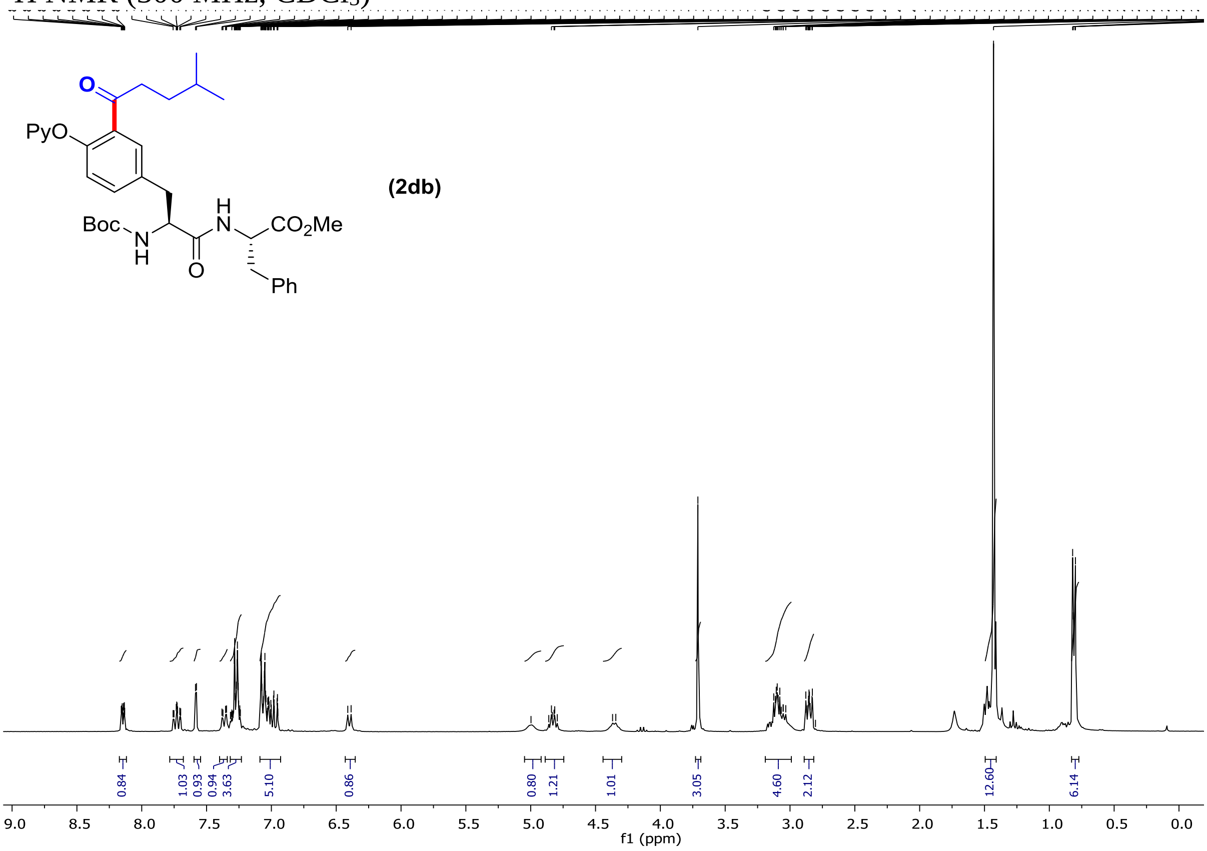

^{13}C NMR (75 MHz, $CDCl_3$)

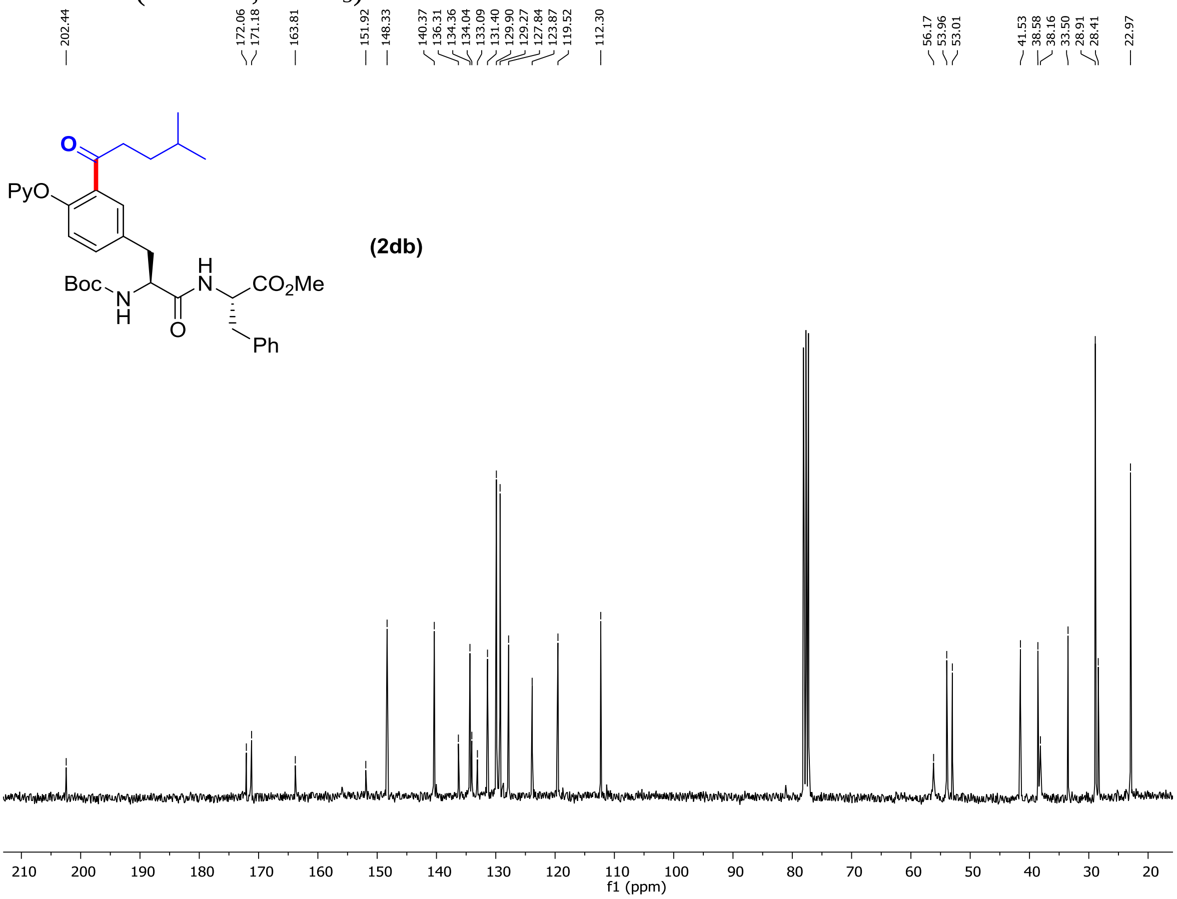

^{1}H NMR (400 MHz, $CDCl_3$)

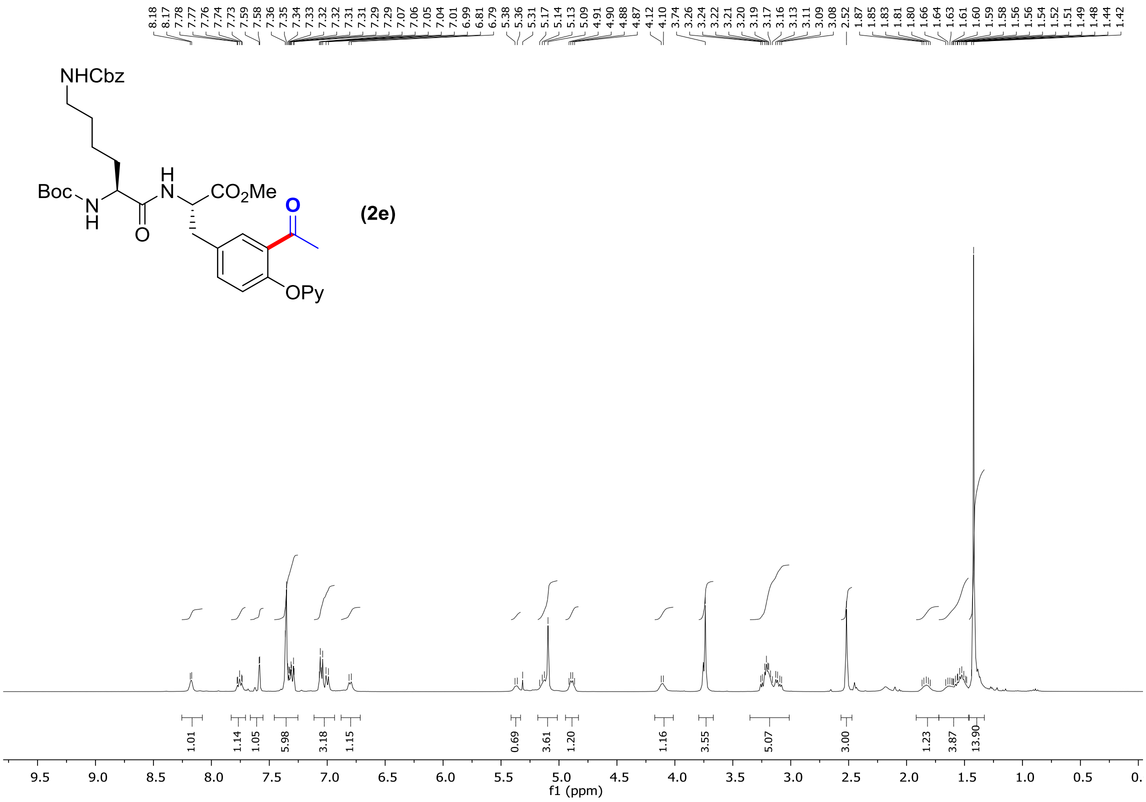

^{13}C NMR (101 MHz, $CDCl_3$)

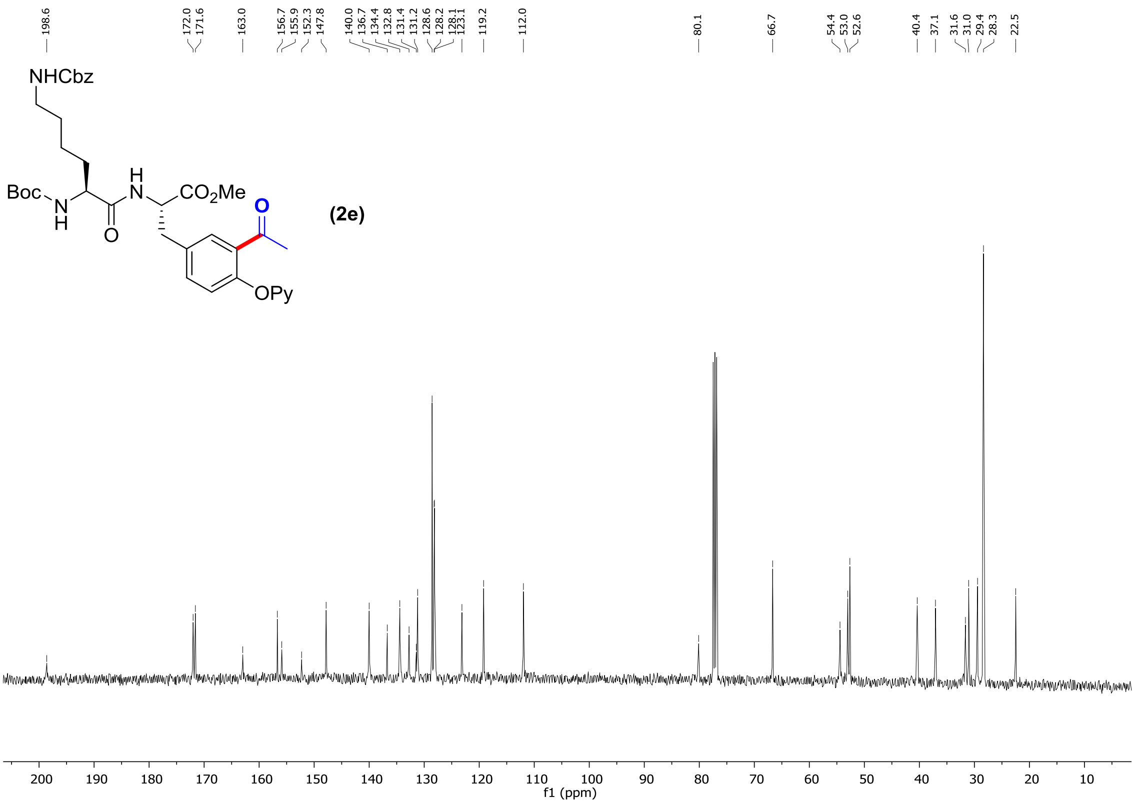

^{1}H NMR (400 MHz, $CDCl_3$)

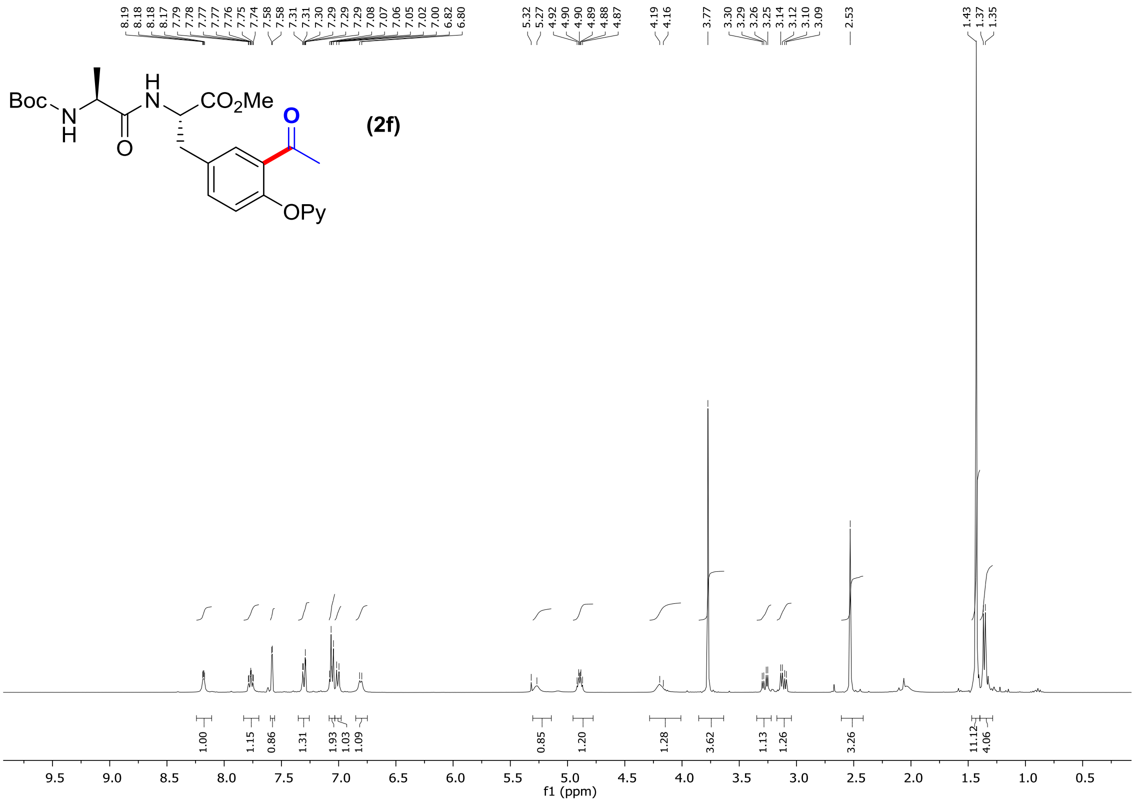

^{13}C NMR (101 MHz, $CDCl_3$)

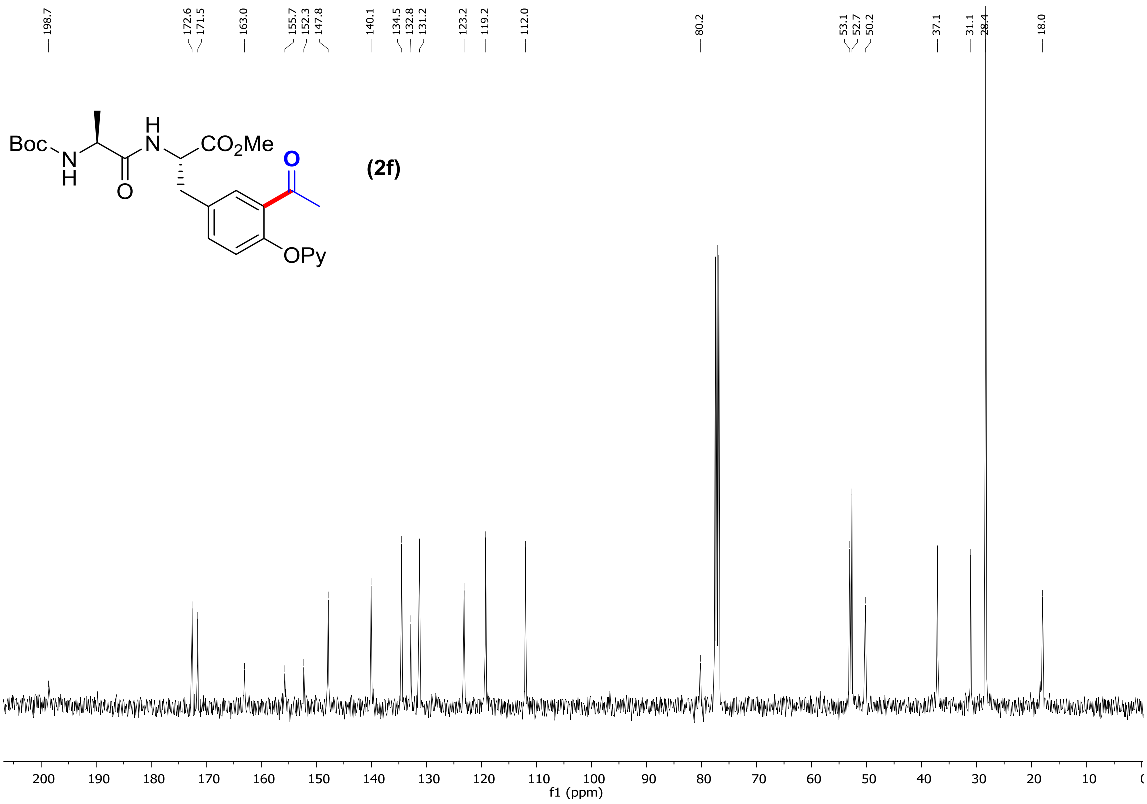

^{1}H NMR (300 MHz, $CDCl_3$)

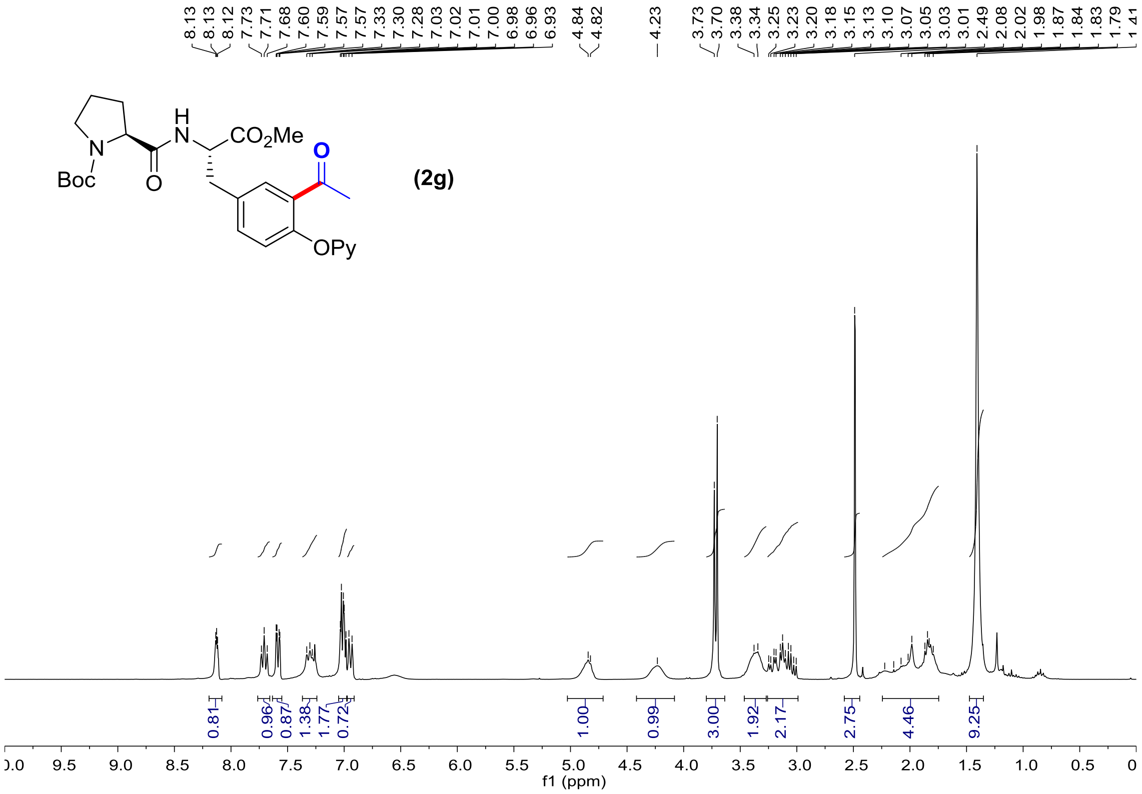

^{13}C NMR (75 MHz, $CDCl_3$)

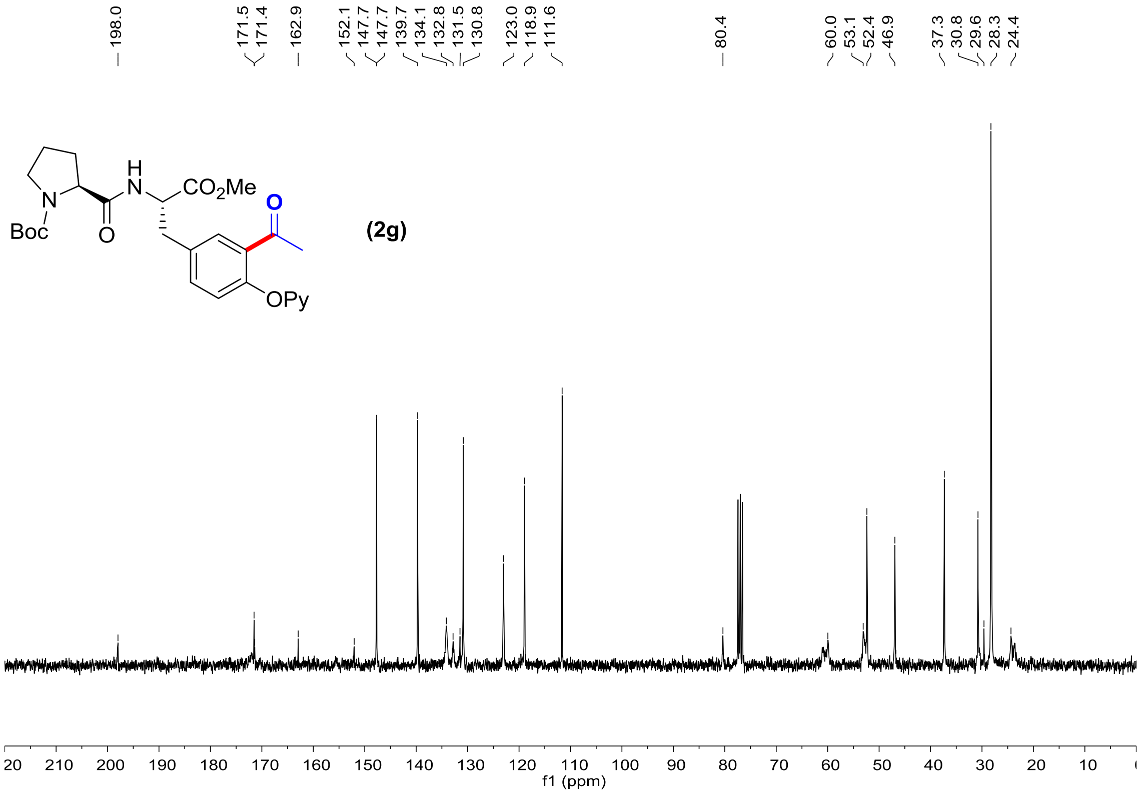

^{1}H NMR (400 MHz, $CDCl_3$)

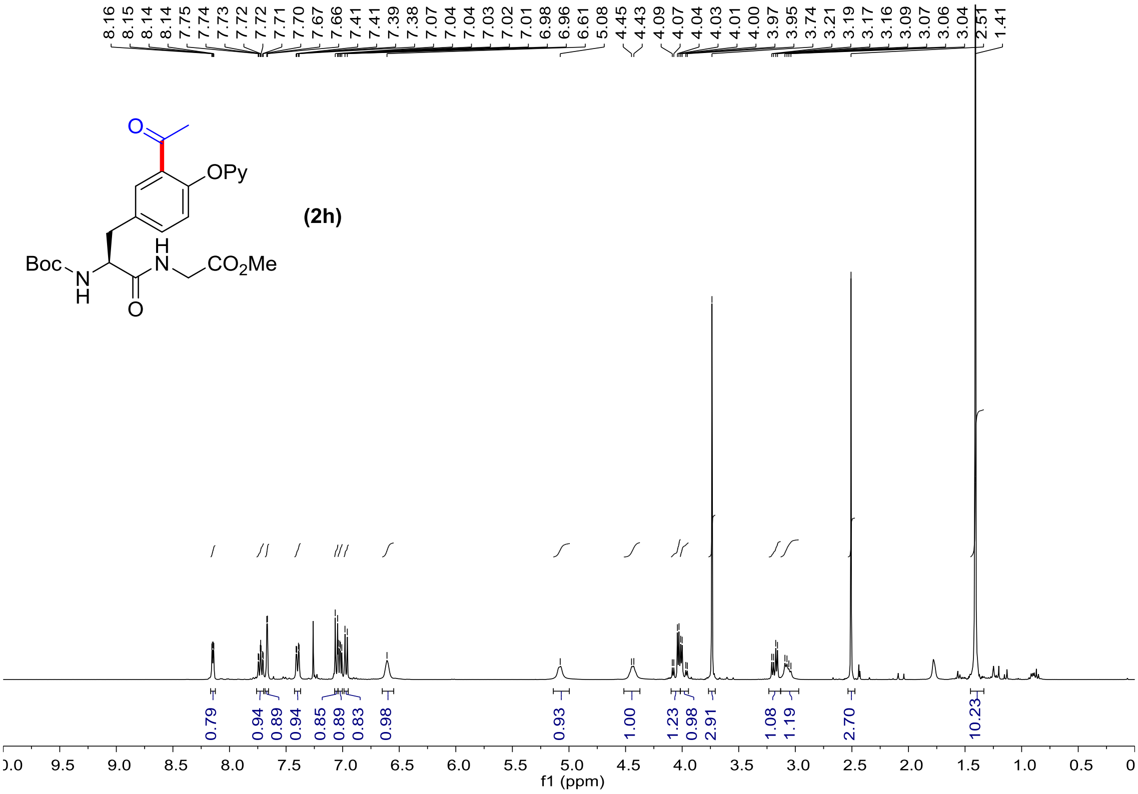

^{13}C NMR (101 MHz, $CDCl_3$)

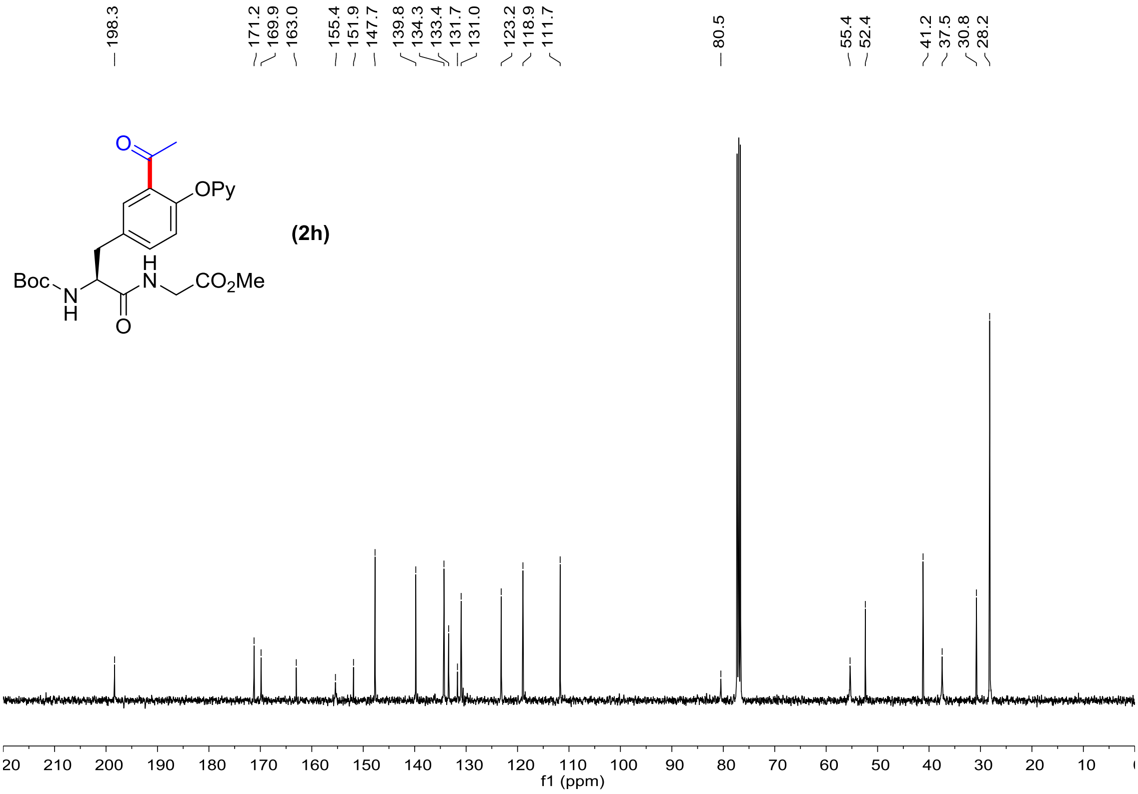

^{1}H NMR (300 MHz, $CDCl_3$)

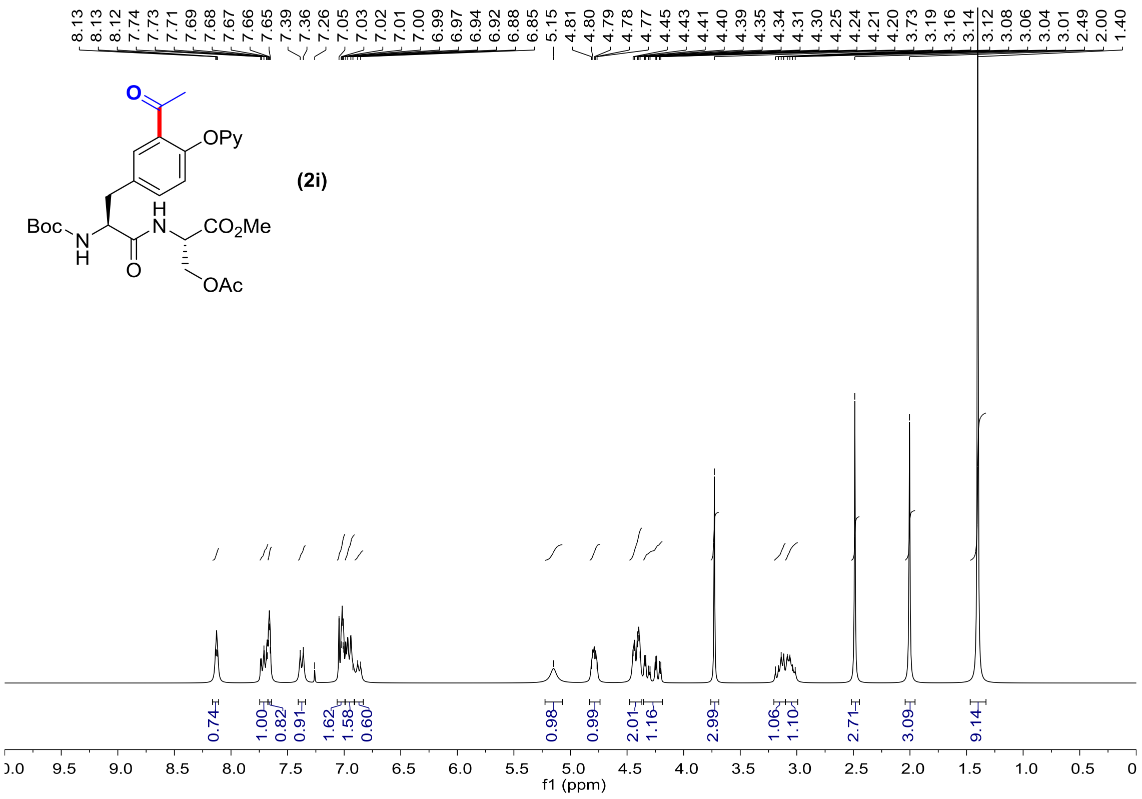

^{13}C NMR (75 MHz, $CDCl_3$)

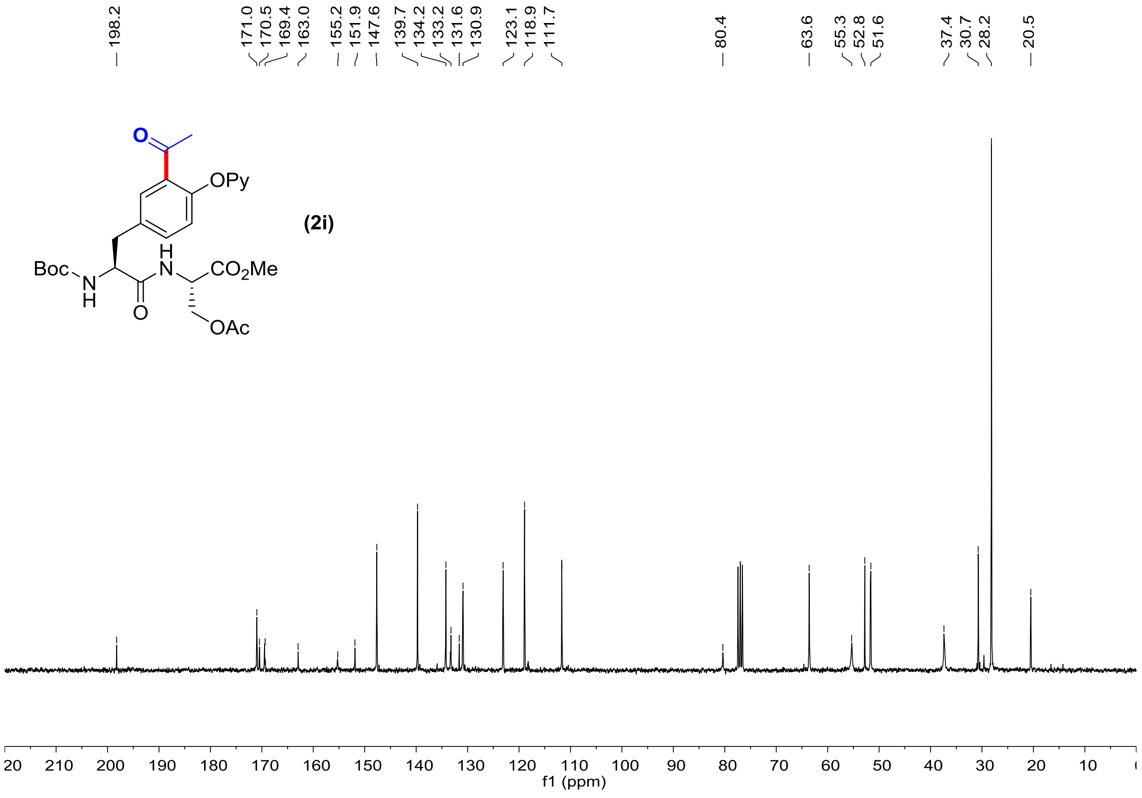

^{1}H NMR (300 MHz, $CDCl_3$)

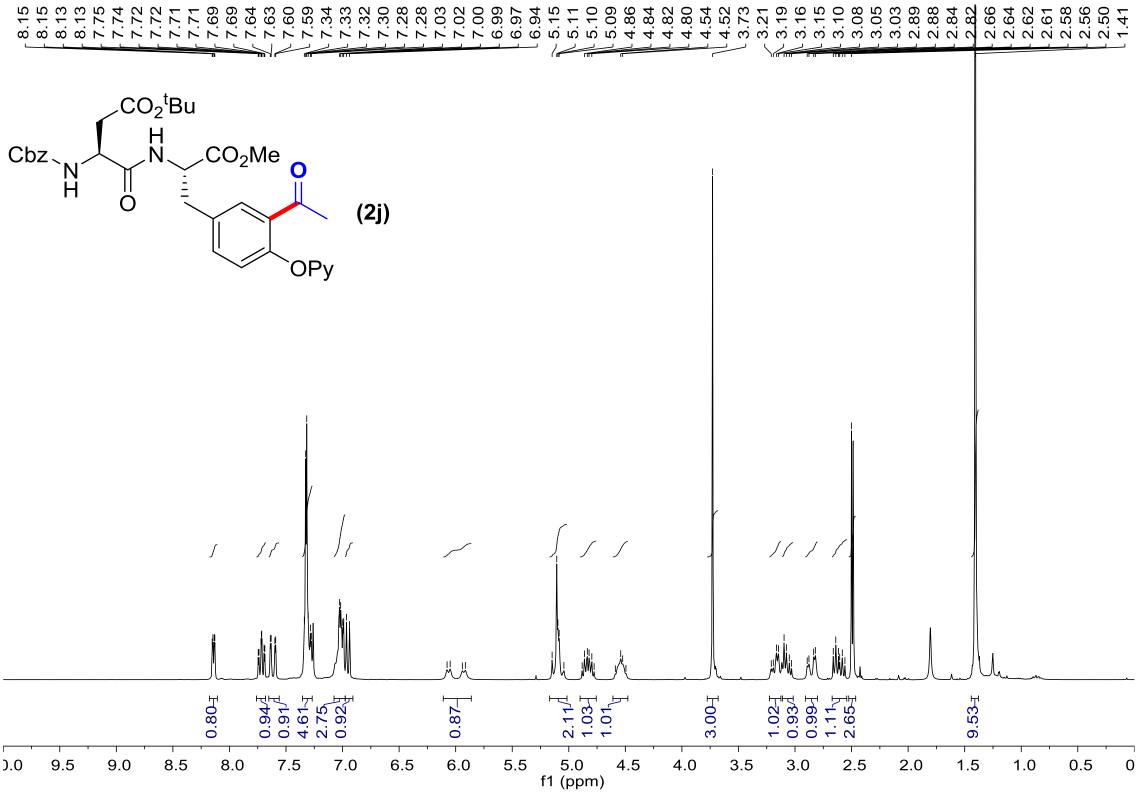

^{13}C NMR (75 MHz, $CDCl_3$)

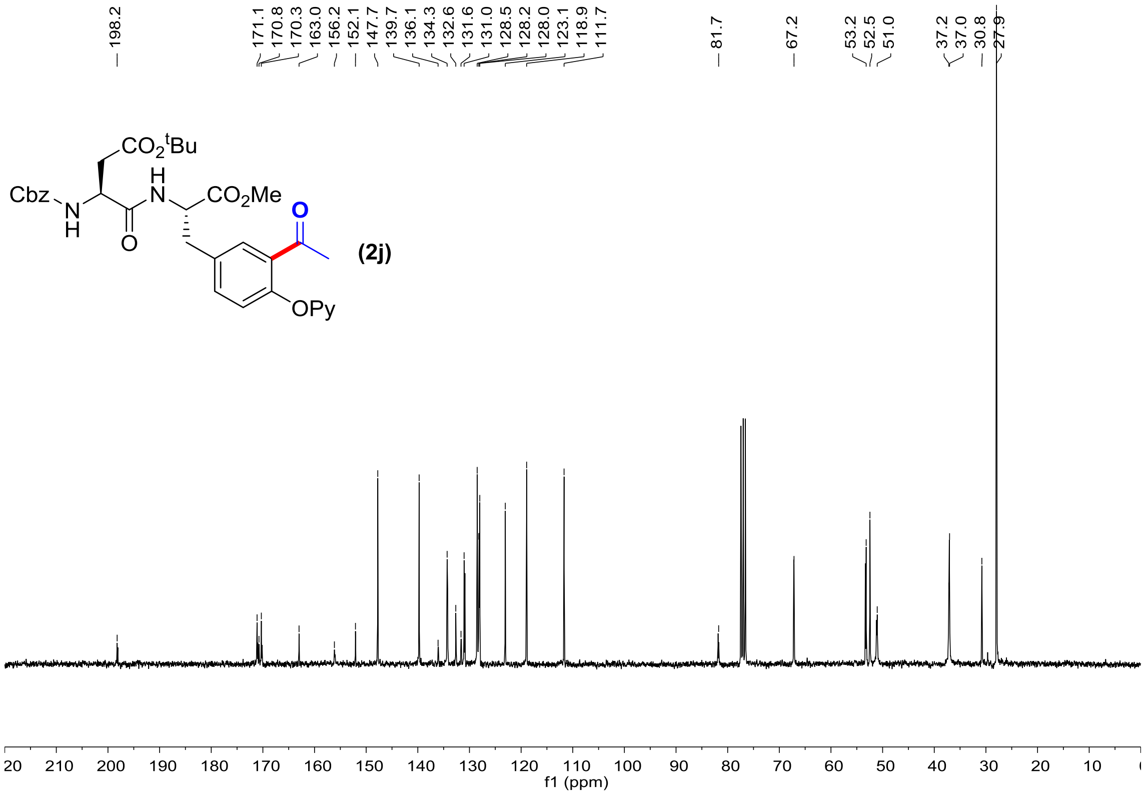

^{1}H NMR (400 MHz, $CDCl_3$)

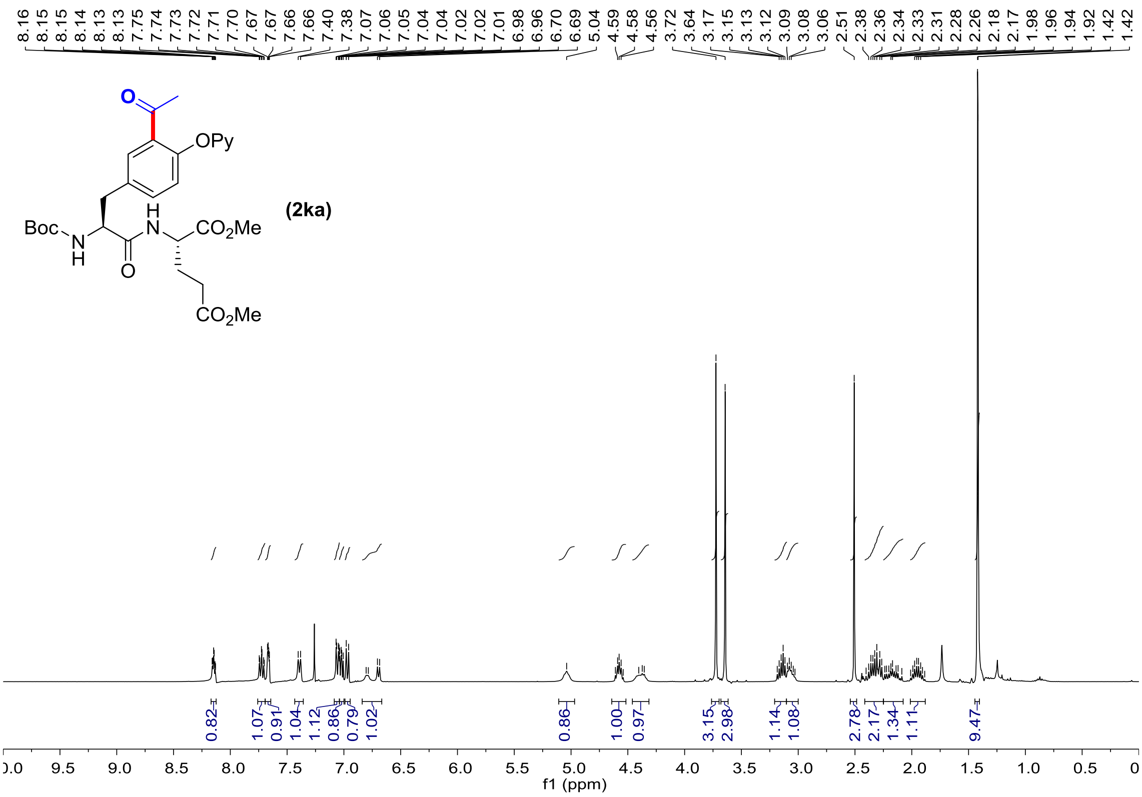

^{13}C NMR (101 MHz, $CDCl_3$)

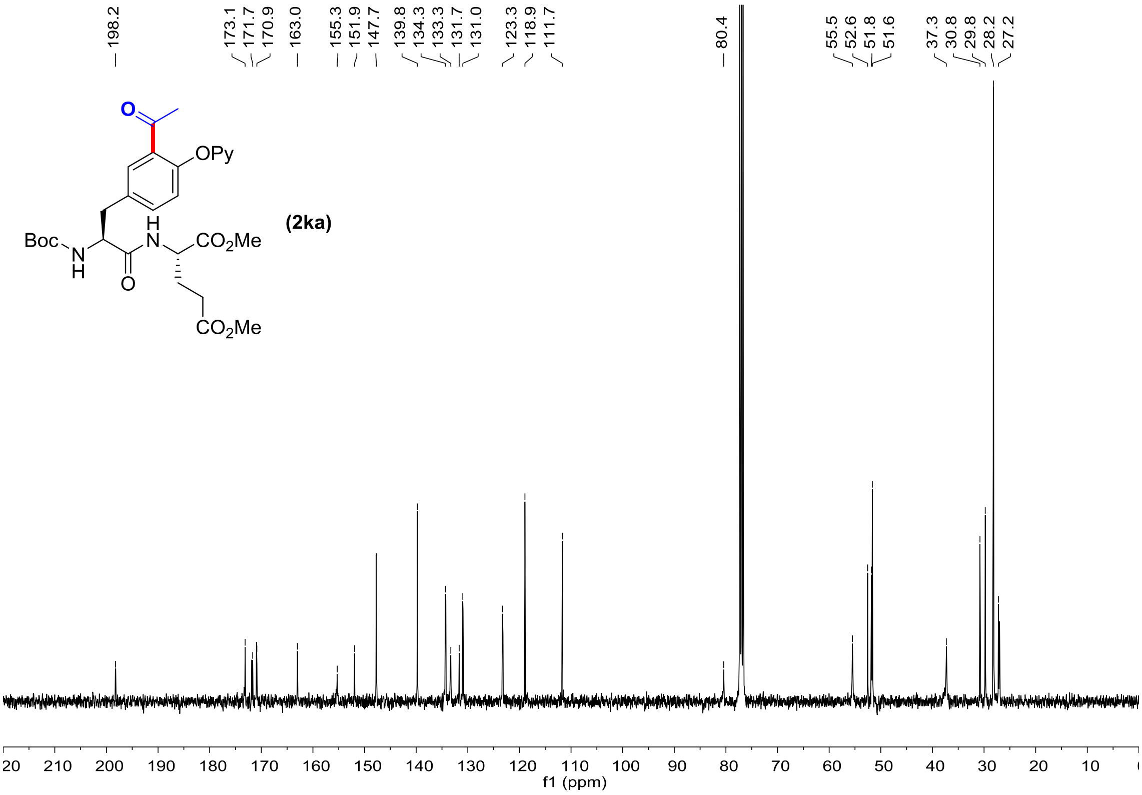

^{1}H NMR (400 MHz, $CDCl_3$)

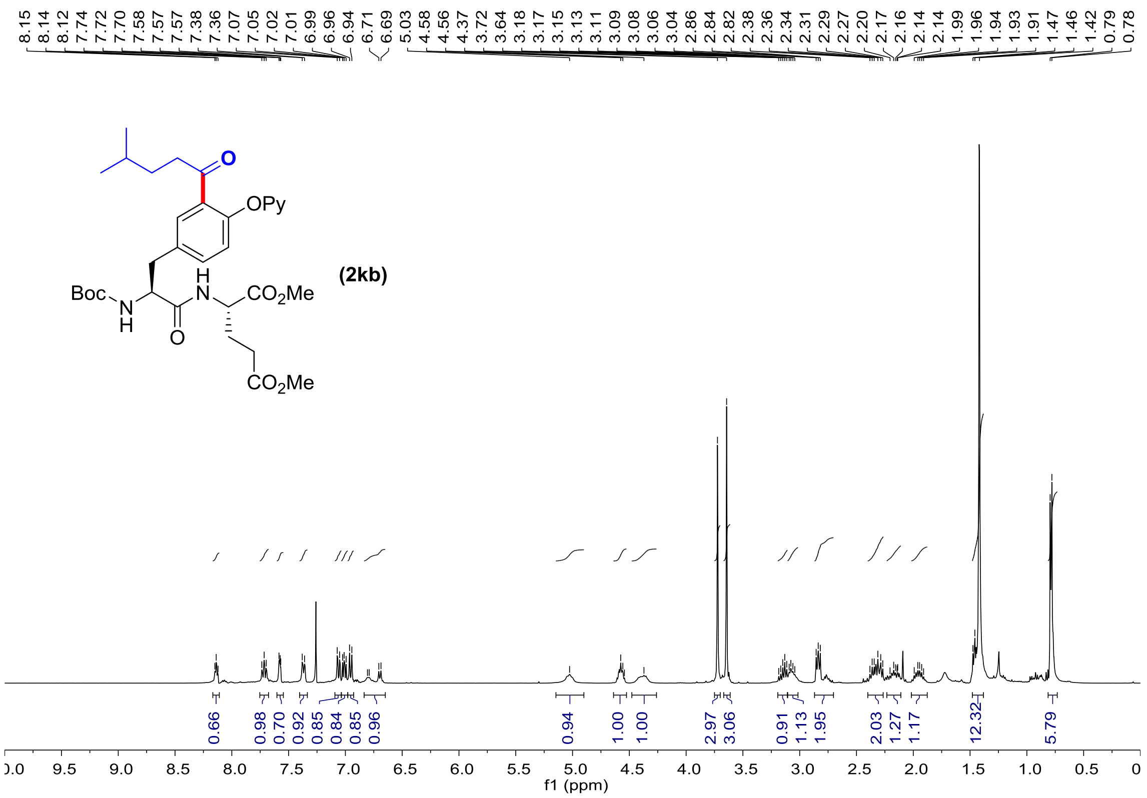

^{13}C NMR (101 MHz, $CDCl_3$)

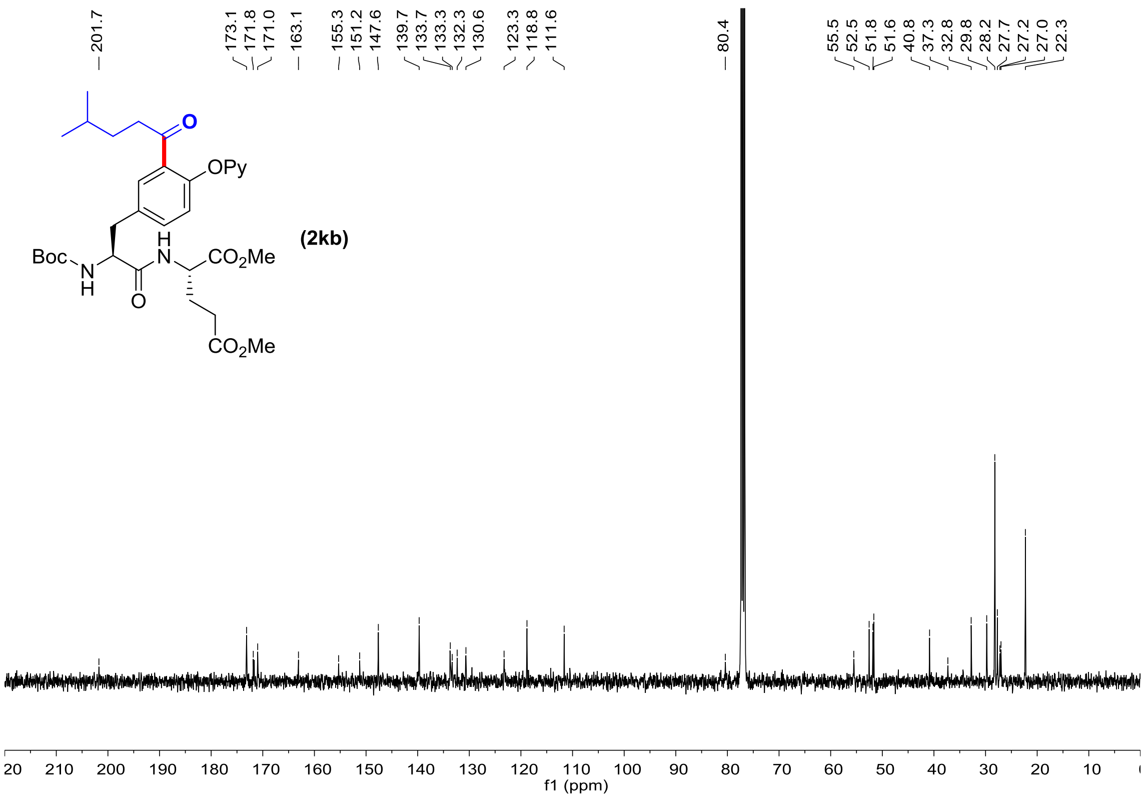

^{1}H NMR (400 MHz, $CDCl_3$)

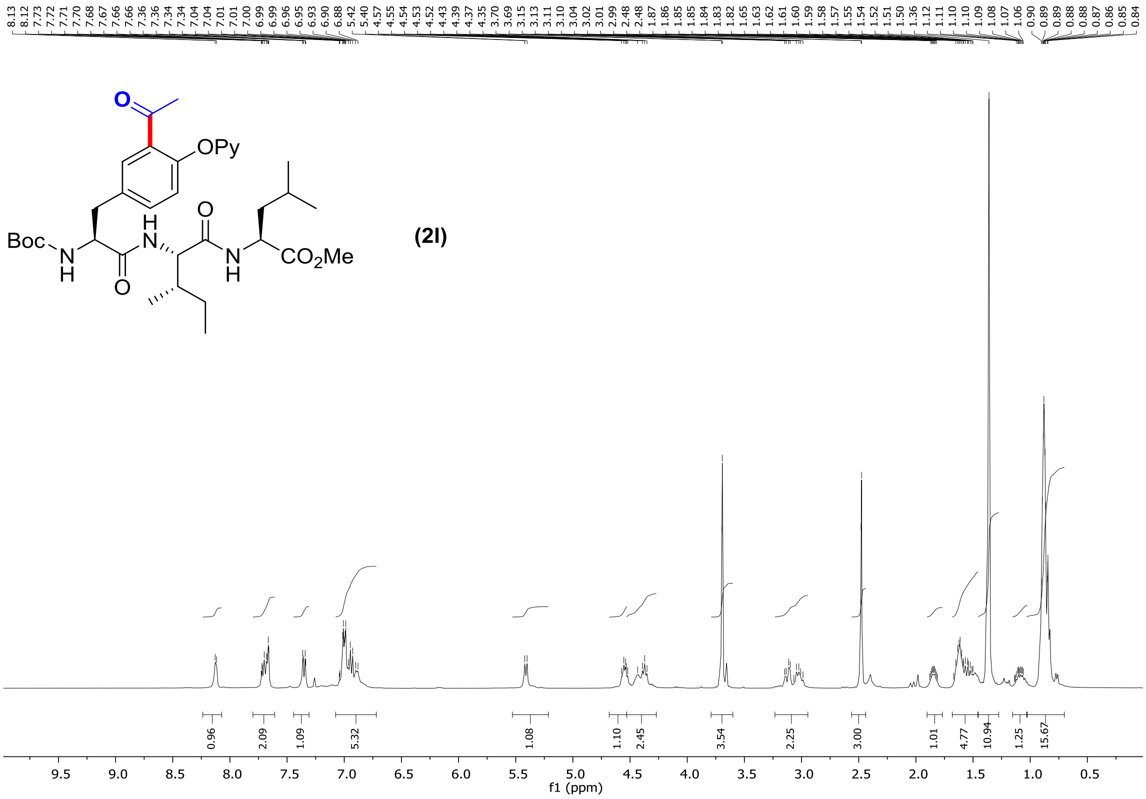

^{13}C NMR (101 MHz, $CDCl_3$)

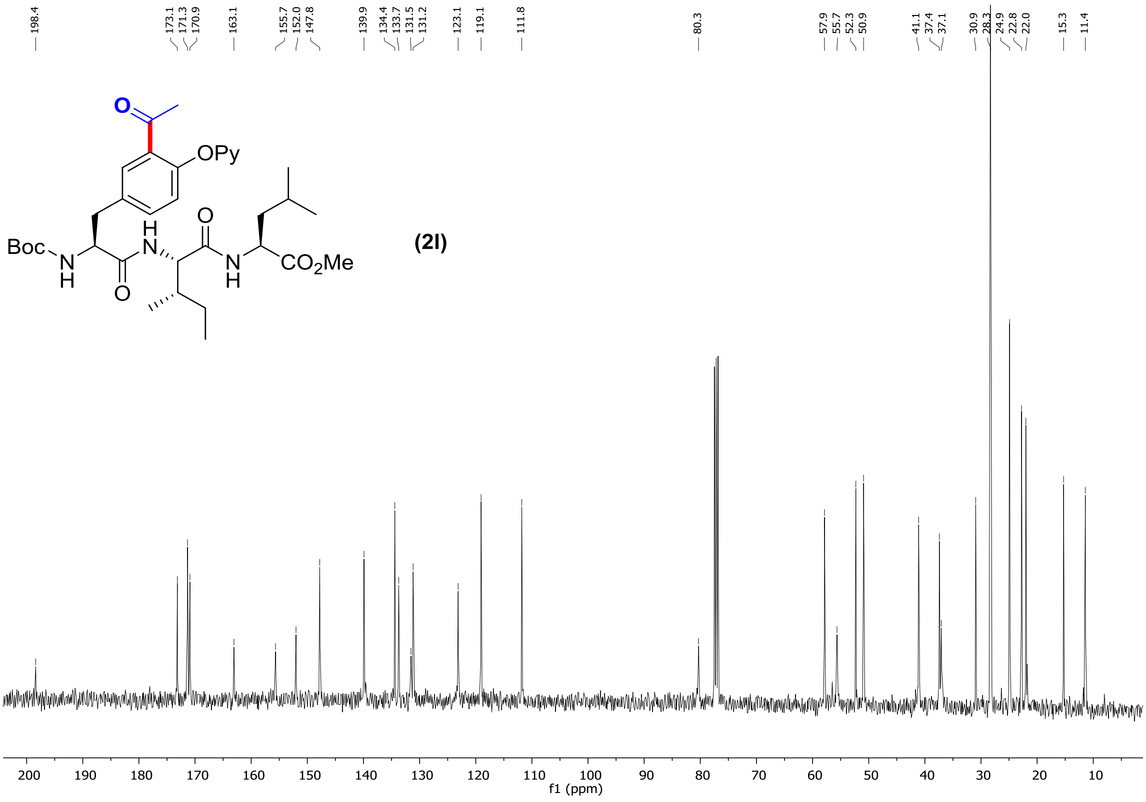

^{1}H NMR (300 MHz, $CDCl_3$)

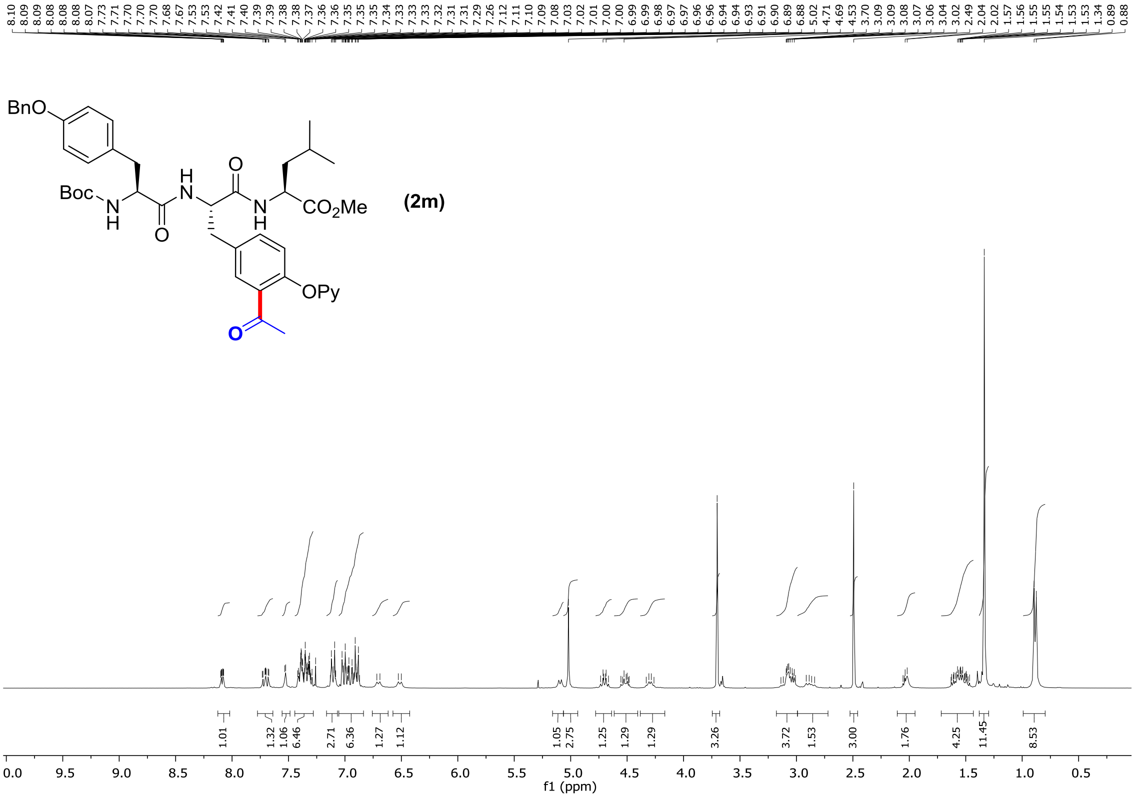

^{13}C NMR (75 MHz, $CDCl_3$)

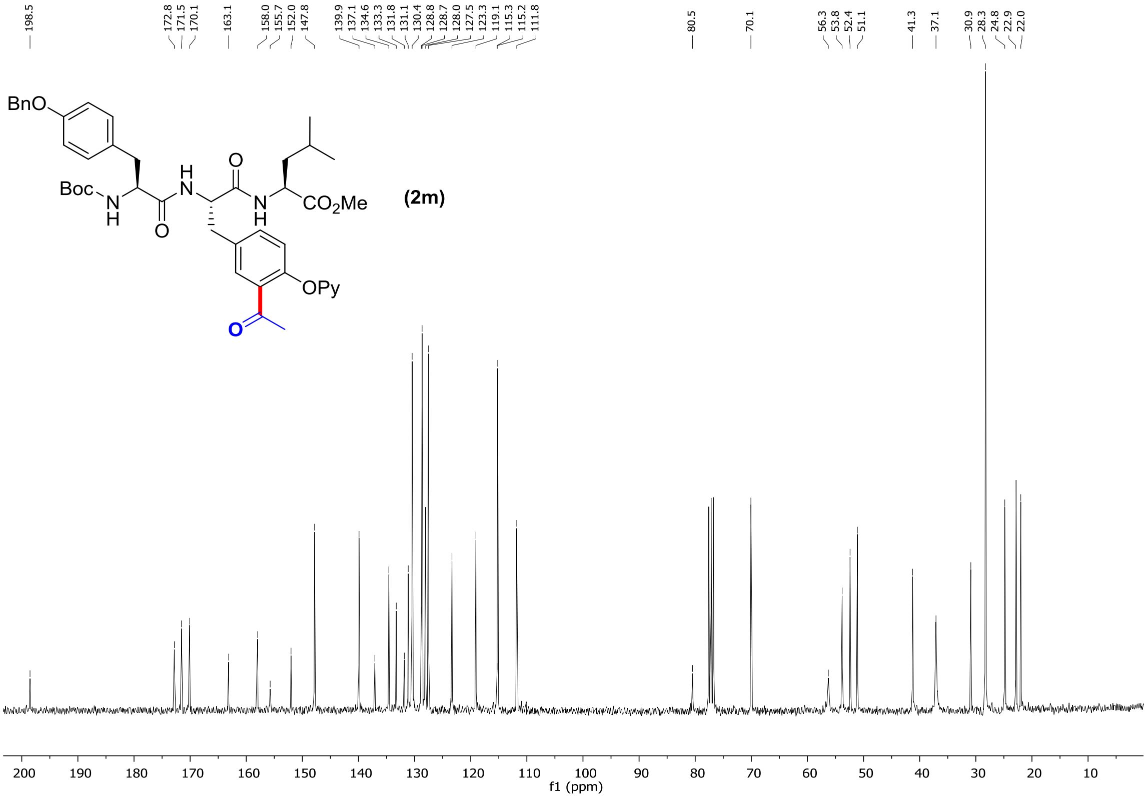

^{1}H NMR (500 MHz, DMSO-d_6 at 80 ºC)

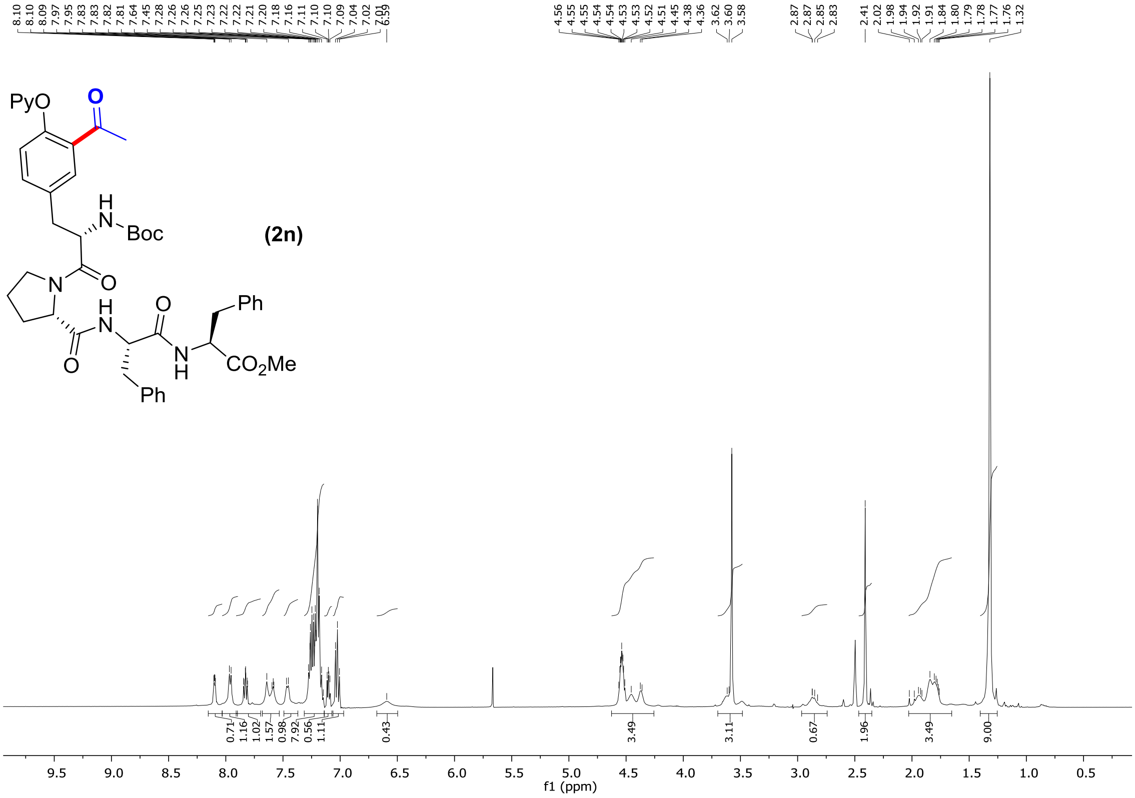

^{13}C NMR (126 MHz, DMSO-d_6 at 80 ºC)

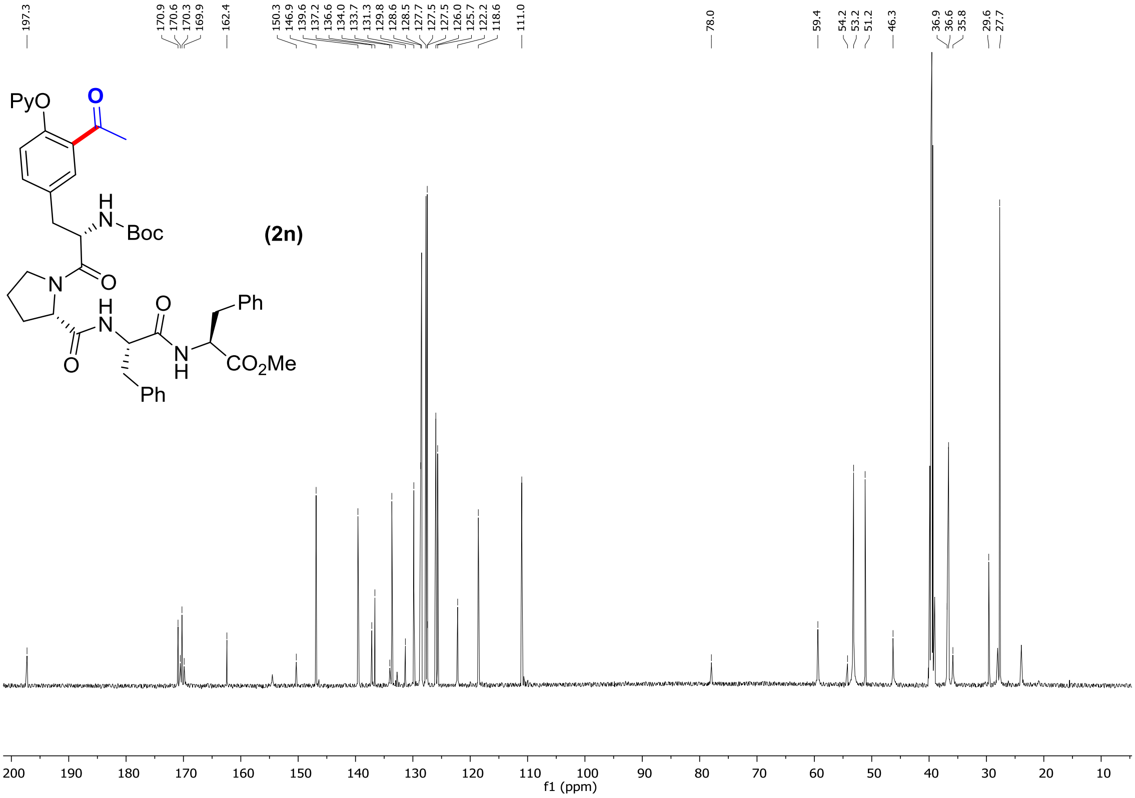

^{1}H NMR (500 MHz, DMSO-d_6 at 80 ºC)

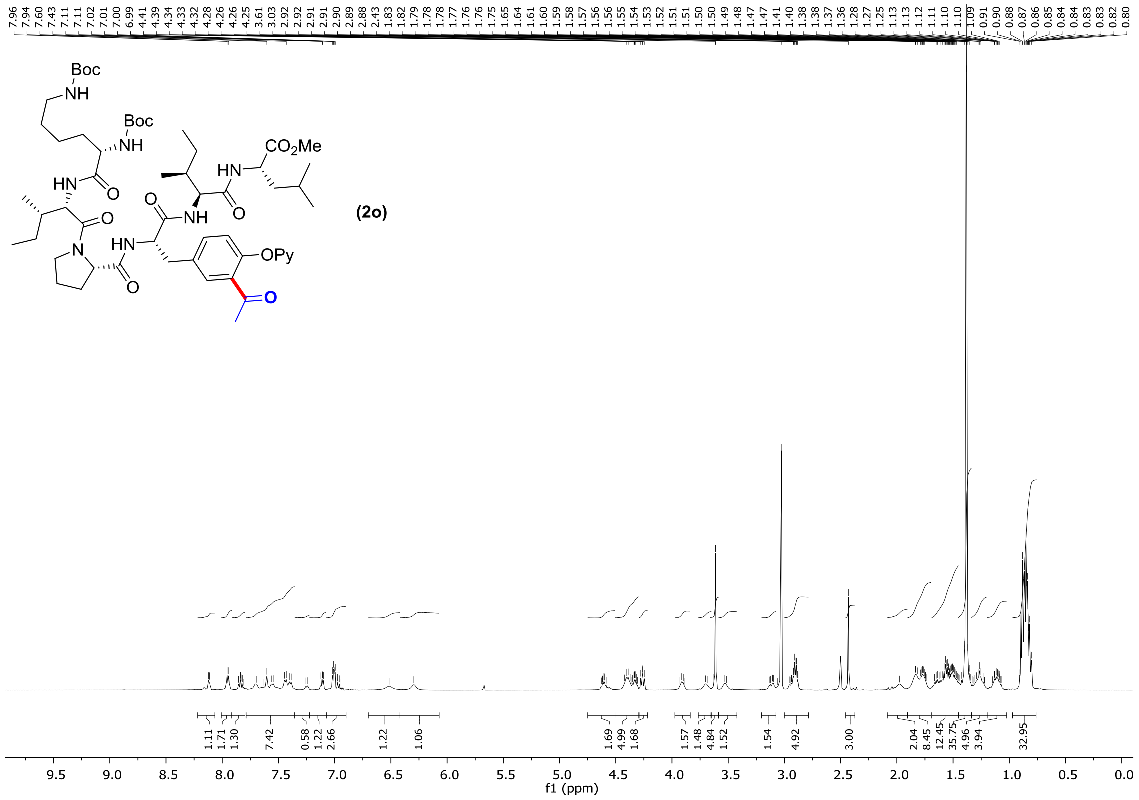

^{13}C NMR (126 MHz, DMSO-d_6 at 80 ºC)

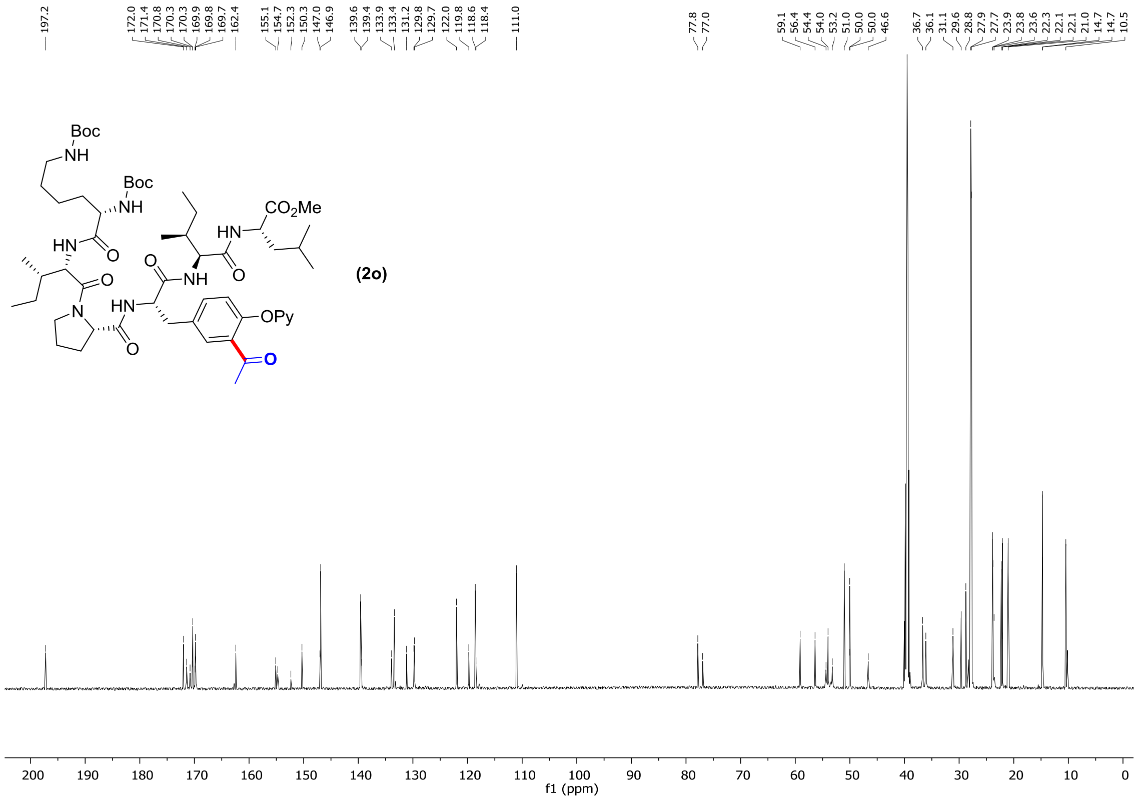

^{1}H NMR (300 MHz, $CDCl_3$)

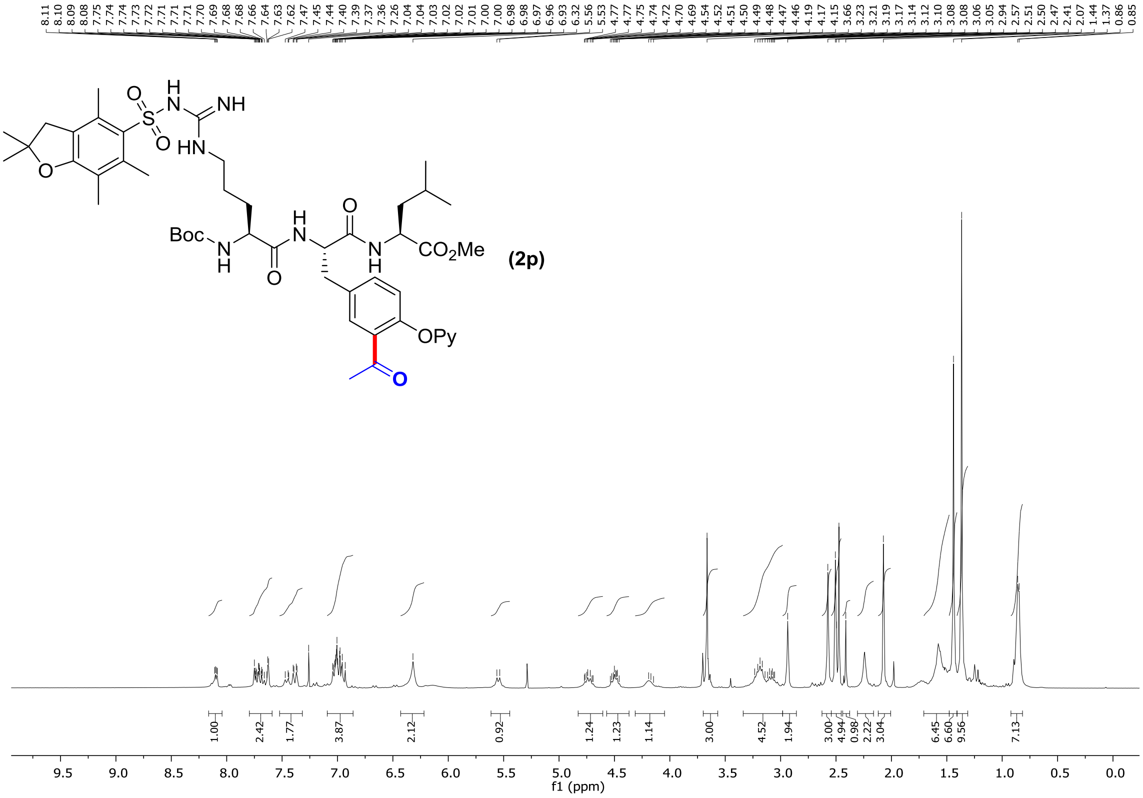

^{13}C NMR (75 MHz, $CDCl_3$)

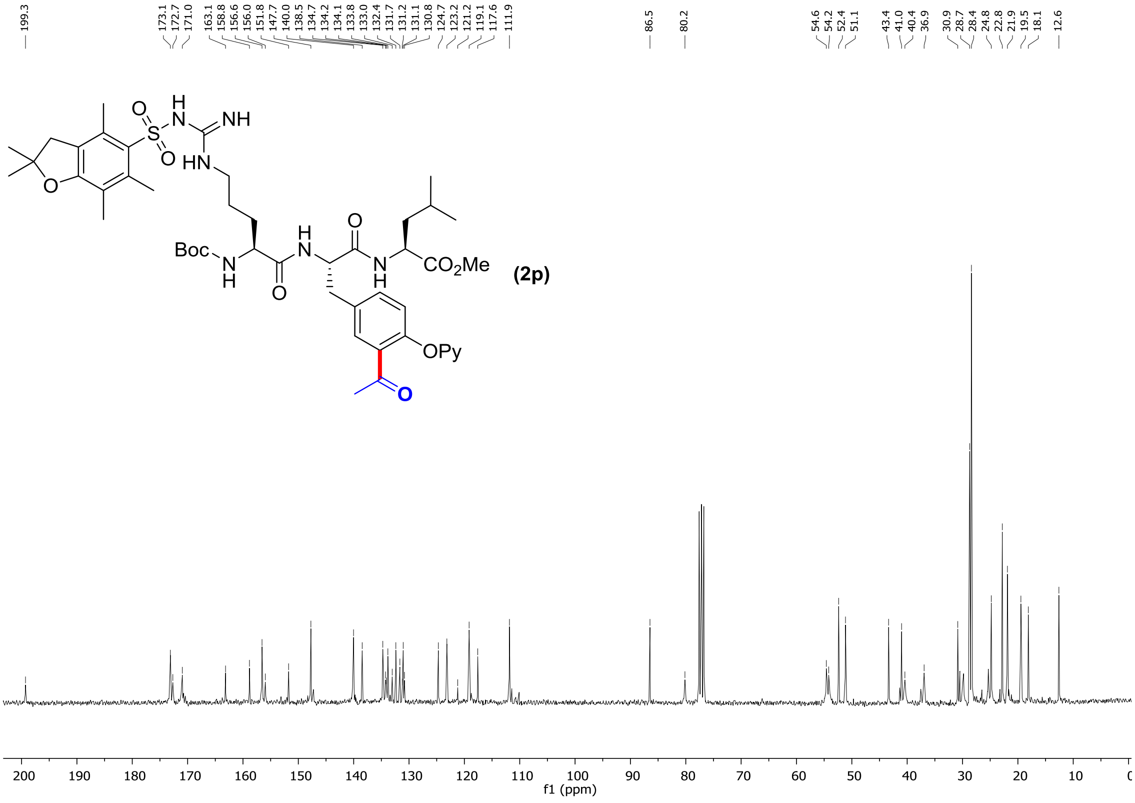

^{1}H NMR (400 MHz, $CDCl_3$)

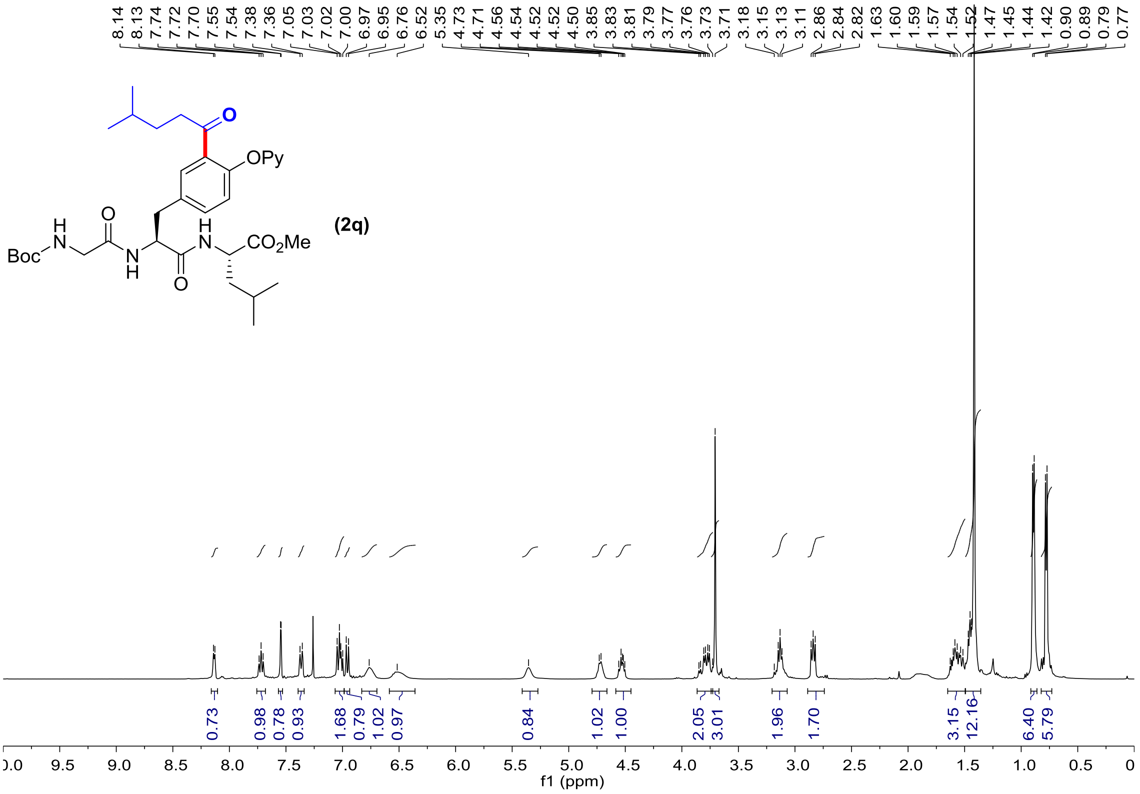

(2q)

^{13}C NMR (101 MHz, $CDCl_3$)

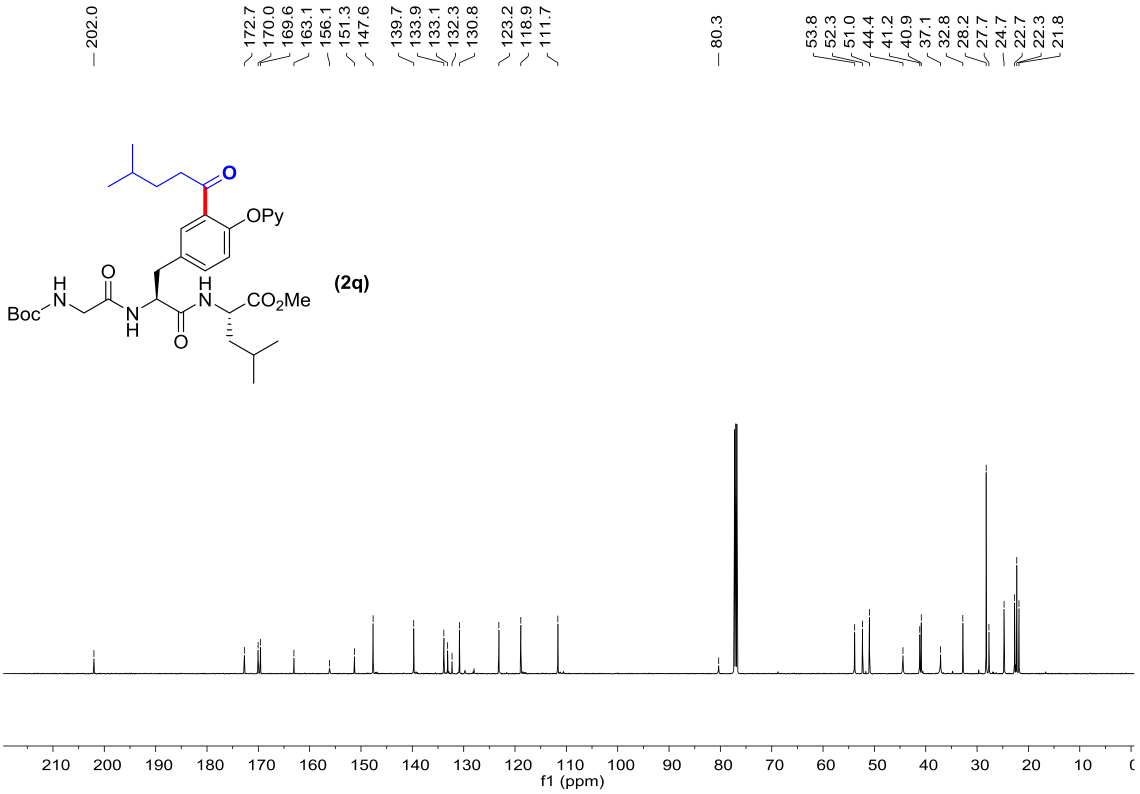

(2q)

^{1}H NMR (300 MHz, $CDCl_3$)

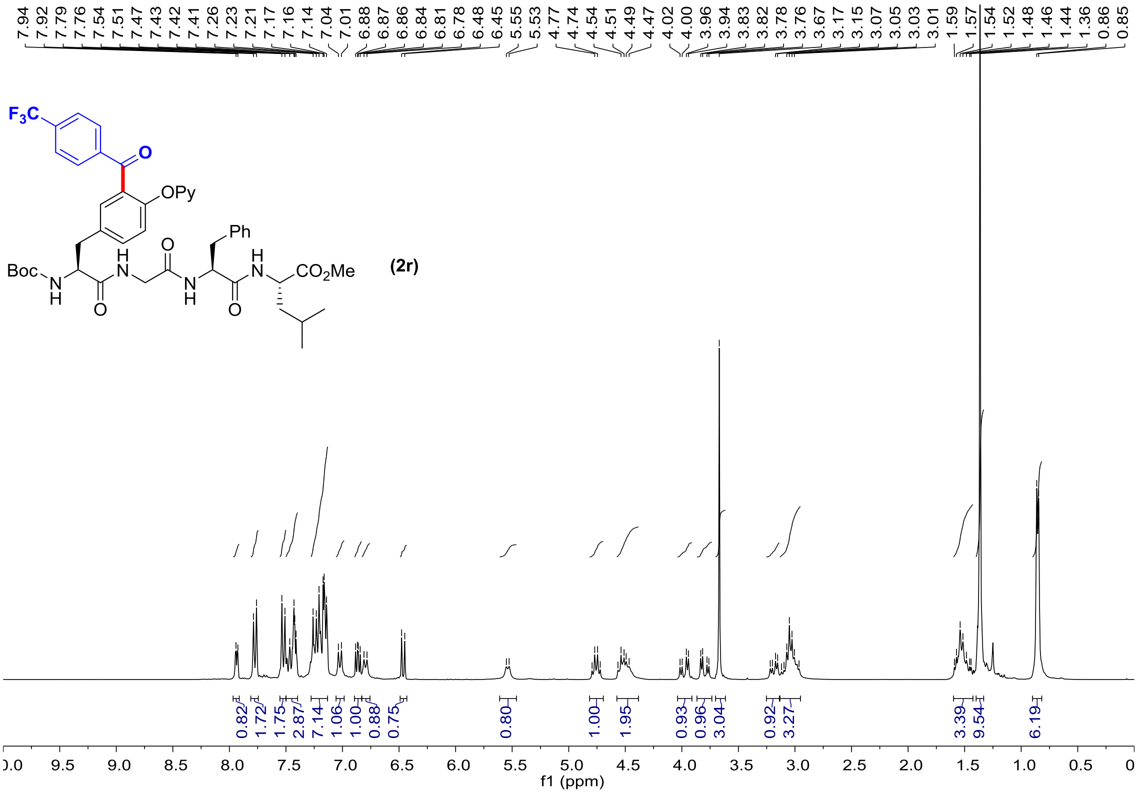

^{13}C NMR (75 MHz, $CDCl_3$)

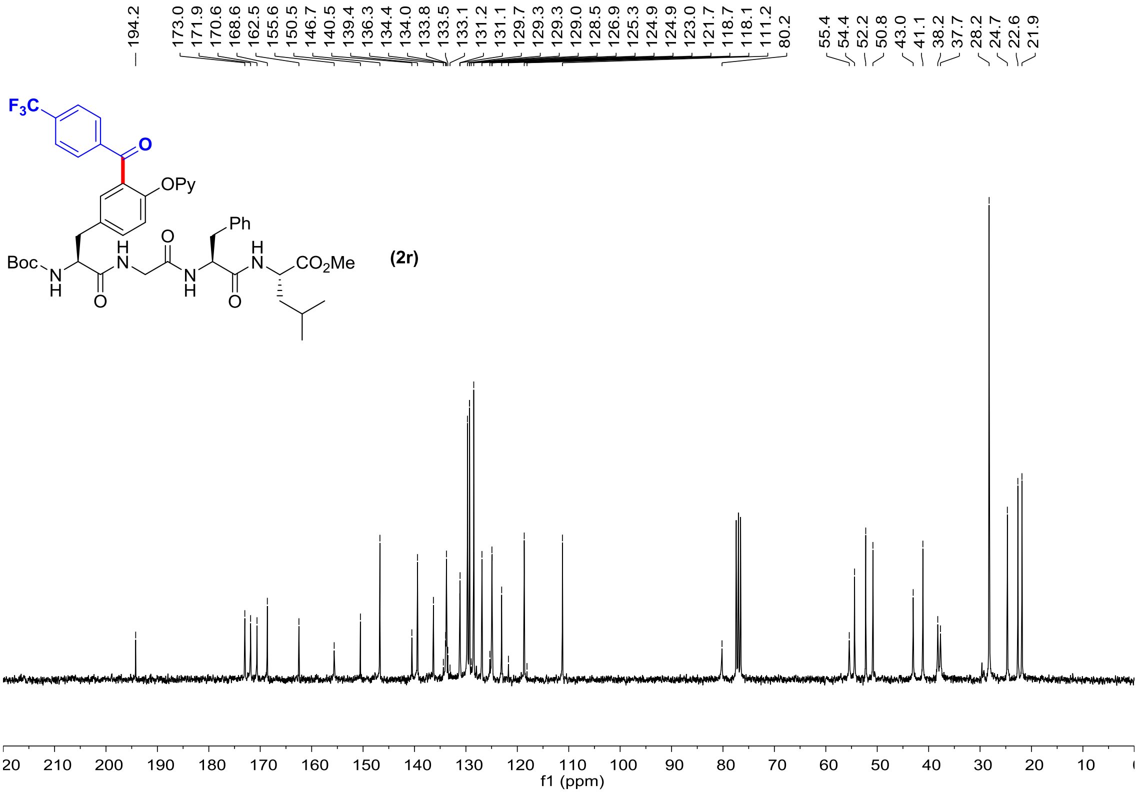

Supplement: Supplementary file 1 — ol1c02764_si_001.pdf [file ol1c02764_si_001.pdf]
